# Redefining tissue specificity of genetic regulation of gene expression in the presence of allelic heterogeneity

**DOI:** 10.1016/j.ajhg.2022.01.002

**Published:** 2022-01-31

**Authors:** Marios Arvanitis, Karl Tayeb, Benjamin J. Strober, Alexis Battle

**Affiliations:** 1 Department of Biomedical Engineering, Johns Hopkins University, Baltimore, MD 21211, USA; 2 Department of Medicine, Division of Cardiology, Johns Hopkins University, Baltimore, MD 21205, USA; 3 Department of Computer Science, Johns Hopkins University, Baltimore, MD 21211, USA; 4 Department of Genetic Medicine, Johns Hopkins University, Baltimore, MD 21205, USA

**Keywords:** eQTL, GWAS, colocalization, fine-mapping, tissue specificity

## Abstract

Uncovering the functional impact of genetic variation on gene expression is important in understanding tissue biology and the pathogenesis of complex traits. Despite large efforts to map expression quantitative trait loci (eQTLs) across many human tissues, our ability to translate those findings to understanding human disease has been incomplete, and the majority of disease loci are not explained by association with expression of a target gene. Cell-type specificity and the presence of multiple independent causal variants for many eQTLs are potential confounders contributing to the apparent discrepancy with disease loci. In this study, we investigate the tissue specificity of genetic effects on gene expression and the overlap with disease loci while considering the presence of multiple causal variants within and across tissues. We find evidence of pervasive tissue specificity of eQTLs, often masked by linkage disequilibrium that misleads traditional meta-analytic approaches. We propose CAFEH (colocalization and fine-mapping in the presence of allelic heterogeneity), a Bayesian method that integrates genetic association data across multiple traits, incorporating linkage disequilibrium to identify causal variants. CAFEH outperforms previous approaches in colocalization and fine-mapping. Using CAFEH, we show that genes with highly tissue-specific genetic effects are under greater selection, enriched in differentiation and developmental processes, and more likely to be involved in human disease. Last, we demonstrate that CAFEH can efficiently leverage the widespread allelic heterogeneity in genetic regulation of gene expression to prioritize the target tissue in genome-wide association complex trait loci, thereby improving our ability to interpret complex trait genetics.

## Introduction

Understanding the mechanisms that underlie genetic regulation of gene expression is crucial to explaining the diversity that governs complex traits. Large scale expression quantitative trait locus (eQTL) studies have been instrumental in identifying genetic variants that influence the expression of target genes and can be used to identify relevant genes for disease-associated genetic loci.^1^^,^^2^ This is particularly useful for the large fraction of disease loci in non-coding regions of the genome. However, the majority of disease-associated genetic variants have not yet been clearly explained by current eQTL data,^3^, ^4^, ^5^ frustrating attempts to use these data to comprehensively characterize disease loci.

One reported observation from recent studies of the genetics of gene expression is that *cis*-eQTLs often appear to be shared across different cell types and tissues.^6^, ^7^, ^8^ However, linkage disequilibrium (LD) within each locus along with the presence of multiple causal alleles within or between cell types may skew the quantification of sharing of genetic effects between tissues and impede our ability to identify causal variants. Indeed, recent research has demonstrated that multiple causal variants are often present in many eQTL and complex-trait-associated loci,^9^^,^^10^ suggesting that allelic heterogeneity may be more common than previously anticipated and underscoring the importance of disentangling causal signals in high-LD regions. These complex patterns could hinder the identification of regulatory effects for disease-associated genetic variants, potentially obscuring both the relevant cell type and target gene.

Here, we re-analyze tissue specificity of genetic effects in the presence of LD and allelic heterogeneity. We demonstrate that *cis*-eQTL effects appear to be predominantly tissue specific, according to methods that directly account for LD. In fact, eQTL loci often have multiple distinct signals across tissues in high LD, thus leading to inflated estimates of tissue sharing by traditional meta-analysis methods. Further, we propose a Bayesian method, CAFEH (colocalization and fine-mapping in the presence of allelic heterogeneity), that incorporates genetic association signal and LD structure across multiple traits, tissues, and studies together to improve the identification of causal regulatory variants across tissues and their relationship to disease loci. We show that eQTL tissue specificity is associated with signals of selection and disease relevance. That is, tissue-specific genes are under greater selective pressure, and tissue-specific eQTLs are more likely to colocalize with disease loci. Ultimately, we reveal that CAFEH can leverage *cis*-eQTL tissue specificity to effectively prioritize the target tissue and inform functional characterization of disease loci. Together, these data and CAFEH provide an improved framework for interpreting tissue specificity and interrogate disease mechanism.

## Material and methods

### Evaluation of tissue sharing in GTEx v8

The primary source of data for this analysis were eQTL summary statistics across 49 human tissues and cell types generated by the GTEx Consortium v8 release.^10^ We additionally used individual-level whole-genome and RNA sequencing by GTEx processed according to the GTEx v8 protocol.^10^ Metasoft^11^ analysis was performed as previously described in GTEx v8.^10^ To perform colocalization, we employed COLOC with the approximate Bayes method^12^ for each gene locus defined as all SNPs in a region within 1 Mb from the corresponding gene transcription start site. COLOC was performed in a pairwise manner between all 49 GTEx v8 tissues for all genes that were expressed in at least one tissue in GTEx v8. Priors for the different colocalization probabilities were set at p1 = 1 $\times \;10^{-4}$, p2 = 1 $\times \;10^{-4}$, p12 = 1 $\;\times \;10^{-6}$ according to the authors’ recommendation in the original COLOC paper.^12^ Colocalization was defined as a PPH4 ≥ 0.5 unless explicitly stated otherwise. Gene-tissue pairs that did not have a signal for an association with genotype in both tested studies (i.e., gene-tissue pairs with PPH3 + PPH4 < 0.5) were excluded from the analysis of tissue sharing.

Because COLOC evaluates the probability of colocalization at the locus level considering the locus signal as a whole, whereas Metasoft m-values work on the variant level and determine the probability for a given variant to be significant eQTL for a gene-tissue pair, in order to perform comparisons between the two, we defined that two tissues shared regulation for a given gene based on Metasoft if the m-value for the variant with the minimum eQTL p value across all GTEx tissues for that gene was ≥0.5 in both tissues.

COLOC by default assumes at most a single causal variant per locus. That assumption may influence the results in loci with multiple causal variants, biasing them against colocalization. Therefore, to test whether the substantial tissue specificity observed by COLOC was significantly influenced by that limitation, we repeated our colocalization analysis by using eCAVIAR,^13^ a software that relaxes the single-causal-variant assumption. A limitation of eCAVIAR is that it scales exponentially in the number of assumed causal variants, making inference of colocalization substantially slower as the number of variants increases. Consequently, even assuming at most two causal variants per locus, this analysis was not computationally feasible across all 35,848 genes and 49 tissues evaluated with COLOC. Therefore, we performed eCAVIAR assuming at most two causal variants per locus in a randomly sampled subset of 1,000 genes out of the 35,848 to evaluate the distribution of genes with shared and distinct regulation in different tissues pairwise. We defined that two tissues colocalize based on eCAVIAR if they share at least one variant with a minimum causal posterior probability in both tissues ≥0.5.

### Evaluation of tissue sharing between GTEx and other datasets

We subsequently evaluated tissue sharing between all 49 tissues in GTEx v8 and human tissues or cell types from Muther^14^ and eQTLGen.^15^ Specifically, for each of the three Muther tissues and cell types that *cis*-eQTL data are available (fat, skin, and lymphoblastoid cell lines), we performed pairwise colocalization analysis for all genes that were tested in both Muther and GTEx v8 between the corresponding Muther tissue and all 49 GTEx tissues. We plotted the colocalization posterior probability (PPH4) mean and 95% confidence intervals by using the subset of gene-tissue pairs that had an eQTL signal in both tissues based on the COLOC output (i.e., PPH3 + PPH4 ≥ 0.5). We followed the same procedure to test for colocalization between eQTLGen whole blood and all 49 GTEx tissues.

### Cell-type deconvolution in GTEx whole blood

We performed cell type deconvolution analysis in GTEx whole blood tissue by using CIBERSORT.^16^ Specifically, we ran CIBERSORT with default parameters by using as input the GTEx v8 whole blood expression in transcripts per million and the signature genes and average expression from a dataset of 22 circulating human immune cell types.^16^ We classified a cell type as estimable in GTEx if it had a corresponding CIBERSORT estimate > 0.05% in more than 5% of the GTEx whole blood samples.^17^ We were able to estimate cell-type proportions in GTEx for 15 different immune cell types by using the above method.

After obtaining the CIBERSORT estimates, we performed interaction QTL calling by using MatrixEQTL^18^ and the following linear model:

Y=intercept+a×cellcomposition+b×genotype+c×covariates+d×neutrophilpercent:genotype,

where Y is the processed gene expression, *genotype* is the genotype of the lead *cis*-eQTL SNP for that gene, *cell composition* is a matrix containing the cell-type proportions for each of the 15 estimable cell types in GTEx, covariates include sex, PCR, platform, 60 PEER factors, and five genotype PCs, and neutrophilpercent:genotype is the interaction term between the SNP genotype and the proportion of neutrophils in each GTEx sample. We evaluated statistical significance of the interaction effect estimate *d* by using a two-sided Wald test and performed Benjamini-Hochberg correction of the p values across tested genes. We then selected the genes that had a significant interaction QTL at different FDR thresholds and tested whether genes that had high posterior probability for non-colocalization between eQTLGen and GTEx whole blood (PPH3 > 0.9) would be enriched in genes with a significant interaction QTL compared to genes that had high PPH4 > 0.9 (suggesting that cell-type differences influence colocalization estimates between the two datasets).

### Simulations to evaluate COLOC performance

To evaluate the performance of COLOC under different underlying LD patterns and numbers of causal variants in each locus, we performed a series of simulations. To ensure we have a broad representation of LD structures in our simulations, we first computed LD scores for all variants in GTEx v8 whole-genome sequencing that passed the standard GTEx v8 filters with the ldsc software.^19^ Naturally, genes with higher LD score are expected to be found in regions with higher LD on average. We then split each gene that had a significant *cis*-eQTL in GTEx whole blood into LD quintiles based on the LD score of its top eVariant. Subsequently, we randomly sampled 20 different genes from each LD bin. For each gene we obtained genotypes for the corresponding locus by selecting the SNPs within 1 Mb from the gene’s transcription start site in four different datasets:

(1) GTEx whole blood,
(2) GTEx thyroid,
(3) 1000 Genomes Europeans, and
(4) 1000 Genomes Africans.

We evaluated three possibilities regarding the number of causal variants:

(1) there is a single causal variant that was selected to be the top *cis*-eVariant for the corresponding gene in GTEx whole blood;
(2) there are two causal variants, one of which is the top *cis*-eVariant for the corresponding gene in GTEx whole blood and the others are selected randomly among the remaining locus variants;
(3) There are five causal variants, one of which is the top *cis*-eVariant for the corresponding gene in GTEx whole blood and the others are selected randomly among the remaining locus variants.

For all configurations, we simulated gene expression by using the following linear model:

$$Y_{j}=\sum_{i=1}^{n}\left(b_{i}\times x_{ij}\right)+\varepsilon$$

ε∼N(0,0.9)

$$b_{i}∼N\left(0,\frac{0.1}{n}\right),$$

where $Y_{j}$ is the simulated expression for the $j_{th}\;$ individual, *n* is the number of causal variants, $b_{i}$ is the effect size for the $i_{th}\;$ causal variant, assumed to have a normal distribution with heritability (10/n)%, according to our prior knowledge on average *cis* heritability of gene expression,^4^ $x_{ij}\;$ is the genotype for causal variant *i* and individual *j*, and ε is the residual error term with a normal, zero-mean distribution.

For each run of the simulation, we simulated gene expression by using the above method for GTEx whole blood individuals and one of the four different genotype datasets listed above. After simulating gene expression, we then obtained simulated eQTL summary statistics for each variant in a locus by performing simple linear regression between the simulated gene expression and the variant genotypes. COLOC was performed on the simulated summary statistics between GTEx whole blood and each dataset with the same priors as outlined above for the analysis of real data. 100 independent simulations were performed for each dataset and causal variant configuration.

### CAFEH

CAFEH is a probabilistic model that performs colocalization and fine-mapping jointly across multiple traits. Let Y be an N×T matrix of measurements from N individuals in T traits. Let X be an N×G matrix of genotypes for each individual in G SNPs. We assume an additive genetic model

$$Y_{it}=X_{i}^{T}b_{t}+\epsilon_{i},$$

where $b_{t}$ is a sparse vector of effect sizes in trait t and $\varepsilon_{i}∼N\left(0,\tau^{-1}\right)$ is i.i.d. noise. We model $b_{t}$ as

$$b_{t}=\sum_{k=1}^{K}\varphi_{k}s_{tk}w_{tk}$$

$$w_{tk}∼N\left(0,\alpha_{tk}^{-1}\right)$$

$$s_{tk}∼Bernoulli\left(p_{0k}\right)$$

$$\varphi_{k}∼Categorical\left(\pi_{0}\right).$$

Similar to SuSiE,^20^ $b_{t}$ is written as a sum of components where each component captures the effect of a single causal variant. Here, $\pi_{0}=\left(\pi_{01},\;\ldots ,\;\pi_{0G}\right)$ is a vector with the prior probability that each SNP is the causal variant, and $\varphi_{k}$ is a one-hot vector of length G indicating the SNP selected in the $k_{th}$ component. We place a spike and slab prior on the effect sizes, parameterized as the product of a Bernoulli and normal random variable $s_{tk}w_{tk}$. Here, $p_{0k}$ gives the prior probability that the $k_{th}$ component is active (i.e., has non-zero effect) in each trait, and $\alpha_{tk}$ gives the prior precision of the effect size.

Intuitively, CAFEH enforces that all traits have zero effect at SNPs not selected by $\varphi_{1},\ldots ,\varphi_{K}$. While the model enforces that at most K SNPs have non-zero effect in each trait, a Bayesian treatment of $\varphi_{1},\ldots ,\varphi_{K}$ allows us to express uncertainty in *which* K SNPs have non-zero effect. In practice, we cannot distinguish between the causal SNP and other tightly linked SNPs included in the model; however, the posterior mass of each $\varphi_{1},\ldots ,\varphi_{K}$ will concentrate on groups of linked SNPs with shared association signal supported by the data. Thus, inference on $\varphi_{1},\ldots ,\varphi_{K}$ constitutes fine-mapping.

The choice of a spike and slab prior on the effect sizes of each component is motivated by the fact that we do not expect all causal variants to be shared across all tissues. With our parameterization, this can be seen easily; $\left(s_{t1},\ldots ,s_{tK}\right)$ are binary variables that select a subset of the K components to have non-zero effect in trait t. When $s_{tk}=0$, component k does not contribute trait t and the causal variant selected by component k is not considered causal in trait t. Conversely, when $s_{tk}=1$, component k will have non-zero effect in trait t and is considered causal. The sparsity induced by the spike and slab leads to straightforward colocalization; two traits, $t_{1}$ and $t_{2},\;$ colocalize in component k if they are both active in component k, that is $s_{t_{1}k}=s_{t_{2}k}=1.$^20^

To complete our model specification, we place priors on the variance terms for our effect sizes and residuals.

$$\alpha_{tk}∼Gamma\left(a_{0},b_{0}\right)$$

$$\tau_{t}∼Gamma\left(c_{0},d_{0}\right)$$

We emphasize the choice of giving each effect in each trait its own precision parameter $\alpha_{tk}$. While effects are modeled as normal, the magnitude of effects are free to vary across traits and causal variants. Thus, CAFEH does not place strong assumptions on the distribution of effect sizes across traits or causal variants.

When T=1, CAFEH reduces to SuSiE with a spike and slab prior on the effect sizes. CAFEH generalizes SuSiE by estimating causal variants across multiple traits jointly. This enables straightforward colocalization analysis and dramatically improves power to perform fine-mapping of shared causal variants by sharing information across multiple traits.

We refer to this form of the CAFEH model as CAFEH-G to emphasize that it is fit with individual-level genotype data.

### Fitting CAFEH from summary statistics

To facilitate the application of CAFEH to genome-wide association study (GWAS) with publicly available summary statistics, we implement a version of CAFEH, CAFEH-S, that can be estimated with summary statistics and a reference LD matrix by using the RSS likelihood.^20^ The RSS likelihood relates the coefficients of a multivariate regression to the effect sizes and standard errors of the marginal univariate regressions. Let ${\hat{\beta }}_{t}$ and $\hat{{\sigma_{t}}^{2}}$ be vectors of effect sizes and standard errors from a simple linear regression of phenotype t against a set of G SNPs. Let $\hat{R}$ be the sample LD matrix computed on X. Define $s_{i}^{2}=\left({\hat{\beta }}_{ti}^{2}/N\right)+{\hat{\sigma }}_{ti}^{2}$ and $\hat{S}$ a diagonal matrix with $i_{th}$ diagonal equal to $s_{i}.\;$ Up to a constant, the likelihood $p\left(Y_{t}|\;X,b_{t}\right)$ is equal to the likelihood of ${\hat{\beta }}_{t}$ under the model (Proposition 2.1 in Zhu and Stephens^20^):

$${\hat{\beta }}_{t}∼N\left(\hat{S}\hat{R}{\hat{S}}^{-1}b_{t},\hat{S}\hat{R}\hat{S}\right).$$

Thus, we can equivalently do inference with summary statistics. In practice, the sample LD matrix may not be available, and $\hat{R}$ will need to be estimated from a panel of reference genotypes.

### Variational inference for CAFEH

The exact posterior distribution $p\left(\left\{w_{tk}\right\},\left\{s_{tk}\right\},\left\{\varphi_{k}\right\},\left\{\alpha_{tk}\right\},\left\{\tau_{t}\right\}|Y,X\right)\;$ is intractable, so we approximate the posterior distribution by using variational inference. We select a family of distributions Q over the latent variables of the model that factorize as

$$q\left(\left\{w_{tk}\right\},\left\{s_{tk}\right\},\left\{\varphi_{k}\right\},\left\{\alpha_{tk}\right\},\left\{\tau_{t}\right\}\right)=\prod_{t=1}^{T}\prod_{k=1}^{K}q\left(w_{tk}|\varphi_{k},s_{tk}\right)q\left(s_{tk}\right)q\left(\alpha_{tk}\right)\prod_{k=1}^{K}q\left(\varphi_{k}\right)\sum_{t=1}^{T}q\left(\tau_{t}\right).$$

We perform coordinate ascent variational inference^21^ to find a member of this variational family that (locally) minimizes the Kullback Leibler (KL) divergence to the true posterior distribution. All updates can be written in closed form. Detailed derivation of the updates for CAFEH-G and CAFEH-S, as well as implementation and initialization details, are available in the supplemental methods, sections 2.4 and 2.3, respectively.

For CAFEH-S, to avoid costly matrix-vector multiplications at every iteration, we implement stochastic variational inference by using a Monte-Carlo estimate of the variational objective. Specifically, we approximate expectations over q(φ) by sampling. Details are available in supplemental methods, section 3.3.

### Setting hyperparameters

CAFEH users need to specify the number of components K and the $p_{0k},$ the prior probability, that each component is active in each phenotype. K can be set to a large value (e.g., 20, 100), which is an upper bound on the number of causal variants CAFEH can detect. Irrelevant components will not be assigned to phenotypes. Similar to SuSiE, unused components have their posterior mass spread over a large number of variants, so they do not significantly impact the posterior inclusion probabilities.

We conservatively choose a null initialization for CAFEH: the posterior means of all effects in all traits are initialized to 0 (that is $b_{t}=0$ for t=1,…,T) and the residual variance of trait t, $\tau_{t}^{-1},$ is initialized to the sample variance of trait t. We also initialize the prior effect size variance $\alpha_{tk}^{-1}=0.1$, which we recommend as a sensible default when running CAFEH with standardized genotypes and traits. However, good initialization of $\alpha_{tk}^{-1}$ depends on the scale of genotypes, traits, and the expected contribution of causal variants to trait variance.

### Simulations

We compare the performance of CAFEH-S and CAFEH-G to popular fine-mapping methods, including CAVIAR, FINEMAP, and SuSiE, and competing colocalization methods eCAVIAR and coloc. In all simulations, CAVIAR and eCAVIAR are fit with a maximum of two causal variants, SuSiE with a maximum of ten causal variants, and FINEMAP with a maximum of five causal variants. To better understand the impact of the spike and slab prior on fine-mapping, we run CAFEH in each simulated phenotype separately, which we denote as (SuSiE-SS). We evaluate fine-mapping methods by using the posterior inclusion probabilities (PIPs) returned by each model. We evaluate colocalization by using colocalization statistics of each method, PPH4, CLPP, and p_coloc for coloc, eCAVIAR, and CAFEH, respectively.

Gene expression data is simulated from real genotypes from 838 individuals in GTEx. We select 100 genes at random and take $X^{\left(i\right)}\;$ to be the genotype matrix for the G variants nearest the transcription start site (TSS) of gene i.

Simulated expression is controlled by four parameters: q, the number of causal variants in each phenotype; ρ, the percent variance explained by causal variants; $r_{\max}^{2},$ the maximum pairwise $r^{2}\;$ between causal variants; and T, the number of phenotypes simulated. Effect sizes are drawn from N(0,(1/p(1−p))), where p is the allele frequency of the causal variant. In order to control the signal strength, residual variance is added to achieve the proportion of variance explained by genotype ρ. Specifically, given a sampled vector of effects b, we set the residual variance $\tau^{-1}$ such that $\rho =Var\left(Xb\right)/(\tau^{-1}+Var\left(Xb\right))$. Within each simulation, traits are randomly assigned to one of two groups. For each group of traits, we sample a set of causal variants and then independently sample causal effect sizes for each trait in that group. We ensure that the two groups have distinct causal variants so that traits within the same group colocalize (i.e., share causal variants) while traits in different groups do not.

In the main set of simulations, we simulate T=4 traits, $r_{\max}^{2}=0.8$, taking all combinations of q=1,2,3 and ρ=0.01,0.05,0.1,0.2 with G=1,000. We also simulate more extensive allelic heterogeneity across a larger set of SNPs, simulating q=5,10 and ρ=0.2 by using all variants within 1 Mb of the TSS. eCAVIAR becomes computationally intractable on the larger simulation over the full *cis*-region, so for that simulation scenario, we run eCAVIAR by using only variants with *Z* score > 2 in at least one study.

To further investigate the value of fine-mapping shared causal variants jointly across traits, we generate simulations where causal variants are shared across an increasing number of studies. In particular, we simulate all combinations of T=4,8,16 traits randomly assigned to two groups with q=1,2,3 causal variants and ρ=0.05.

To demonstrate CAFEHs ability to perform fine-mapping and colocalization under more complex patterns of causal variant sharing, we simulate T=10 traits with q=10 causal variants, where causal variants are randomly assigned to each trait with probability 1/5. This simulation allows causal variants to be shared across arbitrary subsets of traits, while, on average, each trait has two causal variants and each variant is causal in two traits. This simulation is repeated across a range of signal strengths ρ=0.01,0.05,0.1.

While CAFEH is better able to fine-map variants that are shared across multiple phenotypes, it is also possible for CAFEH to represent multiple tightly linked causal variants with a single component, leading to false positive colocalization. To highlight this potential limitation, we generate simulations with T=4, q=1, and ρ=0.1. We vary the $r^{2}\;$ between the causal variant in each group of studies in the ranges (0,0.5),(0.5,0.7),(0.7,0.9).

To explore the robustness of CAFEH under different effect size distributions, we perform additional simulations where effect sizes for normalized genotypes are sampled from a mixture of 0 centered normal distributions with variance $\alpha^{-1}=0.01,0.05,0.1,0.5$. Residual variance is fixed at $\tau^{-1}=1.$ These simulations capture the scenario where a causal variant may be shared across multiple traits, but effect sizes differ in magnitude. We repeat this simulation, now sampling effect sizes from a mixture of point masses at $\sqrt{\frac{2}{\pi }\alpha^{-1}}$ for $\alpha^{-1}=0.01,0.05,0.1,0.5$. These values represent the expected magnitude of effect sizes under the normal mixture.

To evaluate the sensitivity of CAFEH to the setting of hyperparameters and initialization, we reevaluate to main simulations across a range of setting for $p_{0k}$ and initialization of the effect size precision parameters $\alpha_{tk}$.

We also generate simulations to evaluate the performance of CAFEH in the presence of causal structural variants (SVs). Using the same parameters as the main simulations, we generate simulations where causal variants are either SNPs or SVs. We then fit CAFEH and coloc by using only SNPs, only SVs, or SNPs and SVs.

### Redefining colocalization with CAFEH

By design, CAFEH outputs credible sets of variants in each component identified as active in at least one tested study for a locus. Because each locus can (and often does) have more than one active component, there are many ways in which to define colocalization between two studies. For our analyses, we chose the following two approaches (although other combinations can also be entertained).

(1) Colocalization in any component: defined as two studies sharing at least one component that is active in both studies with probability ≥ 0.5.
(2) Colocalization in the top component: defined as two studies sharing their top component. To select a top component for each study, we generated a weight for all variants in the 95% credible set of all active components in the study defined as follows:

$$weight=p_{active}\times \frac{effect\;size}{standard\;deviation\;of\;the\;effect},$$

where $p_{active}\;$ is the probability of the component being active in the study, effectsize is the effect size of the variant in the component, and standarddeviationoftheeffect is the standard deviation of that effect. We subsequently labeled as top component for each study the component that contains the variant with the maximum absolute value of the weight across all variants in the 95% credible sets of all components.

### Enrichment of CAFEH components in active regulatory elements

To evaluate the ability of CAFEH to identify causal variants in real data, we performed an enrichment analysis for variants in regulatory elements. Specifically, for each protein-coding gene-tissue pair in GTEx, we selected the credible set variant that has the maximum weight as defined above (see top component colocalization) for that tissue. For each tissue, we then compared the variants selected by this approach for each gene and evaluated for enrichment of those variants compared to a background of the variants that had the minimum *cis*-eQTL p value for association with the expression of each gene in that tissue. The approach ensures that the same number of variants are included in the test and background sets because one variant is selected for each gene-tissue pair. We then evaluated for overlap between these variant sets and active regulatory elements in corresponding tissues from Roadmap Epigenomics^22^ as defined in the main GTEx v8 paper^10^ and performed a Fisher’s exact test to evaluate enrichment of the test compared to the background variant set.

### Gene set enrichment analysis

To evaluate whether genes with highly shared or very tissue-specific regulation evaluated by CAFEH have distinct characteristics, we performed gene set enrichment analysis based on sets defined by Gene Ontology (GO)^23^ obtained by MSigDb.^24^ Our background set of genes consisted of all protein-coding genes in GTEx v8 that have a significant *cis*-eQTL in at least 20 tissues. We tested two sets of genes against the background: a highly colocalizing set, defined as the subset of background genes that are colocalizing in their top component in at least 20 tissues and a poorly colocalizing set, defined as the subset of background genes that are colocalizing in their top component in <10 tissues. Fisher’s exact test was used for the gene set enrichment analysis for each GO term and a Bonferroni correction was applied for multiple testing. GO terms that had a Bonferroni-adjusted p value < 0.05 were defined as enriched.

Similarly, the same sets of genes were evaluated for enrichment in sets of genes identified by OMIM as associated with human Mendelian diseases. We separated OMIM genes in two groups based on the underlying patterns of inheritance: genes with autosomal dominant inheritance and genes with autosomal recessive inheritance.

We also evaluated the relative selection status of the same set of genes by using loss of function observed/expected upper bound (LOEUF)^25^ and pLI^26^ as measures of selective pressures (lower LOEUF and higher pLI mean that the gene is more intolerant to variation). We compared the LOEUF and pLI distributions between the two gene sets and the background set by using a Wilcoxon rank-sum test.

### Using CAFEH to infer sharing and target tissue in GWAS loci

We used CAFEH to evaluate the degree of tissue sharing in causal genome-wide association study (GWAS) signals. We first evaluated 19 highly powered GWA studies of diverse traits from the UK Biobank.^27^ For each study, we performed colocalization by using CAFEH for all genome-wide significant loci. For each genome-wide significant locus, we assessed for colocalization genes for which the sentinel variant of the GWAS was also a genome-wide significant *cis*-eQTL for that gene in at least one of 49 GTEx v8 tissues. That process produced a set of genes to be tested for each locus. CAFEH was then run separately for each gene (because of concerns that joint inference across genes might be influenced by correlations between genes due to co-expression). Therefore, for each CAFEH run, the input was the tested GWA locus and the *cis*-eQTL summary statistics of one of the identified genes across all 49 GTEx v8 tissues. GTEx v8 LD was used as reference. For each CAFEH run, we extracted all components identified as active by CAFEH in at least one GTEx v8 tissue (at a threshold of 0.5). We then stratified those components in quartiles based on the number of GTEx v8 tissues that share the component that provides an estimate of how tissue specific each component is (from the most tissue-specific components on the first quartile to the most tissue-shared components in the fourth quartile). To assess whether tissue-specific components are more likely to be causal in GWAS than tissue-shared components, we evaluated the mean CAFEH posterior probability of a component’s being active (p_active) in the GWAS across all components in each quartile. To probe the overall association between the p_active in GWAS and the number of tissues that share said component, we performed a linear mixed model with the GWAS phenotype as the random effects term and the number of tissues sharing the component as the fixed effect. The model was the following: p_active ∼number_tissues_sharing_component + (1|trait). The addition of the random effects term in the linear model was considered necessary to account for a potential variation in the heritability and evolutionary pressure induced by the different tested traits, which may in turn influence the tested fixed effects relationship.

We then evaluated the converse question of whether components active in GWAS are more likely to be tissue specific compared to variants that regulate gene expression. For that, we used a published GWAS meta-analysis of coronary artery disease (CAD),^28^ which is a disease with high heritability that is known to be enriched for the liver and the arterial wall. For each gene within 1 Mb of each sentinel GWAS variant, we ran CAFEH-S jointly between the GWAS and all tissues in GTEx v8 for which that gene is expressed. From each CAFEH run, we selected the components that are active in the GWAS (based on a threshold of 0.5) and are also active in one of the tissues that are enriched in CAD heritability (either one of three artery tissues or the liver). We also performed CAFEH jointly across all GTEx v8 tissues for all protein-coding genes and selected the components that are active (at the same threshold of 0.5) in either one of three artery tissues or the liver. We generated boxplots of the number of tissues that share each of the selected components in a 2 × 2 factorial design (CAD or GTEx tissue for each of the following tissues: artery or liver). We then generated p values for the fixed effect term of linear mixed models of the form: number of tissues sharing component ∼Group_of_component (CAD versus GTEx tissue)+ (1|gene). The gene random effects term in that model was introduced to account for a potential variation in the tested fixed effects relationship based on the conservation status and relative importance of each gene.

In addition, we used the same data from CAFEH to assess the extent to which allelic heterogeneity in GWAS can be explained by eQTLs active in different tissues for the same gene. For each of the 19 tested GWAS traits, we counted the number of loci in which CAFEH identifies more than one causal component that also colocalize with eQTLs for a single gene in distinct tissues. We then plotted the ratio of the above number divided by either (1) all genome-wide significant loci, (2) genome-wide significant loci that have >1 active components based on CAFEH, or (3) all loci that have >1 active component in the GWAS and for which the sentinel GWAS variant is also an eQTL for at least one tissue.

In order to evaluate the effectiveness of CAFEH on prioritization of the target tissue in GWAS, we evaluated CAFEH’s performance in the same 19 highly powered GWA studies from the UK Biobank.^27^ CAFEH was performed as described above in the first paragraph of this section. We evaluated colocalization in any component or the top component (as defined previously) between the GWAS and the GTEx tissues for each genome-wide significant locus and we counted the number of loci for which each tissue colocalizes with the corresponding GWAS (each locus could be counted for more than one tissue if it colocalizes with multiple tissues). If a GWAS locus sentinel variant was a significant eQTL for more than one gene, the locus was included in multiple CAFEH runs (one for each gene) and the counts of colocalizing tissues across runs for that locus were aggregated such that each tissue was counted one time if it colocalized with that locus for at least one tested gene. The results of the aggregate colocalization counts for each phenotype were compared to the results of partitioned LD score (LDSC) regression for the same phenotype with tissue-specific genes from GTEx as previously described.^29^ The number of loci colocalizing with partitioned LDSC-enriched versus non-enriched tissues was compared with a linear mixed model with each phenotype defined as a random effect. In addition, for more detailed visualization of the results, we ranked each tissue based on the number of loci colocalizing in each GTEx tissue and plotted the ranks.

This analysis established that expected tissues based on partitioned LD score regression results and our prior knowledge of disease-specific pathogenesis can be identified and prioritized on the basis of a colocalization approach for the majority of traits. We should note that our expectation is that partitioned LD score regression, by virtue of the fact that it leverages the whole GWAS signal as opposed to genome-wide significant loci, may be more effective at this broad tissue prioritization. In contrast, our approach has the advantage of being able to identify putative target tissues at a locus resolution, therefore prioritizing tissues to be tested in downstream functional characterization of each locus. To demonstrate the effectiveness of CAFEH-S in identifying tissues in specific GWAS loci, we performed a case study with CAD as a complex trait. The choice of CAD was made on the basis of the fact that it is a complex trait with a highly heritable component and with published high-powered GWA meta-analyses.^28^ In addition, CAD has a multifactorial pathogenesis that involves multiple organ systems,^30^ thereby allowing for the possibility of different tissues’ being relevant in different loci. Lastly, because CAD was one of the first diseases to be studied in a GWAS approach, several downstream functional characterization studies have been undertaken for its significant loci, which provides gold-standard knowledge against which we can test the results of CAFEH-S.

To test CAFEH-S performance in CAD, we used a large-scale GWAS meta-analysis^28^ and performed a literature review to select loci that either (1) were in close proximity to a gene whose rare variants are known to predispose to atherosclerosis—the root cause of CAD—in a Mendelian fashion (*LDLR*, *APOE*, *APOB*, *PCSK9*, *ANGPTL4*, *LPL*, *ABCG8*, *CETP*) or (2) loci for which functional characterization followed by experimental validation studies have been performed linking the GWAS locus to a specific gene in a tissue or cell line.^2^^,^^31^, ^32^, ^33^, ^34^, ^35^, ^36^, ^37^, ^38^, ^39^, ^40^, ^41^ For each of the above loci, we ran CAFEH-S jointly with the GWAS and the GTEx v8 eQTL summary statistics for the putative target gene across all 49 GTEx v8 tissues. We also ran COLOC pairwise between the GWAS locus and each tissue for the target gene with the same parameters as described previously. We then compared the number of loci that colocalize in the target tissue by using any component or the top component colocalization according to CAFEH-S and those that colocalized with the target tissue according to COLOC, stratified by whether the GWAS sentinel variant is a genome-wide-significant eQTL for the target gene in the target tissue. We also plotted detailed results of colocalization on the basis of CAFEH-S.

### Participant data

This study used de-identified participant data from GTEx v8 and summary statistics from large-scale GWA studies. The study team never had access to individual identifiers. Participant consent was obtained as detailed in the original studies. GTEx v8 access was authorized by dbGaP after an official data access request.

## Results

### Colocalization reveals pervasive tissue specificity in gene regulation

**Figure 1:**
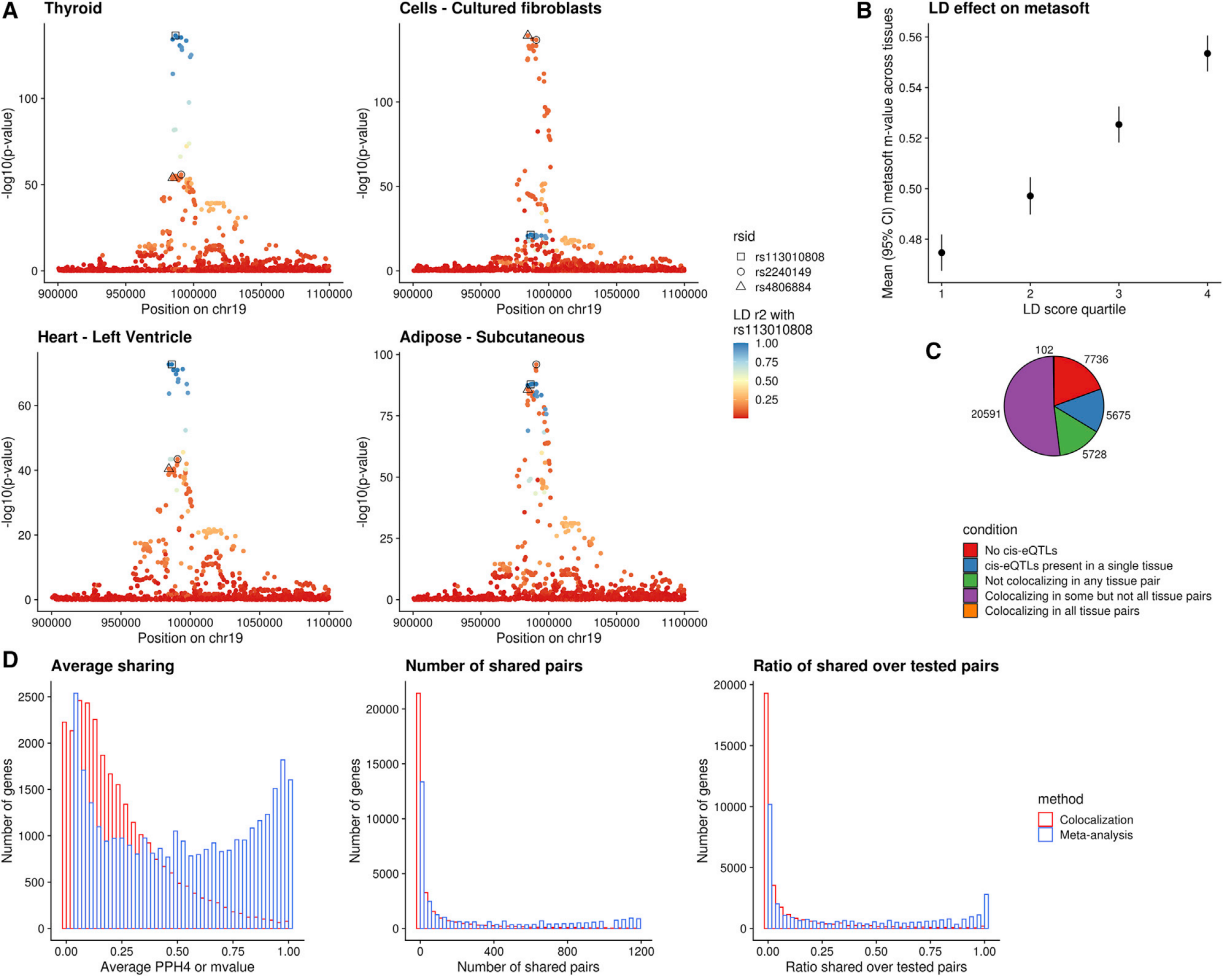
Colocalization provides evidence of extensive eQTL tissue specificity (A) Local Manhattan plots of *cis*-eQTLs for the *WDR18* gene in four tissues of GTEx v8. The plots reveal allelic heterogeneity between tissues (while thyroid and heart share the same pattern of genetic regulation, whole blood and fibroblasts have different causal variants). The three lead variants across the four tissues are colored on the basis of their LD with variant rs113010808 (lead variant in both thyroid and heart). All three variants have a Metasoft m-value of 1 in all four tissues. LD r^2^ between rs2240149 and rs4806884 is 0.79, while r^2^ between rs2240149 and rs113010808 is 0.15. (B) Metasoft m-values are positively correlated with LD. When the LD score quantile of the tested variant increases, the average m-value across tissues is higher. (C) Pie-chart of all 38,518 genes in GTEx v8 that are expressed in at least one tissue based on the tissue specificity of their eQTLs estimated by COLOC. (D) Histograms depicting patterns of sharing of genetic regulation between tissues based on COLOC and Metasoft. From left to right: histogram of mean PPH4 or m-value between tissue pairs for each gene; histogram of number of tissue pairs with shared *cis*-eQTLs for each gene; and histogram of the ratio of shared tissue pairs divided by the tissue pairs in which the gene is expressed. COLOC reveals more profound tissue specificity.

Previous analyses of *cis*-eQTL effect sharing across tissues have employed meta-analytic strategies that aggregate the association signals from multiple tissues.^6^^,^^10^^,^^11^^,^^42^ A crucial pitfall of these analyses lies in handling LD. Specifically, if two distinct causal regulatory *cis*-eQTL variants acting in separate tissues are in even moderate LD with each other, those variants often falsely appear to be active in both tissues, boosting each-other’s association signal and providing an often false, high estimate of eQTL sharing between tissues (example, Figure 1A). Indeed, tissue sharing statistics reported by the GTEx Project,^10^ quantified by Metasoft^11^ m-values, are strongly correlated with LD score (Figure 1B), indicating tissue sharing estimates are likely to be inflated by LD. This association remains after controlling for gene density and distance to the nearest transcription start site (Figure S1).

We performed an alternative analysis of tissue sharing among *cis*-eQTLs across 49 human cell types and tissues in GTEx v8^10^ using a colocalization approach (COLOC^12^) that explicitly incorporates LD information to overcome the confounding issues observed with other approaches. Colocalization has been commonly used for assessing the causal overlap between eQTLs and GWA studies but infrequently used for assessing relationships between eQTLs.^43^ Our analysis revealed that for the majority of genes, distinct causal variants are likely to be responsible for the *cis*-eQTL signals in different tissues (Figures 1C and 1D), and there was far less sharing than reported by meta-analysis approaches. Because COLOC is limited by the assumption of a single causal variant per tested region, we also performed another analysis with a second LD-aware colocalization method, eCAVIAR,^13^ allowing up to two causal variants per region. Because eCAVIAR is computationally expensive, it was run on a subset of 1,000 randomly sampled genes. These results confirmed the pattern of widespread tissue specificity in regulation of gene expression (Figures S2A and 2B).

**Figure 2:**
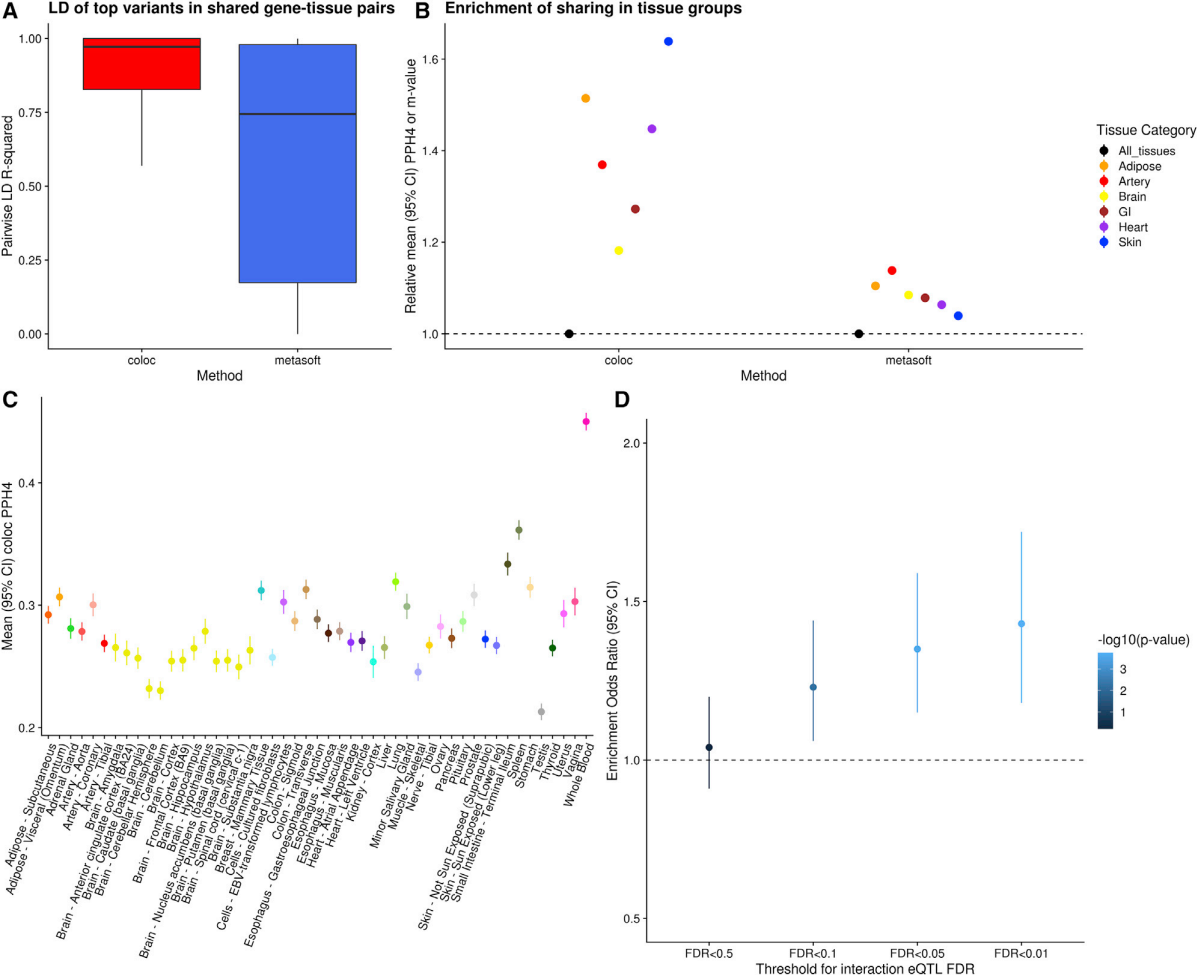
Colocalization is superior to other methods that don’t account for LD (A) Boxplots of the LD between the top variants per gene in tissue pairs that colocalize based on COLOC (in red) or Metasoft (in blue). (B) Average sharing between groups of biologically similar tissues in GTEx v8 based on COLOC or Metasoft. Values are normalized by dividing with the mean across all gene-tissue pairs. (C) Average eQTL sharing between eQTLGen whole blood and all GTEx v8 tissues for genes that have an eQTL in both datasets, using COLOC PPH4. (D) Odds ratio of enrichment for non-colocalizing genes (PPH3 > 0.9) compared to colocalizing genes (PPH4 > 0.9) between eQTLGen whole blood and GTEx whole blood. The odds ratios are stratified by the false discovery rate of interaction with cell type composition, measured as the interaction between the lead eQTL variant for that gene in GTEx v8 and the relative proportion of neutrophils (the most common whole blood cell type) in the GTEx sample.

We found that gene-tissue pairs were identified as shared by Metasoft even when they had distinct top *cis*-eQTL variants in only moderate LD, whereas for colocalization, sharing was identified only when the lead variants were identical or in high LD with each other, more likely tagging the same causal effect (Figure 2A). Further, using colocalization, tissues with similar origin had a higher degree of sharing across genes, whereas with meta-analysis this expected pattern of sharing was far weaker, as evaluated across the full range of PPH4 or m-values, respectively (Figure 2B and Figure S3). We should note that the overall degree of tissue sharing naturally changes depending on the selection of different thresholds for each method. For colocalization, even a remarkably lenient PPH4 threshold of 0.1 reveals substantial tissue specificity (Figure S4). For Metasoft, higher m-value thresholds do shift to a more tissue-specific pattern (Figure S5A) but primarily by selecting for eQTLs with a stronger p value in each tissue (Figure S6). Additionally, even at more stringent Metasoft thresholds, it does not identify the genes that appear to share effects through colocalization (Spearman r between COLOC PPH4 and Metasoft m-value = 0.57). Specifically, shared gene-tissue pairs identified by colocalization continue to show stronger LD between their lead variants compared to those identified as shared by Metasoft regardless of chosen threshold (Figure S5B), suggesting more LD artifacts with Metasoft regardless of threshold. The observed *cis*-eQTL tissue specificity also extends to datasets with larger sample size, such as the eQTLGen^15^ and Muther^14^ consortia (Figure 2C, Figure S7), suggesting that it reflects true tissue specificity as opposed to an artifact of low power, batch effects or sequencing approach. We noted that estimates of sharing in bulk tissue samples with cellular heterogeneity are affected by cell type composition variability between different datasets. Specifically, genes with a strong posterior probability for separate, independent signals between eQTLGen and GTEx whole blood tissue (PPH3 > 0.9) were enriched for cell-type interaction QTL signals compared to genes with colocalization between the two datasets (PPH4 > 0.9) (Figure 2D).

### CAFEH: A Bayesian method for colocalization and fine-mapping across multiple studies

The observed pervasive tissue specificity across *cis*-eQTLs when accounting for LD effects underscores the need for approaches able to probe the full spectrum of allelic effects across tissues and traits. Existing methods for colocalization, despite accounting for LD better than meta-analysis approaches, have several known limitations that preclude full exploration of tissue-specificity and causal variant sharing. First, most existing colocalization methods require manual specification of the number of causal variants for each locus, and those that allow for more than one causal variant become computationally intractable when many causal variants are specified, thereby substantially limiting evaluation of allelic heterogeneity. Further, even when two studies have the same underlying LD, most colocalization methods underperform in regions of high LD in a manner biased against reporting colocalization.^13^ Nonetheless, we should note that this bias is not enough to account for the observed *cis*-eQTL tissue specificity, as the evidence of pervasive tissue specificity well beyond that reported by meta-analysis methods remains present across different underlying LD structures (Figure S8). Last, most existing methods generally perform pairwise comparisons between studies, therefore failing to aggregate evidence from multiple studies or tissues jointly. All these limitations are highlighted in simulations we performed across a range of chosen number of causal variants, underlying LD structures, and different populations assessed (Figures S9 and S10), which show suboptimal performance of colocalization in the presence of allelic heterogeneity and high LD by COLOC.

**Figure 3:**
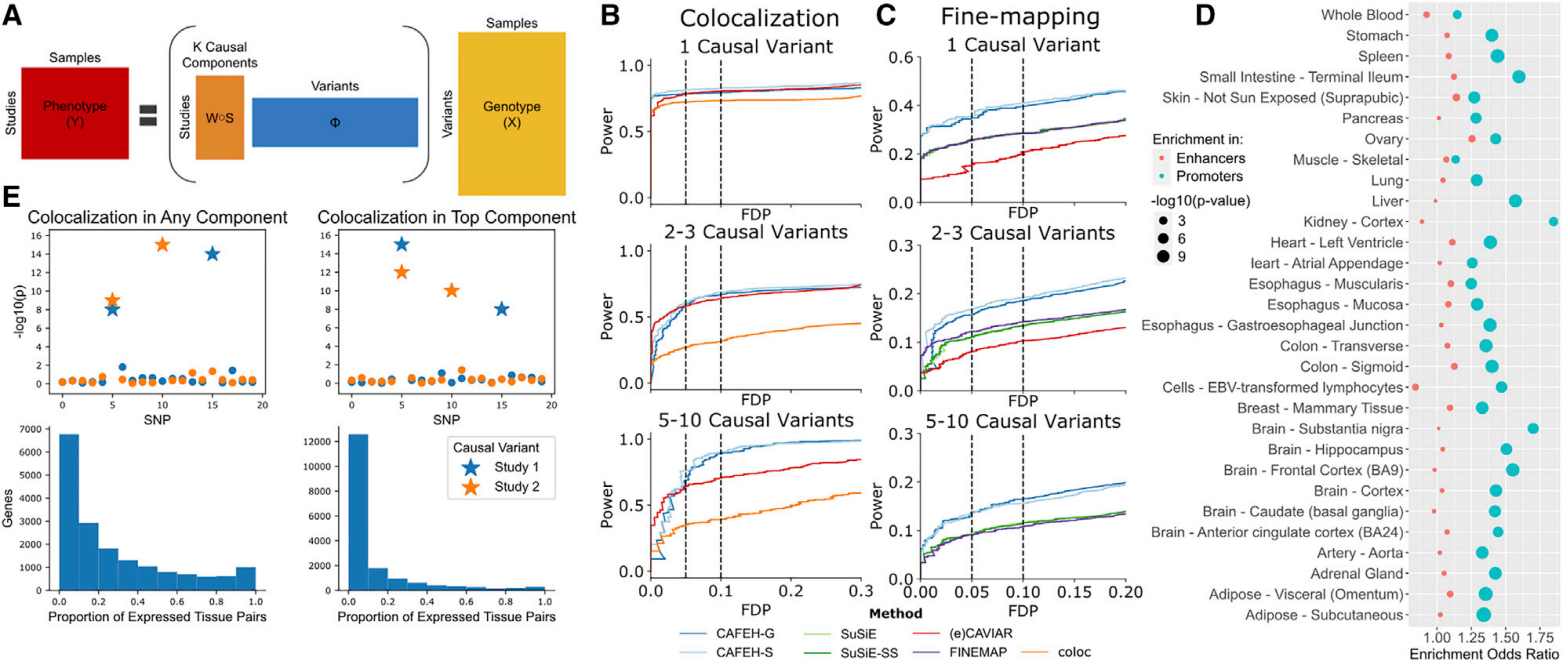
CAFEH GTEx and simulations (A) A schematic representation of CAFEH. CAFEH can be viewed as a sparse regression with a shared set of causal variants across all studies. Entries of W are modeled with a spike and slab prior, so each study uses a subset of causal variants. (B) Proportion of false discoveries (FDP) versus proportion of true positives (Power) across a range of colocalization thresholds. (C) FDP versus Power across a range of thresholds of the posterior inclusion probability in competing fine-mapping methods. (D) Enrichment of the top CAFEH variant of each gene in promoter (teal) and enhancer (red) elements in matched Roadmap cell-types relative to top eQTL variants. (E) Redefining colocalization with allelic heterogeneity. Top: representation of colocalization in any or the top component. Stars represent causal variants in each study. Bottom: Proportion of tissue pairs colocalizing in any or top CAFEH components at a 0.5 threshold.

To overcome the limitations of existing approaches and to robustly perform fine-mapping and colocalization in the presence of multiple causal variants across many studies, we developed CAFEH (colocalization and fine-mapping under allelic heterogeneity). CAFEH is a probabilistic model that fine-maps causal variants and estimates their effect sizes and pattern of sharing across multiple studies. CAFEH identifies a set of causal components across all tested studies, tissues, or traits that explain the observed association signals. Each component represents a single underlying causal variant. As a result of LD, we are often unable to pinpoint the exact causal variant that produces the observed signal for each component and therefore CAFEH outputs a credible set of variants for each component. Depending on availability of individual-level genotype and phenotype data, we developed two versions of the method, CAFEH-G (Figure 3A), when individual-level data are available, and CAFEH-S, which can be applied with summary statistics alone. CAFEH is fit with a fast variational approximation, which shows strong concordance with the exact model (Figures S11 and S12)

We explore the performance of CAFEH relative to other popular colocalization and fine-mapping methods through realistic simulations across a range of signal strengths and genetic architectures (material and methods). CAFEH performs similarly to existing methods when those methods’ more restrictive assumptions about the number of causal variants are satisfied and demonstrates improved power to detect colocalization in the presence of more extensive allelic heterogeneity (Figure 3B and Figure S13). In contrast to CAVIAR and eCAVIAR, CAFEH is tractable for large K, and thus avoids a serious issue of model misspecification. CAFEH is also able to dramatically improve fine-mapping across the range of simulations (Figure 3C). Furthermore, compared to SuSiE, CAFEH’s 95% credible sets are smaller and detect a higher proportion of causal variants (Figure S14). These improvements in fine-mapping over single-trait methods demonstrate the advantage of leveraging association signal across studies; this effect becomes even more pronounced when the number of traits sharing a causal variant is varied from 1–12 (Figure S15). Those results are similar in the presence of structural variation, provided that the actual causal variants are included in the analysis (Figure S16).

CAFEH can perform colocalization and fine-mapping robustly even when the distribution of effect sizes varies across traits and causal variants (Figures S17 and S18) and when patterns of causal variant sharing are more complex (Figure S19). We observe an improved performance of CAFEH compared to existing methods in the presence of more than one causal variant per locus. Given a single causal variant, CAFEH has better performance on fine-mapping but slightly lower performance for colocalization. We should note that even though CAFEH substantially improves our ability to assess allelic heterogeneity compared to existing methods across a range of LD thresholds, when LD is very high (r^2^ > 0.7) between two causal variants, CAFEH is unable to distinguish between them and assigns them in a single causal component (Figure S20). CAFEH’s objective is non-convex and is thus optimized to a local maximum. Despite this, we find that our default initialization performs well in practice, and among multiple initializations, the evidence lower bound (ELBO) maximizing initialization performs well (Figure S21).

### CAFEH corroborates the tissue specificity of *cis*-eQTLs

Importantly, when tested across 49 GTEx v8 tissues, CAFEH-G recapitulates the pervasive tissue specificity in genetic regulation of gene expression (Figure 3D and Figure S22–S24). Although, as expected due to eQTL detection power, the colocalization estimates across tissues are influenced by the expression level of the corresponding gene across tissues and lowly expressed genes have a bias toward no colocalization, eQTL tissue specificity is widely prevalent across all quintiles of median expression (Figure S25). Moreover, similar to our analyses with COLOC, CAFEH supports our findings of eQTL tissue specificity in eQTLGen (Figure S26) and the contribution of cell-type proportions on bulk tissue colocalization estimates (Figure S27) that we observed with COLOC.

Importantly, by identifying allelic heterogeneity within each locus, CAFEH allows us to redefine colocalization on the basis of different patterns of sharing of causal variants. For example, two studies may share all causal variants from either study, a very stringent definition of colocalization. Alternatively, they may share their most strongly associated causal component, or any subset of their active causal genetic components, a less stringent but still potentially informative form of colocalization (Figure 3E). Using the output from CAFEH, these and other customized criteria may be applied to define colocalization by each user depending on the particular goals of their study (supplemental methods). Notably, CAFEH-G reveals that the majority of genes have more than one causal variant per tested tissue (Figure S28). Similarly, when tested across all 49 GTEx v8 tissues, most genes have five or more total different causal variants influencing their expression among all tissues (Figure S28). Lastly, causal variants fine-mapped by CAFEH in each tissue are enriched for promoter elements in the corresponding tissues as defined by Roadmap chromHMM^22^ compared to top eQTL variants for the corresponding genes (Figure 3C). We should note that by nature of its joint cross-tissue inference, CAFEH prefers components that have evidence for an active signal in multiple studies and hence has a slight bias toward colocalization. However, that is offset by the increase in power that the joint inference allows, hence improving overall accuracy in both fine-mapping and colocalization (Figures 3D and 3E and Figure S13). We should also note that despite this potential bias toward sharing, CAFEH still reveals substantial evidence for tissue specificity of *cis*-eQTLs.

### Disease relevance, function, and selective pressure for tissue-specific eQTLs

**Figure 4:**
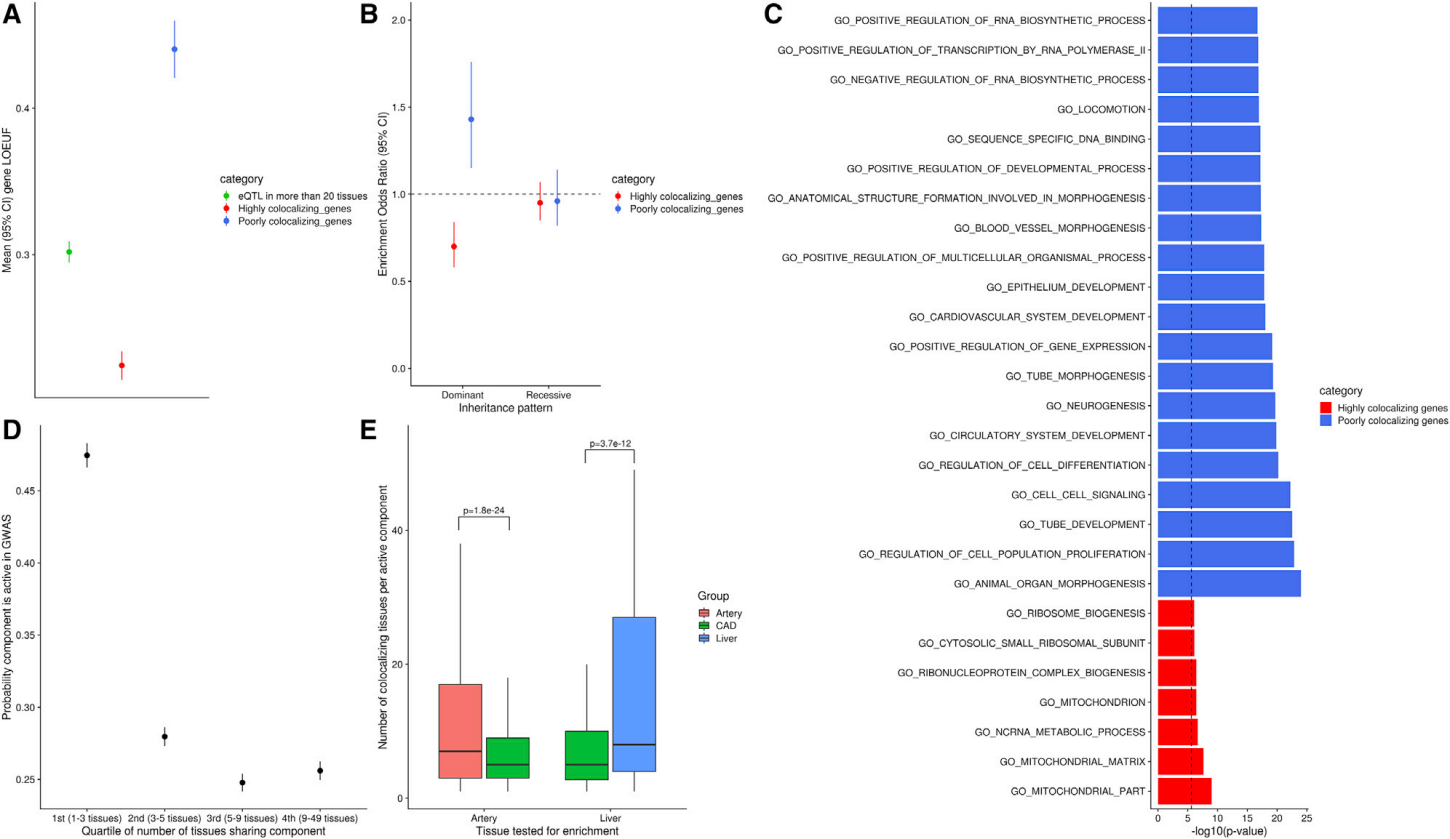
Characteristics of genes based on the degree of eQTL tissue sharing (A) Average loss of function observed/expected upper bound (LOEUF) between genes that are colocalizing in at least 20 tissues (highly colocalizing) and those that colocalize in less than five tissues (poorly colocalizing), comparing only genes that have an eQTL in at least 20 tissues. Colocalization was defined as sharing of the top causal component based on CAFEH. (B) Enrichment in genes whose rare variation is associated with autosomal dominant or recessive human diseases based on OMIM (left) between highly and poorly colocalizing genes. (C) Gene Ontology enrichment analysis of genes that are colocalizing in at least 20 tissues (highly colocalizing) and those that colocalize in less than five tissues (poorly colocalizing), comparing only genes that have an eQTL in at least 20 tissues. Colocalization was defined as sharing of the top causal component based on CAFEH. (D) Mean (95% CI) of probability of a *cis*-eQTL component’s being active in GWAS (as defined by CAFEH-S) stratified by the quartile of the number of 49 GTEx tissues that share the given eQTL. The figure presents aggregate results of 19 diverse GWAS traits from the UK Biobank. For each GWAS, we jointly run CAFEH-S between the GWAS summary statistics and GTEx v8 eQTL summary statistics among genes for which the sentinel variant of a genome-wide significant disease locus is also a genome-wide eQTL in at least one tissue. We then evaluate all components that are active in at least one GTEx tissue. Overall, there is a strong inverse association between the posterior probability of a component being active in the GWAS and the number of tissues that colocalize with said component (linear mixed model p = 3.3 × 10^−160^). (E) Boxplots of the number of tissues that share an active component identified by CAFEH, among components that are either active in a CAD GWAS and a GTEx tissue *a priori* believed to be relevant for CAD heritability (either artery on the left or liver on the right) among all genes within 1 Mb of genome-wide significant CAD locus, compared to components that are active only in a GTEx tissue (artery or liver) among all protein-coding genes.

We observed that genes whose genetic transcriptional regulation is predominantly tissue specific as defined by CAFEH have different characteristics than those whose regulation is shared across tissues. First, patterns of selective pressures were different between the set of genes that are highly shared between tissues and those that are predominantly tissue specific, restricting to genes that have a significant *cis*-eQTL in at least 20 tissues each. Specifically, tissue-specific genes were found to be more variation intolerant as measured by LOEUF^25^ and pLI^26^ compared to background genes, which were in turn more intolerant compared to the subset of genes with shared genetic regulation (Figure 4A and Figure S29), a phenomenon present regardless of the underlying strength of the eQTL association (Figure S30). In parallel, we observed that poorly colocalizing genes were enriched for participation in human diseases with dominant inheritance patterns as defined by OMIM^44^ (Figure 4B). Further, GO enrichment analysis^4^ demonstrated that poorly colocalizing genes were enriched in pathways related to development, differentiation, cell-adhesion, and transcription factor activity, suggesting that these pathways are critical to the differences between cell types in humans despite being broadly expressed and genetically regulated in many tissues. In contrast, highly colocalizing genes were enriched for mitochondria and the cytosolic ribosome, cellular structures that are abundant in all tissues and cell types (Figure 4C and Table S1).^44^ We then showed that causal eQTL components shared by very few tissues are more likely to be active in GWAS than those shared by multiple tissues (Figure 4D). Conversely, GWAS causal components that are also eQTLs in known target tissues are more likely to be tissue specific compared to general eQTL causal components in those tissues (Figure 4E). Further, we discovered that in a large proportion of GWAS loci that appear to have more than one causal variant according to CAFEH, the GWAS components colocalize with distinct tissues for the same gene, suggesting that eQTLs in different contexts or cell types may each capture effects on complex trait pathophysiology (Figure S31). These results are consistent with our finding of greater selective pressure for genes with tissue-specific regulation compared to tissue-shared genes and jointly suggest that exploration of cell-type-specific and potentially context-specific eQTLs could provide an explanation of the effects of GWAS variants that lack a colocalizing signal in currently available eQTL datasets.

### Identification of tissues of interest for disease loci

**Figure 5:**
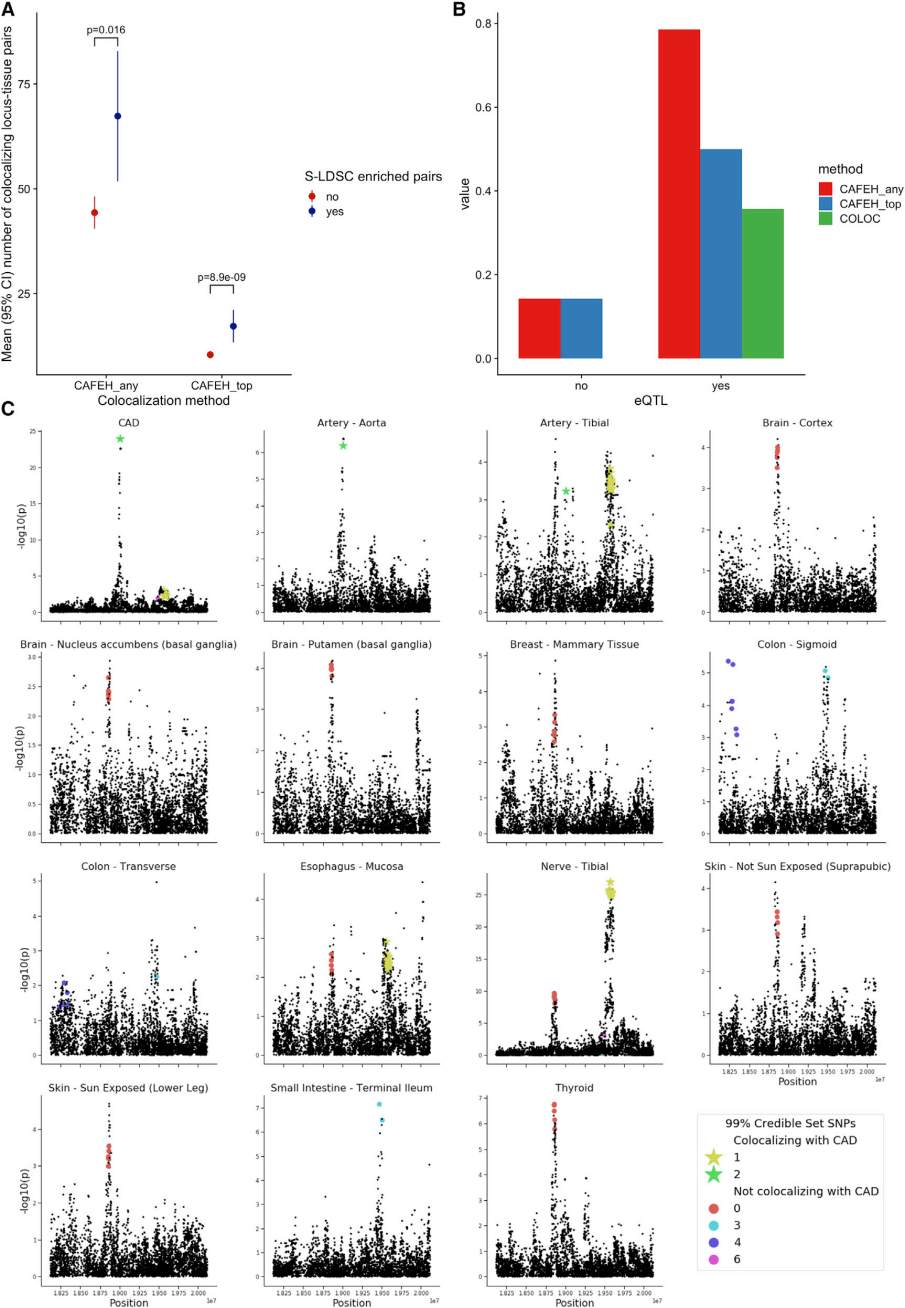
CAFEH identifies the target tissue in GWAS (A) Number of colocalizing locus tissue pairs in 19 diverse GWAS traits from the UK Biobank. Colocalization is defined on the basis of any component or top component in CAFEH and the counts are colored on the basis of whether the corresponding tissues are enriched for that trait by partitioned LD score regression. (B) Case study of loci in a large CAD GWAS that have an established target tissue. The bars represent the proportion of loci colocalizing in the known target tissue with any of three different methods (CAFEH colocalization in any component, CAFEH colocalization in the top component, COLOC). The results are stratified on the basis of whether or not the lead variant in the locus is a genome-wide significant *cis*-eQTL for any gene in at least one tissue in GTEx v8. We see that CAFEH outperforms COLOC and can prioritize the target tissue in most cases when an eQTL signal is present. (C) Example of CAFEH revealing allelic heterogeneity and identifying the target tissue in the TWIST1 CAD locus. CAFEH identifies a dominant component for the CAD GWAS in that locus with a 99% credible set that consists of a single variant (rs2107595). The same component is shared by artery tissue in GTEx v8. Recent CRISPR in human arterial smooth muscle cells confirmed its effects in influencing the expression of TWIST1 in that tissue.

The underappreciated yet extensive tissue specificity in *cis*-eQTLs should also in theory provide a basis to probe the tissue of interest in GWAS loci for a number of complex traits. Indeed, we showed that CAFEH-S accurately prioritizes the target tissue for a number of diverse traits from the UK Biobank and the tissue prioritization results agree with stratified LD score regression estimates applied to the same traits (Figure 5A, Figure S32). Next, we evaluated whether CAFEH-S is able to prioritize the correct tissue in GWAS variants where the active tissue is known. To do that, we performed a case study in CAD. We selected CAD as an example of a complex trait with substantial heritability^28^ and a pathophysiology that involves a variety of different organ systems.^45^ We assessed the performance of CAFEH-S in identifying the causal tissue for all CAD loci that have either been subjected to experimental validation of the causal tissue via genome editing or loci in which the closest gene is an established CAD gene with a known tissue-specific mechanism of action. CAFEH-S colocalizes with the correct tissue in those loci 80% of the time and outperforms COLOC when a significant eQTL signal is present, demonstrating that the approach is effective in most situations (Figures 5B and 5C, Figure S33) when highly powered GWAS and corresponding tissue eQTL summary statistics are available. Naturally, although the method identifies colocalization even in weak (non-genome-wide significant) eQTL signals (see SMAD3 locus in Figure S33), it is unable to colocalize with the target tissue when the eQTL signal is absent. We should note that of six loci without a genome-wide significant eQTL signal, three (ANGPTL4, APOE, LDLR)contain a coding variant of the corresponding target gene in high LD (R2 > 0.6) with the sentinel SNP, which may suggest their effects on phenotype may not be mediated by gene expression.

## Discussion

Our study has important implications in the interpretation of complex trait genetic association signals. First, we showed that genetic regulation of gene expression is much more tissue specific than previously appreciated and demonstrated that tissue-specific eQTLs are more likely than tissue-shared eQTLs to be regulating complex traits. This finding suggests that the lack of a colocalizing eQTL signal observed for the majority of non-coding genomic loci in large scale GWA studies to-date^1^^,^^42^^,^^46^ could be partially explained by the inability of most existing colocalization approaches to fully account for allelic heterogeneity, which can lead to inaccurate estimates of colocalization and therefore hinder our ability to understand GWAS signals.

Second, we proposed CAFEH, software that outperforms existing approaches for colocalization and fine-mapping when more than one causal variant is present in each tested trait and allows for multi-trait and multi-tissue estimation of allelic heterogeneity. CAFEH decomposes the genetic association signal into individual components, and each component corresponds to a single causal variant. Unlike existing methods,^12^^,^^47^ CAFEH does not rely on *a priori* knowledge of the number of causal variants in each genomic locus and is computationally tractable even for a large number of causal signals. In practice, this is achieved by setting an arbitrarily high number of causal components for each CAFEH run (in our study, we used 20 components per locus). The algorithm then automatically removes any additional components beyond the number needed to explain the tested association signals. In addition, by leveraging genomic association signals across multiple traits and studies, CAFEH can boost the power and accuracy of both fine-mapping and colocalization as we showed in simulations. The approach of jointly evaluating colocalization across different traits has been shown to improve colocalization estimates in prior work.^48^^,^^49^ CAFEH goes further by allowing users to explore the full extent of allelic heterogeneity and perform fine-mapping of the different causal variants across all tested traits.

Third, we showed that genetic regulation of gene expression is tightly linked to selective pressure. Indeed, genes whose regulation is mostly tissue specific tend to be more intolerant to loss of function and linked to general developmental processes whose disruption may cause a significant fitness deficit to the organism. A natural explanation for this finding would be that these genes cannot generally afford genetic variation within their promoters or broadly shared enhancers because of selection pressures and consequently only tissue-specific regulation (perhaps in tissues not crucial to the primary gene’s function) is observable via common variant eQTL studies. Consequently, it is not surprising that genes with primarily tissue-specific eQTLs and variants that regulate gene expression in a tissue-specific manner are more enriched in Mendelian and complex human traits, as our analyses revealed.

Last, we demonstrated that CAFEH can be employed to predict the target tissue in individual genomic GWAS loci. Although researchers have increasingly relied on colocalization to identify gene candidates for significant GWAS associations,^46^ previous attempts to define the target tissue on the basis of eQTL data have shown some promise^42^ but the use of colocalization for the purpose of establishing the tissue of interest in GWAS loci is still a matter of debate. Several investigators have shied away from recommending the use of eQTL information to prioritize target tissues in GWAS,^42^^,^^50^ citing the tissue-sharing of *cis*-eQTLs in a large fraction of trait associations as one reason.^51^ Recent work by us^52^ and others^4^^,^^53^^,^^54^ has demonstrated the existence of tissue-specific eQTLs and shown potential for leveraging those eQTLs to understand broad patterns of tissue enrichment for human complex traits. For example, Majumdar et al. showed that tissue-specific eQTLs can be employed to generate tissue polygenic risk scores for complex traits.^54^ Similarly, other groups have shown enrichments of complex traits for biologically relevant tissues by using colocalization or mediation approaches on eQTL data.^4^^,^^53^^,^^55^ However, as a result of the inability of existing methods to fully evaluate allelic heterogeneity and LD, the extent of tissue specificity of eQTLs has not been previously fully explored or harnessed. Specifically, it remains an open question whether the observed tissue-specific enrichments are driven by a small number of genes known to possess eQTLs only in specific tissues or by pervasive patterns of tissue specificity confounded by allelic heterogeneity and LD. Our study of eQTLs via CAFEH strongly suggests the latter and opens the door to using the allelic heterogeneity of eQTLs and GWAS to generate mechanistic hypotheses of variant and tissue targets for GWAS loci broadly. Indeed, our findings suggest that when a colocalizing eQTL signal is present within the GWAS locus, CAFEH can leverage its tissue-specific regulation to improve accuracy in identifying target tissues in which said component exerts its effect.

Naturally, we don’t expect that CAFEH will be able to pinpoint the target genes and tissues in all GWAS loci. Recent estimates from Yao et al.^4^ suggest that on average only 11% of complex trait heritability is mediated via *cis*-eQTLs in bulk tissues via data from GTEx v7 and eQTLGen. Although those estimates are derived from broad heritability signals, including small subthreshold effects that may not necessarily reflect the behavior of the strongest non-coding GWAS loci, it is important to highlight situations where a *cis*-eQTL colocalization approach would be expected to fail to show evidence for colocalization. First, loci whose effects are mediated via coding variants may not demonstrate eQTL effects and therefore cannot be explored with CAFEH. Given the fact that non-synonymous coding variation is under strong selection pressure, this phenomenon is most likely rare in GWA studies that focus on common variant analysis.^56^ Second, certain complex trait risk variants could influence splicing or could act via effects on expression of distant genes. CAFEH can still be employed in those cases if there is availability of splice QTL or distant eQTL data for different tissues. Most importantly though, the observed pervasive tissue specificity of eQTLs underscores the limitation of using bulk eQTL data for the purpose of GWAS locus exploration and provides a potential explanation for the underwhelming estimates of trait heritability mediated via *cis*-eQTLs. Indeed, in the presence of pervasive allelic heterogeneity across tissues, bulk tissue eQTLs are unlikely to be a good surrogate for cell-type-specific signals, especially for cell types that are not dominant in the tested tissues. In addition, it is likely that context-specific effects, such as infection response^57^ or development, could also mediate the effect of certain common variants on complex traits. Therefore, as we have shown here and previous studies support,^57^^,^^58^ a broader set of eQTL data from specific cell types and different contexts is likely to improve our ability to identify the cell type of interest across different GWAS traits. Lastly, we should note that colocalization does not necessarily suggest mediation and it remains possible that colocalizing signals can be observed without a linking causal pathway due to horizontal pleiotropic effects.

Our study has several limitations. First, the current version of CAFEH relies on a single LD matrix, therefore large differences in LD between studies, due to ancestry differences or other factors, could impact inference of colocalization and fine-mapping. Second, CAFEH includes a simple prior on variant causality, assuming equal prior probability across all evaluated variants for a locus. While this performs well in our analysis, CAFEH could be extended to incorporate informed priors based on variant annotations such as regulatory element annotations or conservation scores. Third, in its current form, CAFEH does not handle missing data in the effect size matrix, which implicitly assumes that the causal variants are present in the tested datasets. A potential solution to this problem would be to first impute missing variants via a reference LD matrix and use the imputed variants as input to CAFEH.^53^ Last, although CAFEH represents a significant advance in evaluating allelic heterogeneity compared to prior methods, it is still difficult distinguish the presence of multiple signals among variants in nearly perfect or very high LD with each other. In that case, one can expect CAFEH to assign all variants in a high LD block almost equal posterior inclusion probability in a single causal component. More extensive evaluation of those cases would require either an extension of our method to incorporate distinct LD structures from different populations (if those are available) or experimental validation.

Our analysis has broad implications for the interpretation of disease-associated loci and the overlap with eQTLs from diverse tissues and contexts. Previous work would suggest that there are a large number of common variants in the human genome with ubiquitous effects across tissues but that are not the primary contributors to disease risk given the limited causal overlap observed. Our refined analysis instead suggests much greater levels of tissue-specific genetic effects than previously appreciated and a greater ability to colocalize disease loci with genetic variants in the correct tissue. However, we are still far from characterizing every disease locus, and the patterns of disease overlap we observe indeed indicate that tissue-specific eQTLs are more likely to underlie disease risk. Together, the presence of profound tissue and cell-type specificity of gene expression regulation in our study and the observed patterns of colocalization hint that bulk tissue eQTLs in adult tissue may not be sufficient to explain complex trait association signals, thereby underscoring the need for more widespread cell-type- and context-specific eQTL studies.

In summary, our results provide evidence of pervasive tissue specificity in genetic regulation of gene expression. We develop a computational tool, CAFEH, to perform fine-mapping and colocalization jointly across multiple tissues and traits that allows multiple causal variants present across studies and can better explore sharing in the presence of allelic heterogeneity. We use that tool to show that genes whose transcriptional regulation is tissue specific, despite being broadly expressed and genetically regulated, tend to be under greater selective pressure and more relevant in disease and that causal GWAS signals are more likely to be tissue specific than shared. Finally, we demonstrate that the method is effective at leveraging the tissue specificity of eQTLs to improve the identification of target tissue in GWAS loci.

## Data and code availability

CAFEH is available in GitHub: https://github.com/karltayeb/cafeh. All GTEx v8 data are available through dbGaP (dbGaP: phs000424.v8).

## Supplemental information

Document S1. Figures S1–S33 and supplemental methods

Table S1. GO enrichment analysis of highly and poorly colocalizing genes according to CAFEH

Document S2. Article plus supplemental information
