# Supplementary material for: Redefining tissue specificity of genetic regulation of gene expression in the presence of allelic heterogeneity
Source: Am J Hum Genet. 2022 Jan 31;109(2):223–39. doi: 10.1016/j.ajhg.2022.01.002 (PMC8874223; doi:10.1016/j.ajhg.2022.01.002)
Supplement: Document S1. Figures S1–S33 and supplemental methods [file mmc1.pdf]

The American Journal of Human Genetics, Volume *109*

Supplemental information

Redefining tissue specificity of genetic regulation of gene expression in the presence of allelic heterogeneity

Marios Arvanitis, Karl Tayeb, Benjamin J. Strober, and Alexis Battle

Supplemental Figures and Legends

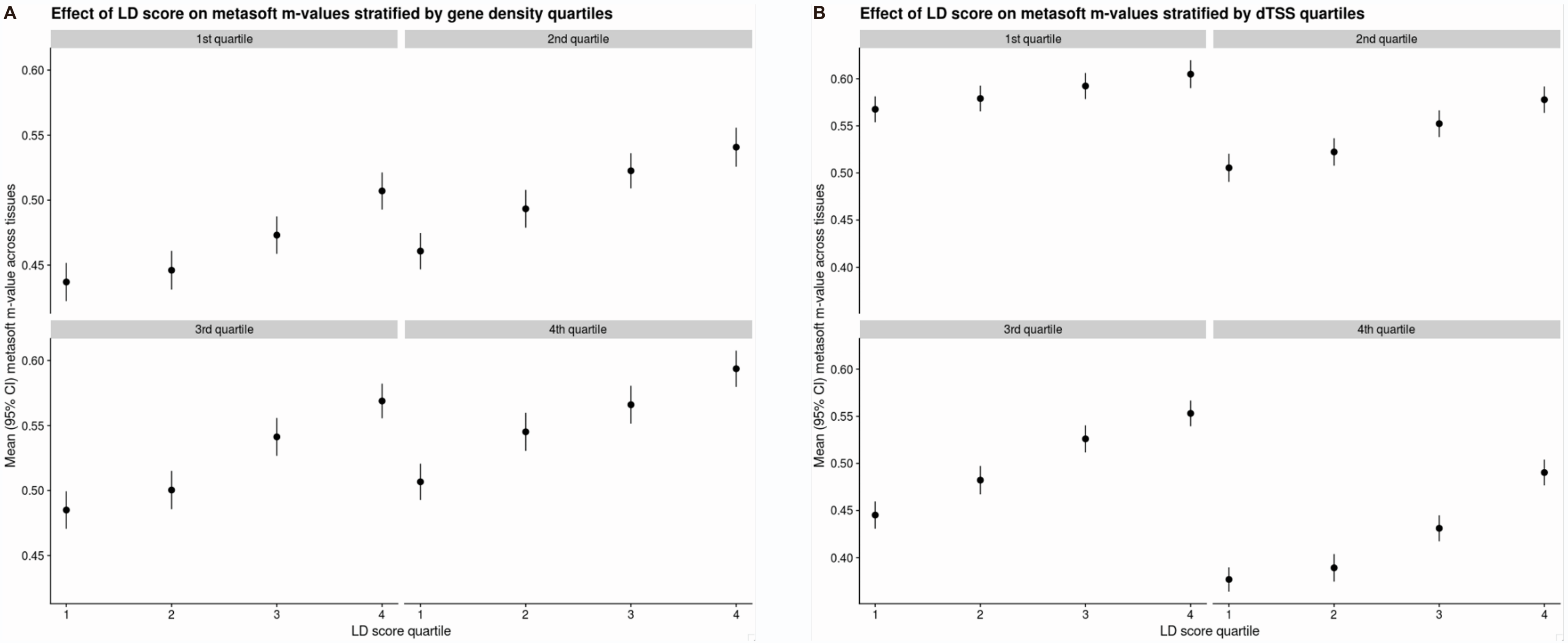

Figure S1: Association between metasoft m-value and LD structure. Average m-value across tissues stratified by the LD score quantile of the tested variant in quartiles of **A.** gene density (number of genes within 1Mb of the tested variant) or **B.** distance to nearest transcription start site (TSS).

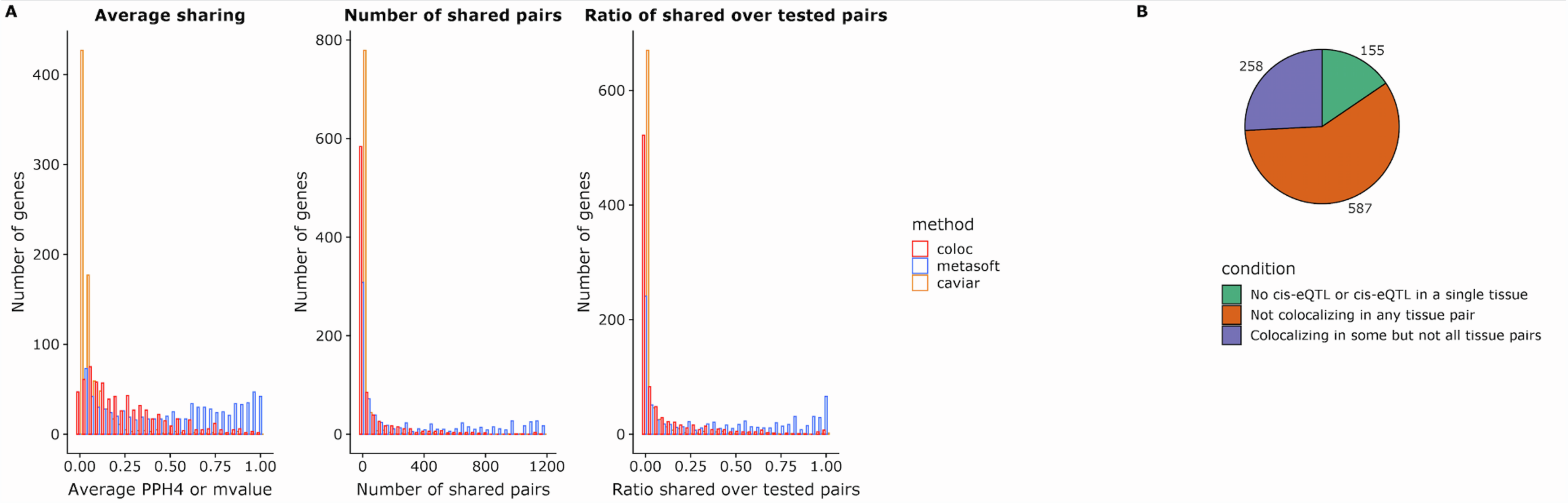

Figure S2: Comparison of cis-eQTL tissue sharing between COLOC, eCAVIAR and Metasoft in GTEx v8. A. Histograms depicting patterns of sharing of genetic regulation between tissues based on eCAVIAR, COLOC and Metasoft in a randomly sampled subset of 1000 genes among all 38,518 genes expressed in at least one tissue in GTEx v8. eCAVIAR and COLOC reveal substantial tissue specificity. **B.** Pie-chart of the same randomly sampled subset of 1000 genes in GTEx v8 based on the tissue specificity of their eQTLs estimated by eCAVIAR assuming ≤2 causal variants.

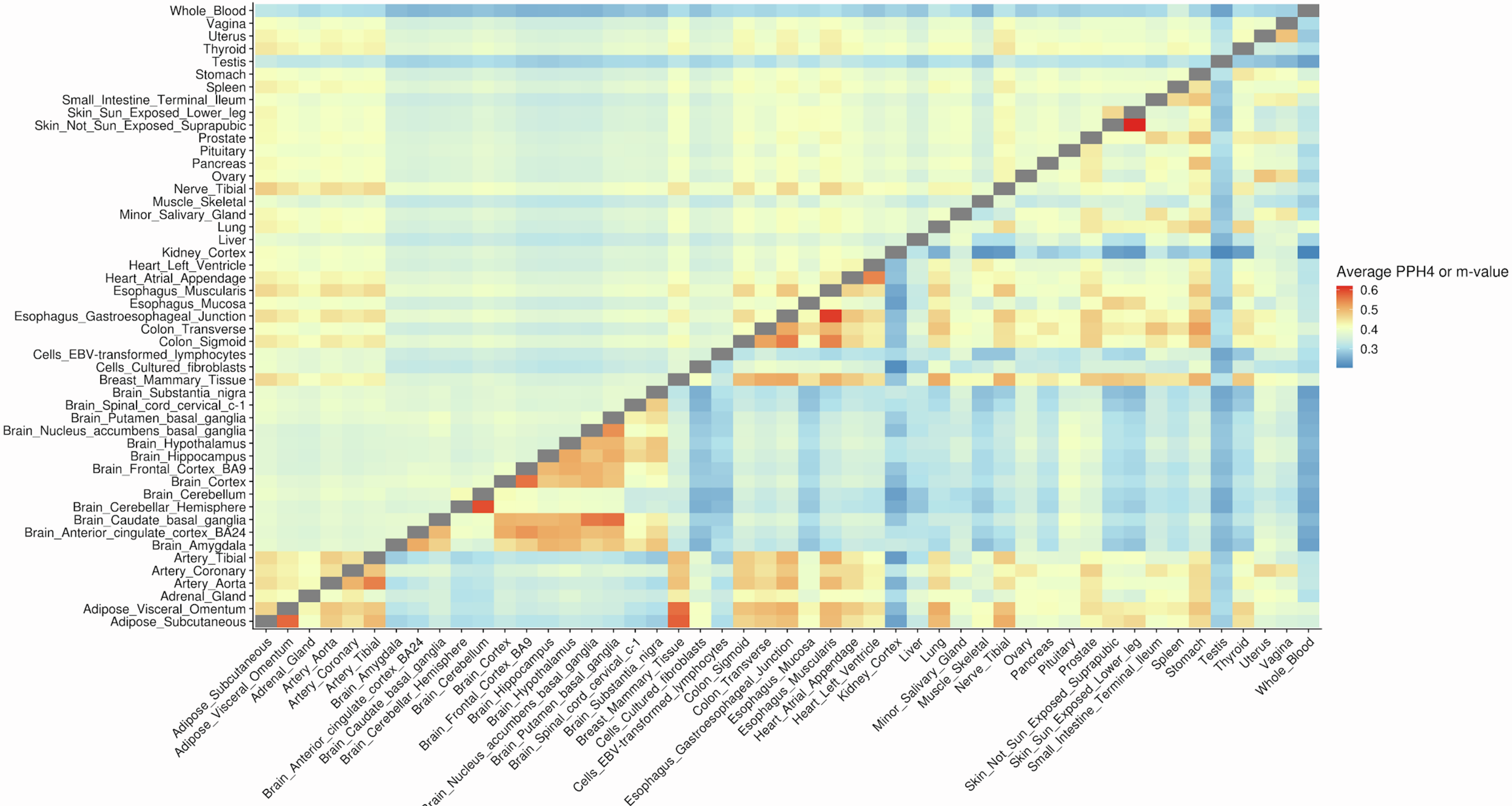

Figure S3: Heatmap of the average COLOC PPH4 or Metasoft m-value between pairs of tissues. Unlike the m-value, PPH4 reveals known biological patterns of tissue similarity.

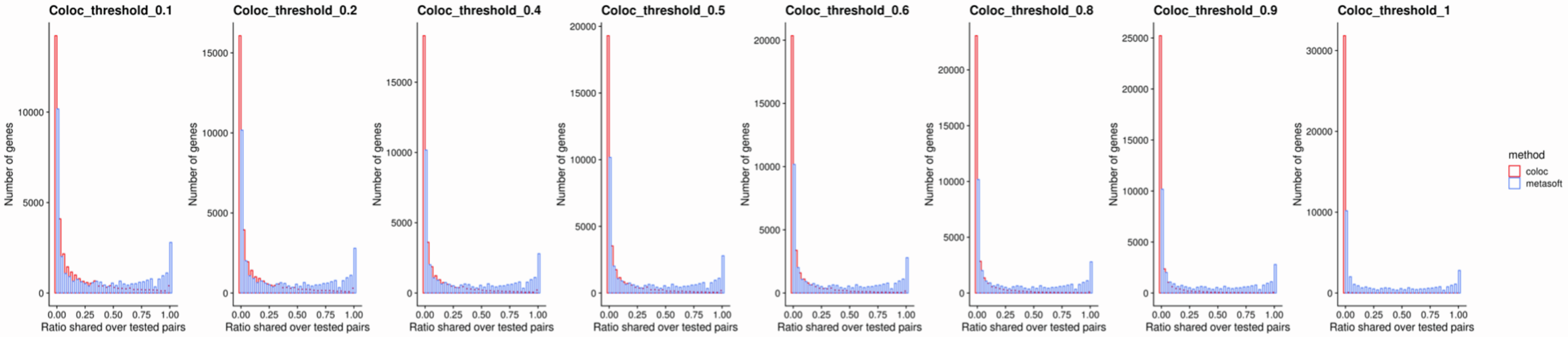

Figure S4: Comparison of eQTL tissue sharing between COLOC and Metasoft at different PPH4 thresholds. Histograms depicting ratios of shared tissue pairs among all tissue pairs in which each gene is expressed for different thresholds of COLOC PPH4 and for metasoft m-value threshold of 0.5 in all tissues of GTEx v8.

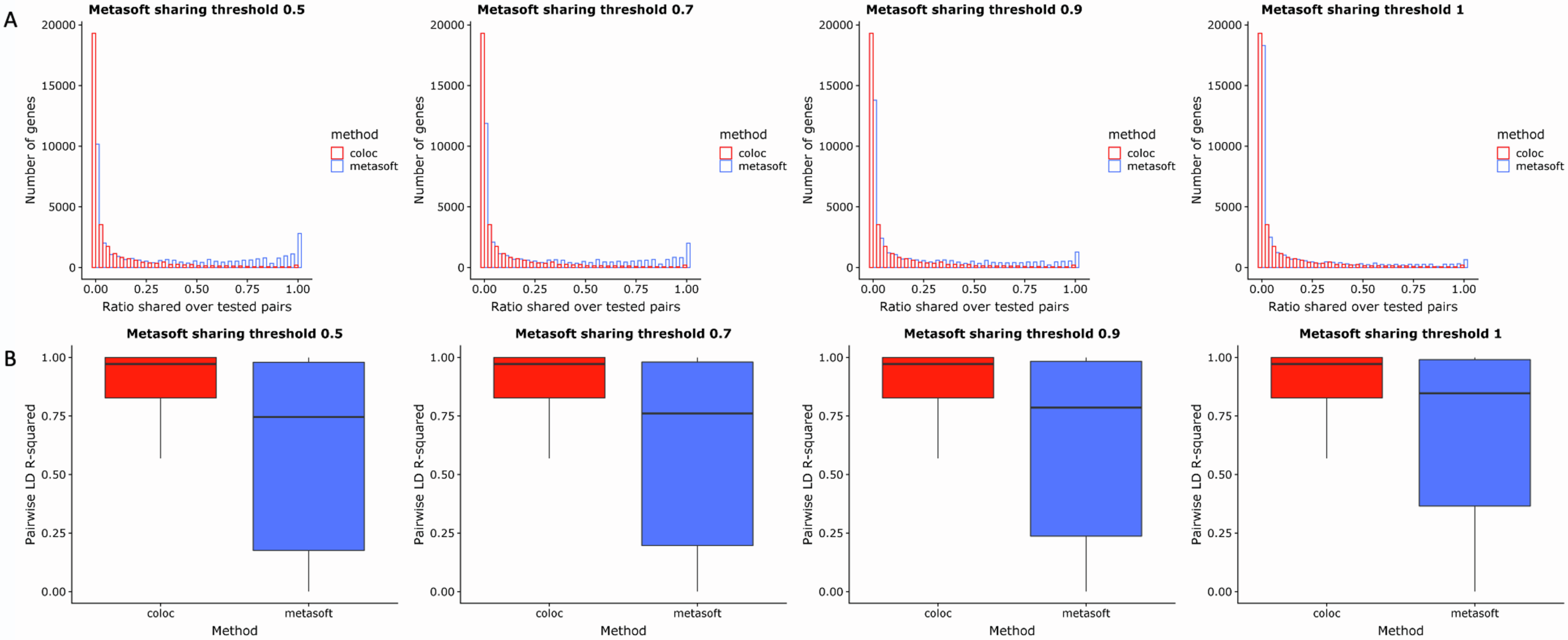

Figure S5: Comparison of eQTL tissue sharing between COLOC and Metasoft at different m-value thresholds. A. Histograms depicting ratios of shared tissue pairs among all tissue pairs in which each gene is expressed for different thresholds of metasoft m-value and for COLOC threshold of 0.5 in all tissues of GTEx v8. **B.** Boxplots of the LD between the top variants in tissue pairs that colocalize based on COLOC (in red) or Metasoft (in blue) at different metasoft thresholds.

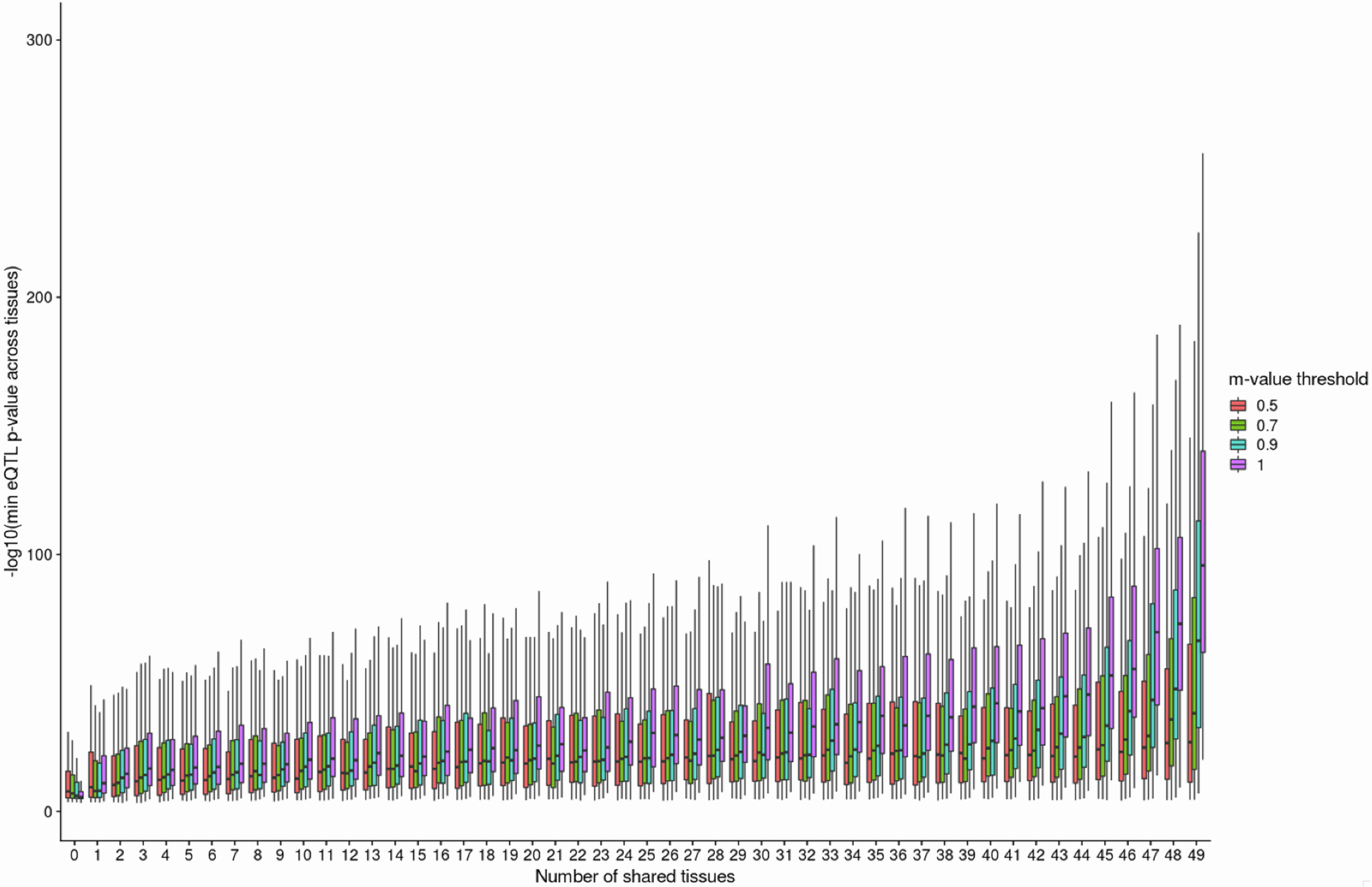

Figure S6: Correlation between strength of eQTL association and degree of tissue sharing by Metasoft. We see that at higher m-value thresholds, Metasoft preferentially identifies eQTLs with a strongest association (denoted by the minimum eQTL p-value across tissues for that gene) as shared.

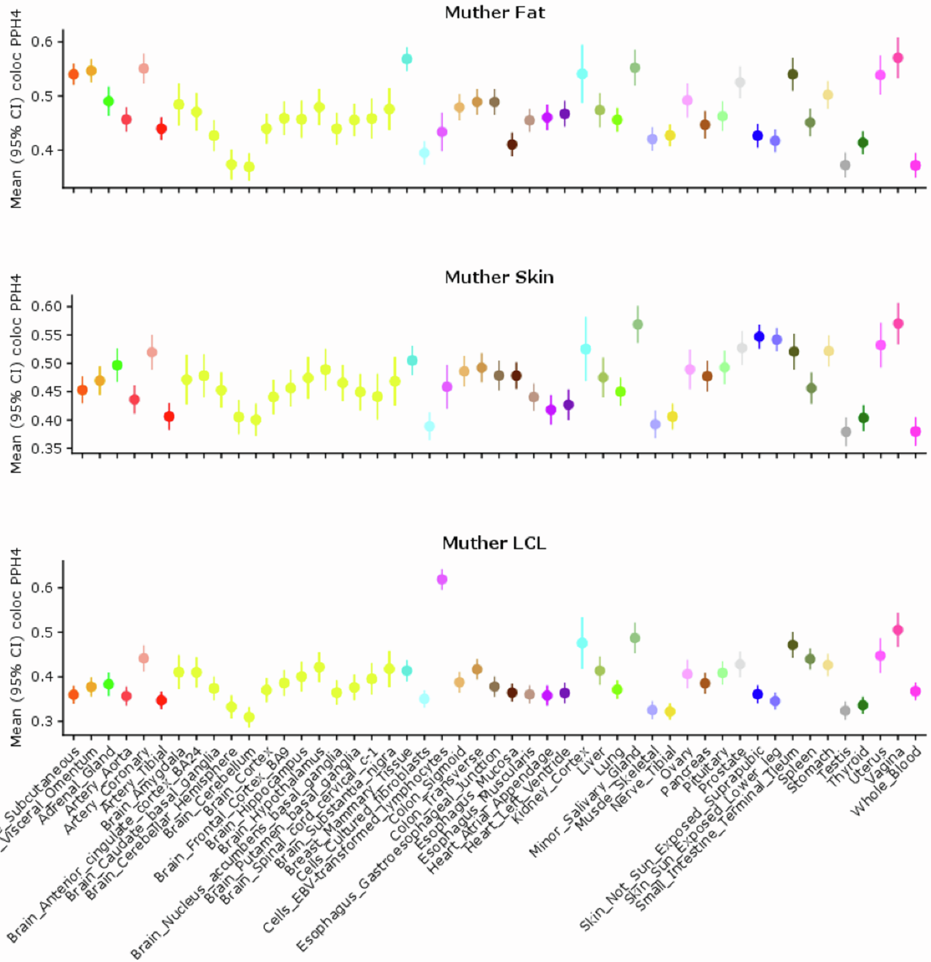

Figure S7: Average eQTL sharing between three tissues(Fat, Skin and LCL) in Muther and all GTEx v8 tissues for genes that have an eQTL in both datasets.

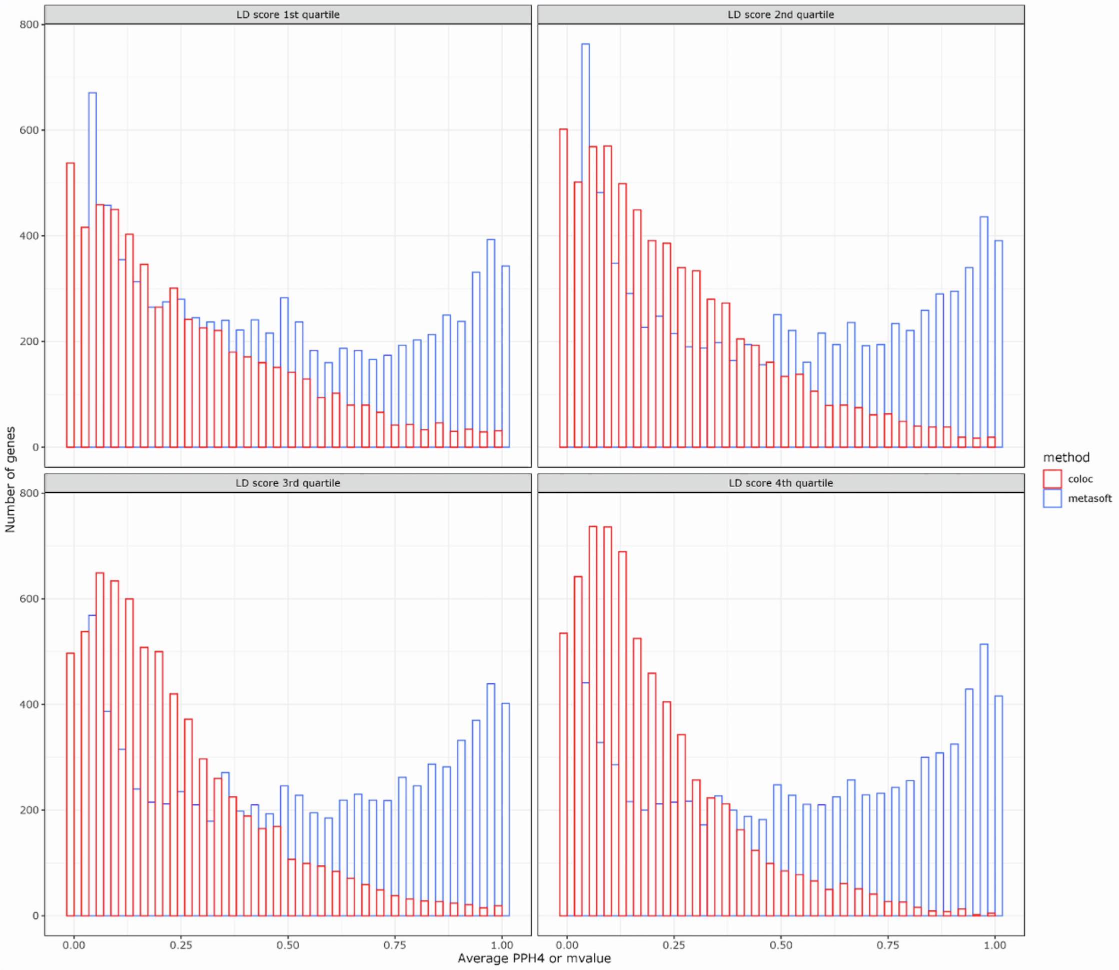

Figure S8: Histograms of tissue sharing by COLOC and Metasoft in quartiles of LD for the top eQTL variant in the tested pair. COLOC reveals more tissue specificity in all quartiles.

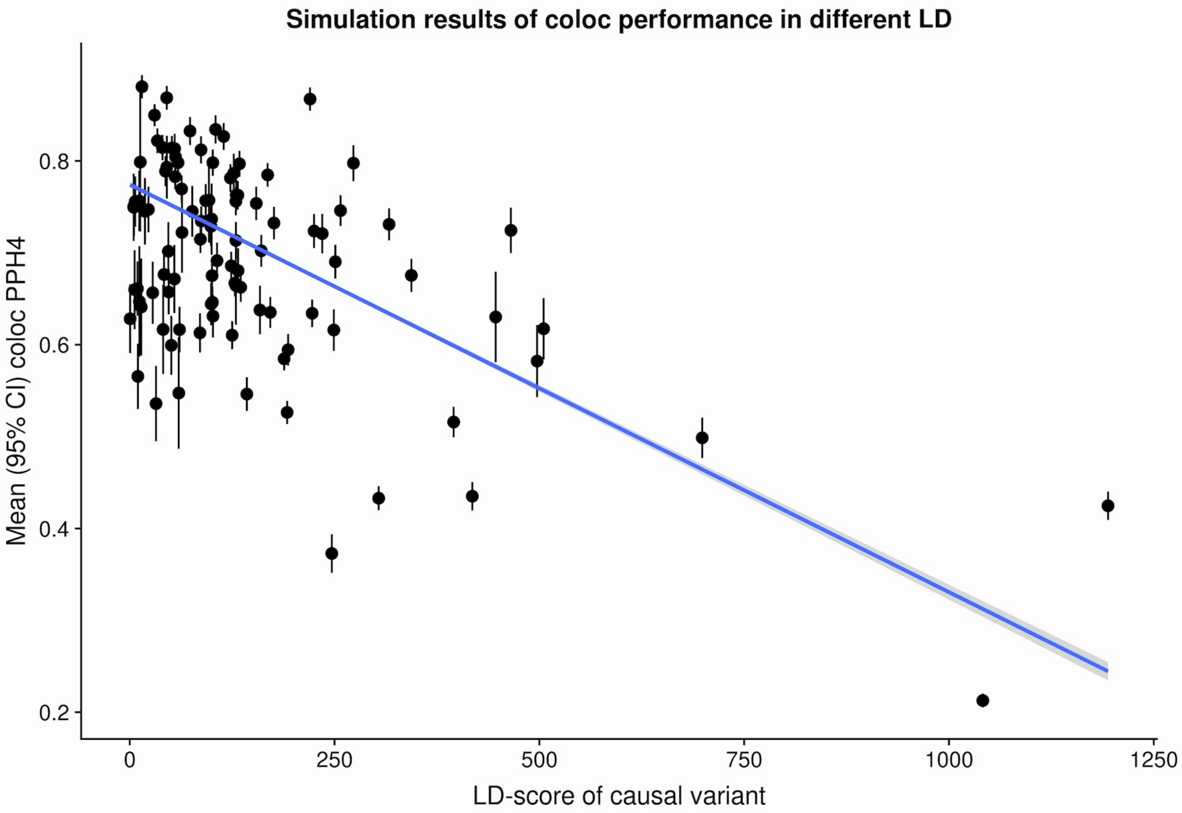

Figure S9. Simulations of coloc PPH4 when causal variants are shared between studies stratified by LD. Average PPH4 between studies that share the same causal variants are stratified by the LD score of the causal variant in simulations. Each dot represents the mean across all simulations for that variant. Standard errors between simulations are also plotted, as is the fitted regression line.

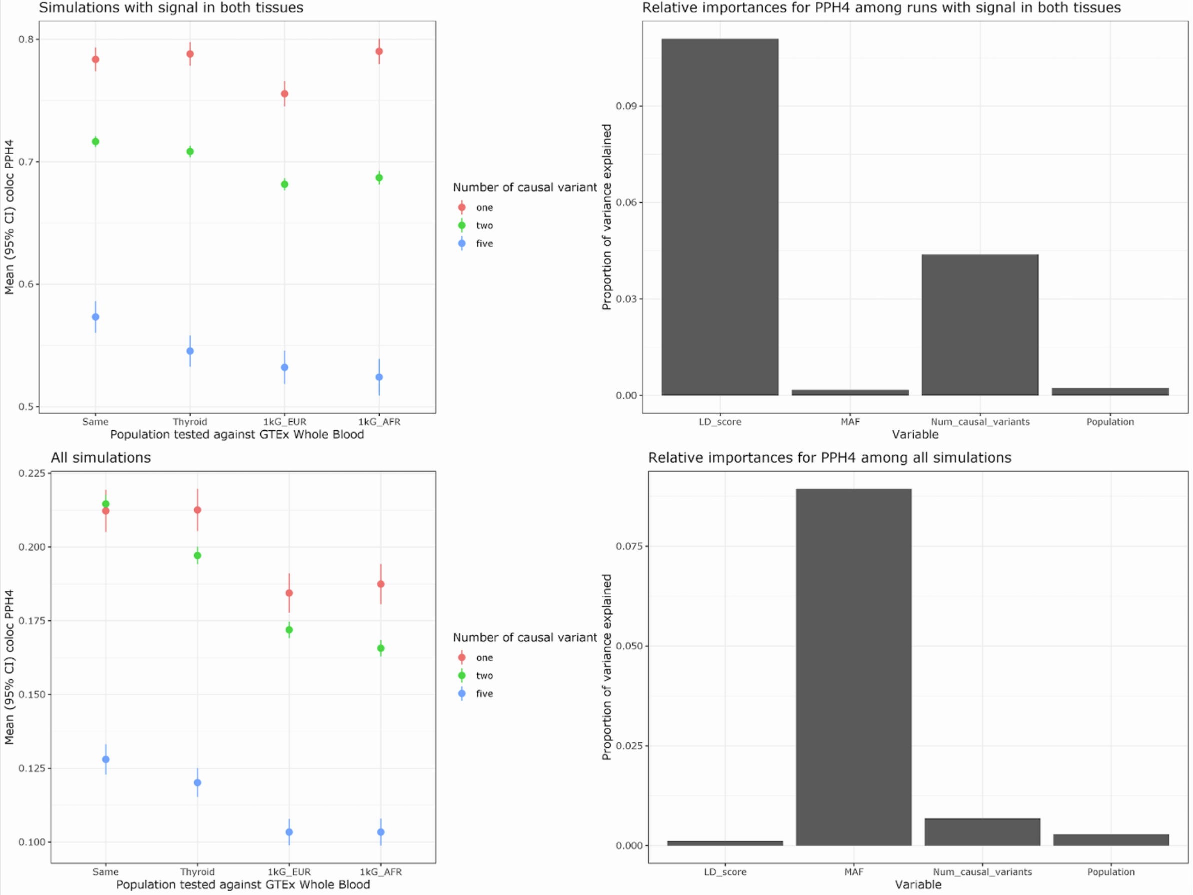

Figure S10: Simulations assessing COLOC performance in colocalizing studies. Left panel shows posterior probability of colocalization in all simulations (bottom) or in simulations that have an active signal in both tissues (top) across different populations and numbers of causal variants. Right panel shows relative importances of four parameters (LD score of the causal variant, Minor allele frequency of the causal variant, number of causal variants and population compared with GTEx) in determining the value of PPH4.

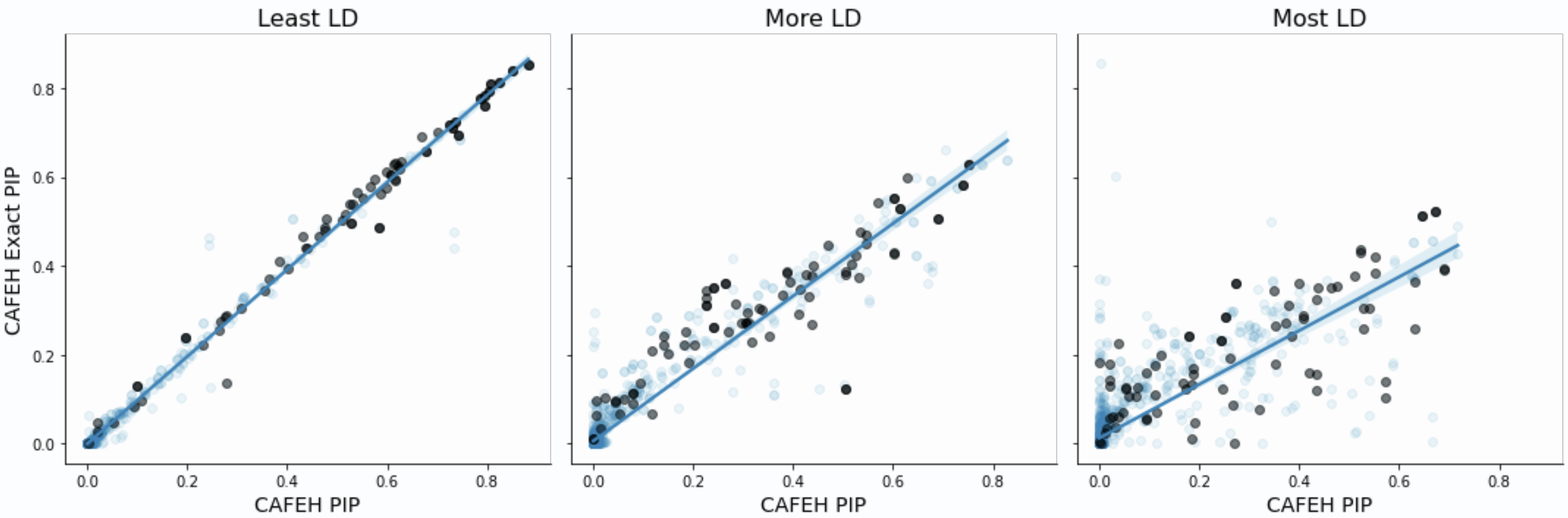

Figure S11: Variational inference vs exact inference in CAFEH: We simulate two traits with three causal variants where one causal variant is shared, and one causal variant is distinct to each trait. For each simulation we generate 50 “variants” from a multivariate normal distribution, with covariance set to reflect varying degrees of LD. For each level of LD we replicate the simulation 20 times. CAFEH (K=3) is fit using the variational approximation, or exact inference. Plots show posterior inclusion probabilities (PIPs) CAFEH’s variational approximation against the exact computation for the low, medium, and high LD simulations (left, center, right, resp). Causal variants are indicated in black.

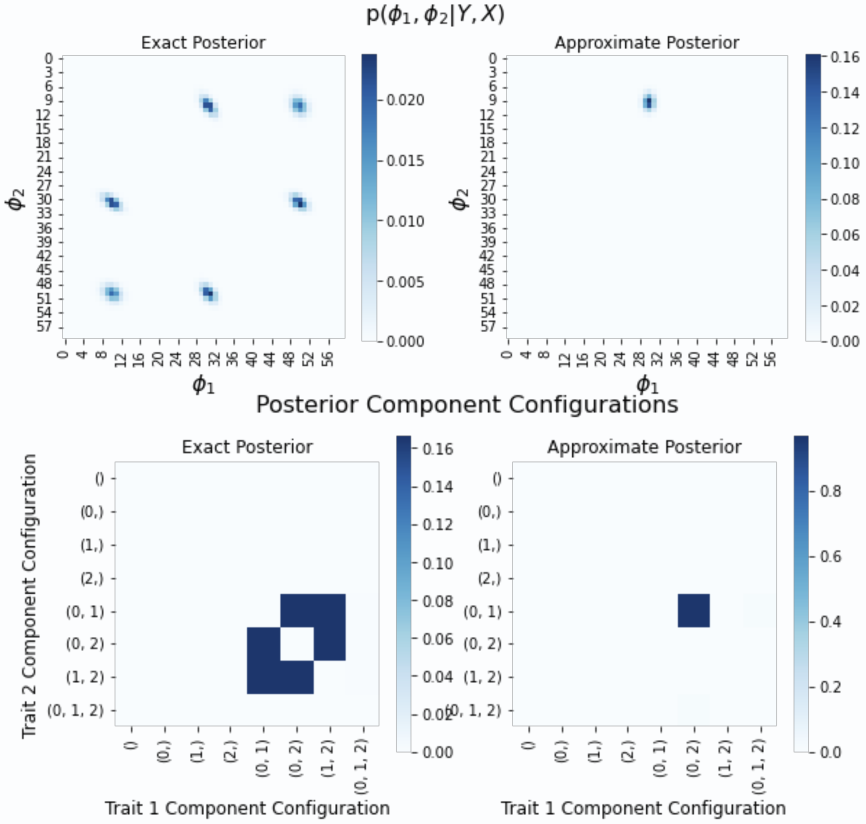

Figure S12: CAFEH's variational approximation identifies modes of the exact posterior. We simulate two traits with three causal variants where one causal variant is shared, and one causal variant is distinct to each trait. We plot the joint posterior distribution of two components (top) and component configurations for both traits (bottom) for the exact (left) and approximate (right) inference schemes. CAFEH's approximate posterior identifies one of several equivalent modes in the true posterior.

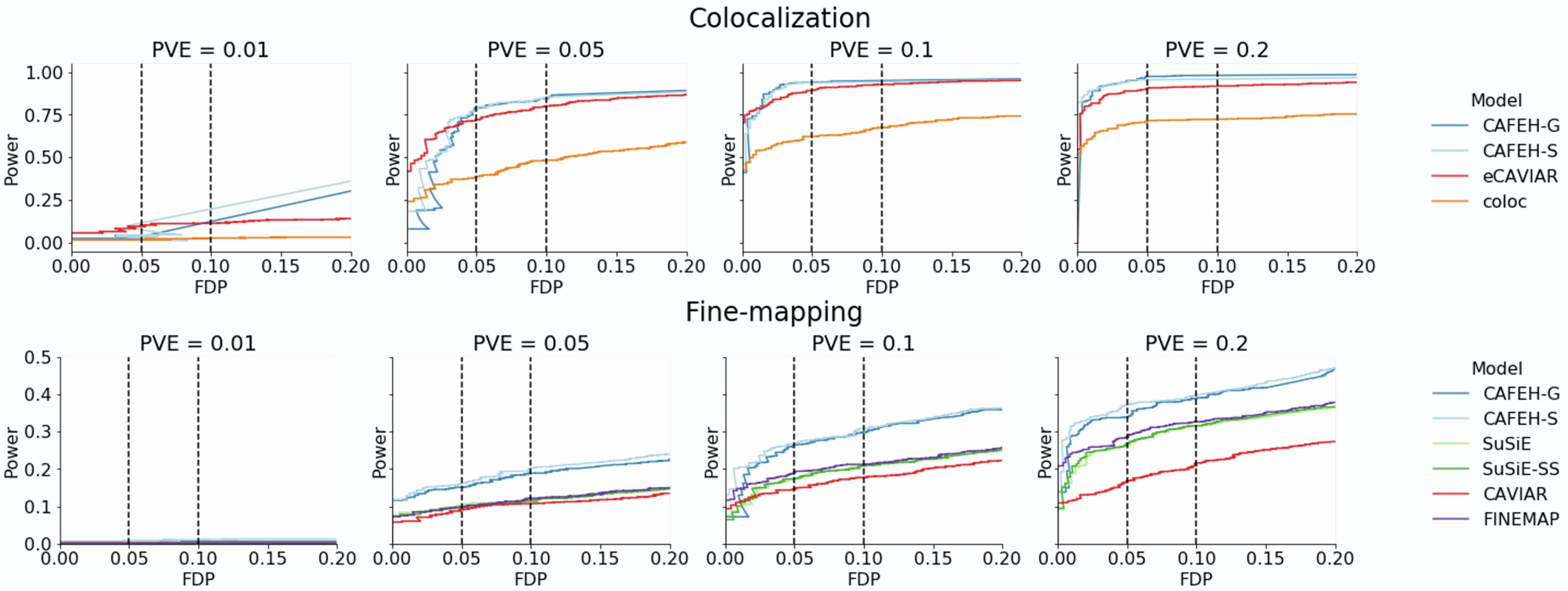

Figure S13: Comparison of colocalization and fine-mapping performance of various methods at varying signal strength. A. We compute power and false discovery proportion at varying thresholds of the colocalization statistics of each method (PPH4 for coloc, CLPP for eCAVIAR, p_coloc_any for CAFEH). **B.** We compute power and false discovery proportion at varying thresholds of the posterior inclusion probability for each method.

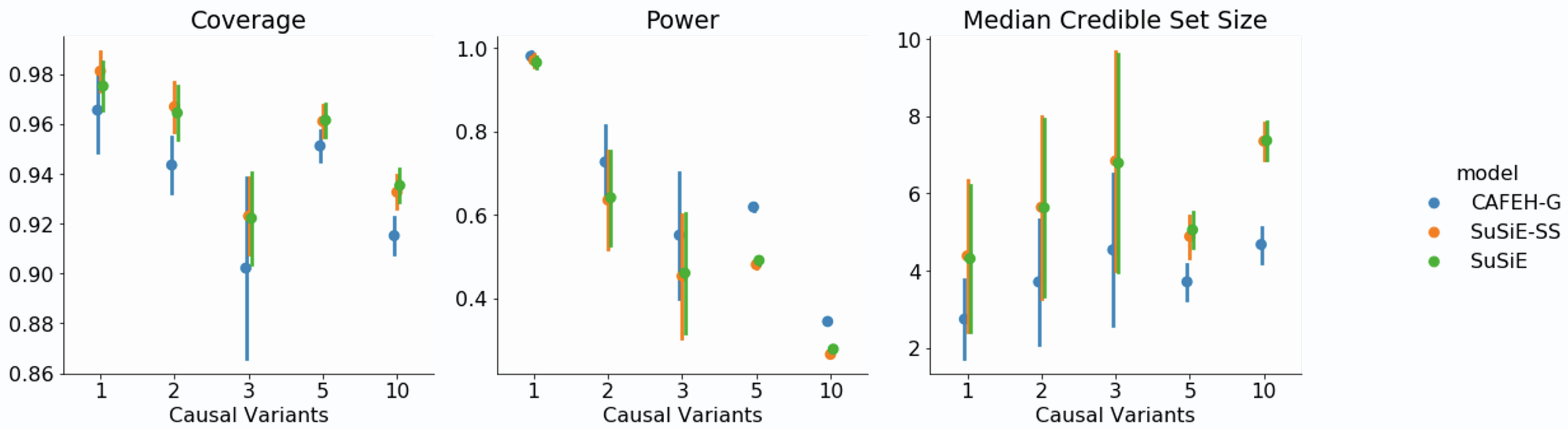

Figure S14: Comparison of 95% credible sets for CAFEH-G, SuSiE-SS, and SuSiE. A. coverage, proportion of 95% credible sets containing a causal SNP. **B.** Power, proportion of all causal SNPs detected in a credible set. **C.** Median credible set size. Confidence intervals computed from 100 bootstrap iterations. Simulations with 1-3 causal variants performed on 1000 SNPs, simulations with 5 and 10 causal variants performed on all SNPs in 1Mb region of gene transcription start site.

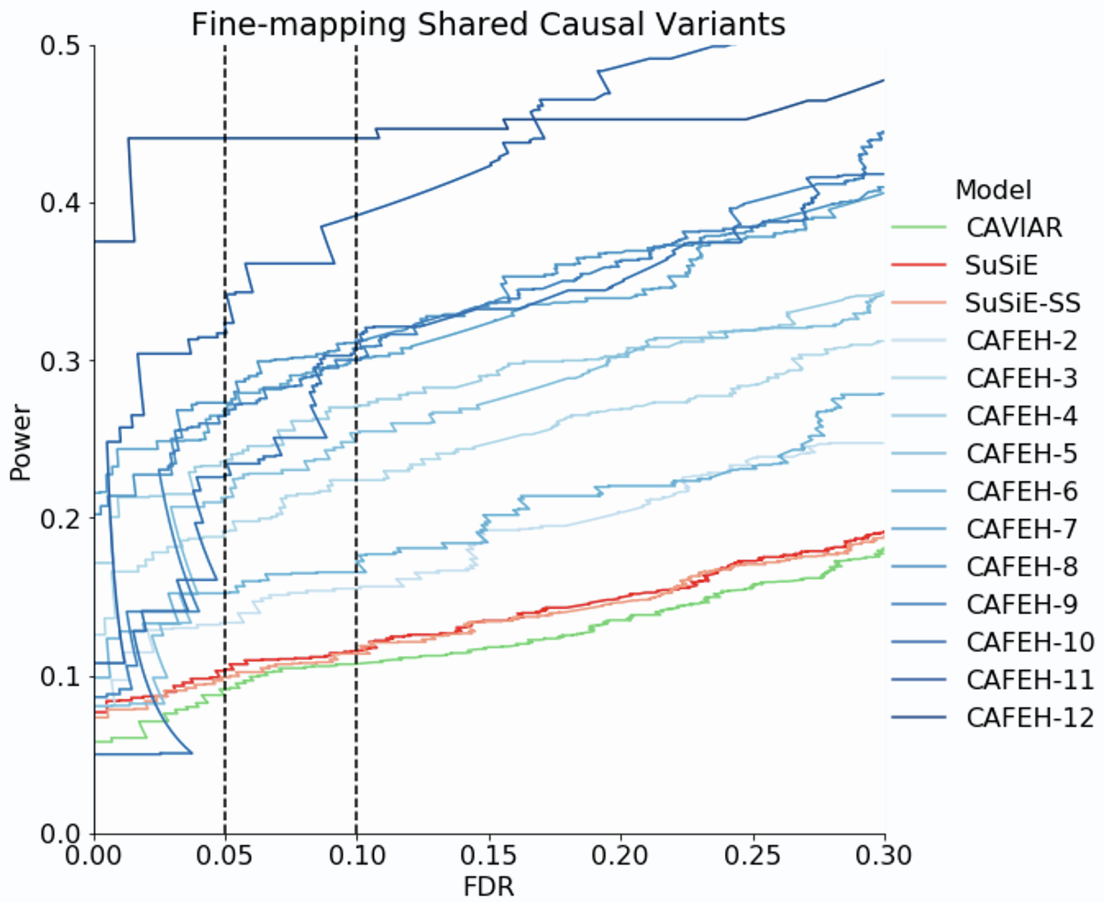

Figure S15: Improved fine-mapping of shared causal variants. We conduct a range of simulations where the causal variant is shared between 1-12 tissues. We vary the threshold of posterior inclusion probability (PIP) for each method and compute the proportion of false discoveries (FDP) and the proportion of causal variants detected (Power).

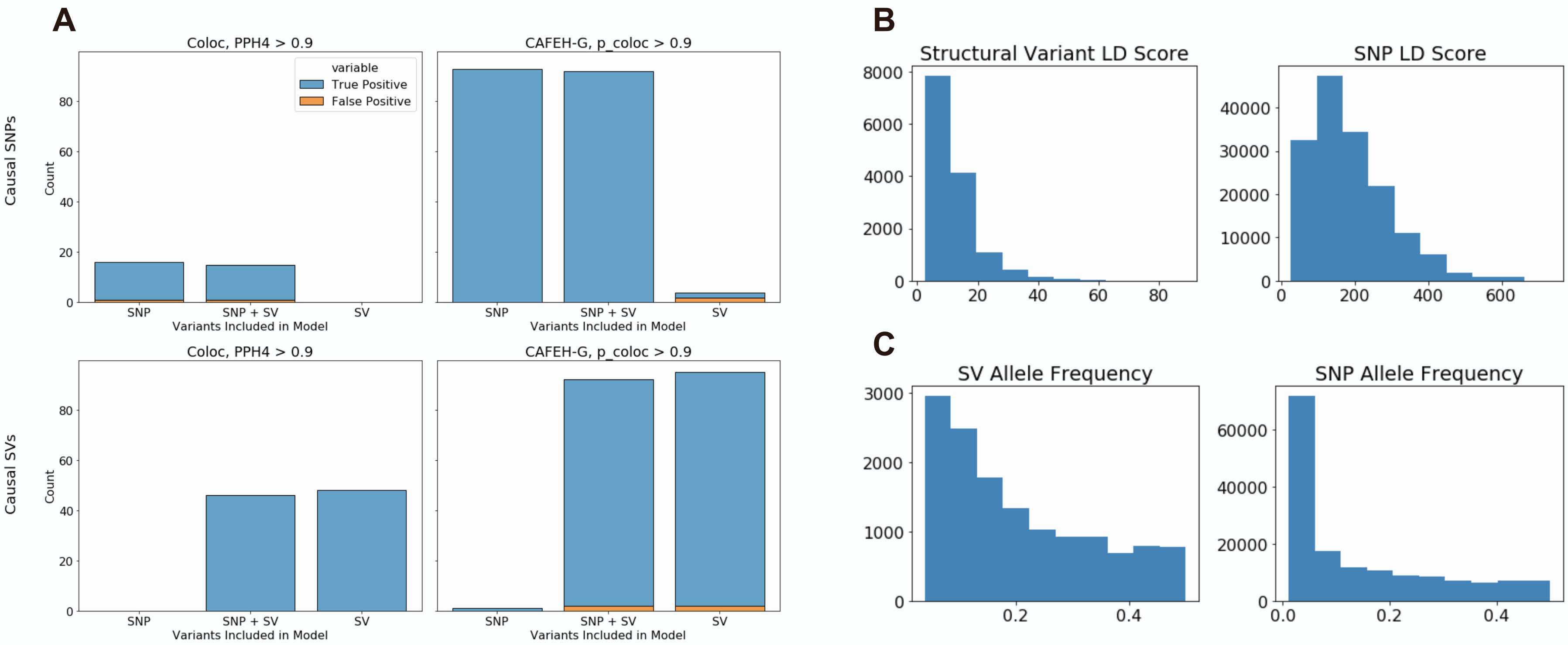

Figure S16: Structural variant simulations. We consider applicability of CAFEH to the colocalization of structural variants (SVs). Simulations are generated where the causal variant(s) are either SNPs (top) or SVs (bottom), and run CAFEH and coloc using only SNPs, SVs, or SNPs + SVs. Causal variants are sampled among SNPs or SVs with allele frequency > 0.05 **A.** Stacked bars count the number of true positives and false positives for coloc at a threshold of PPH4 > 0.9 (left) and CAFEH at a threshold of p_coloc > 0.9. **B.** LD scores, calculated as the sum of squared correlation between a variant and all other variants, for SVs (left) and SNPs (right) used in simulations. **C.** Allele frequency of unique SVs (left) and SNPs (right) used in simulations.

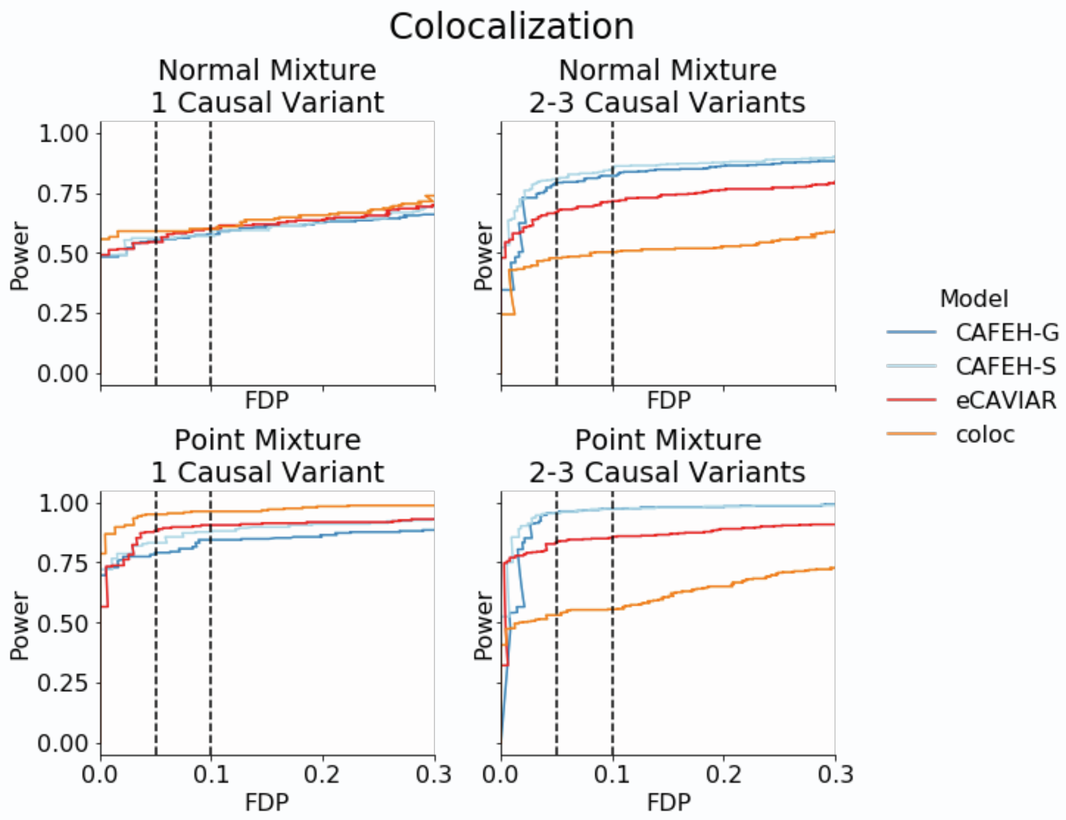

Figure S17: Colocalizing of mixture simulations: Causal variants are drawn from a mixture of 0 mean normal distributions (top) or a mixture of point masses (bottom). Plots show the trade off between power and false discovery at varying colocalization thresholds for simulations with a single causal variant (left) and multiple causal variants (right).

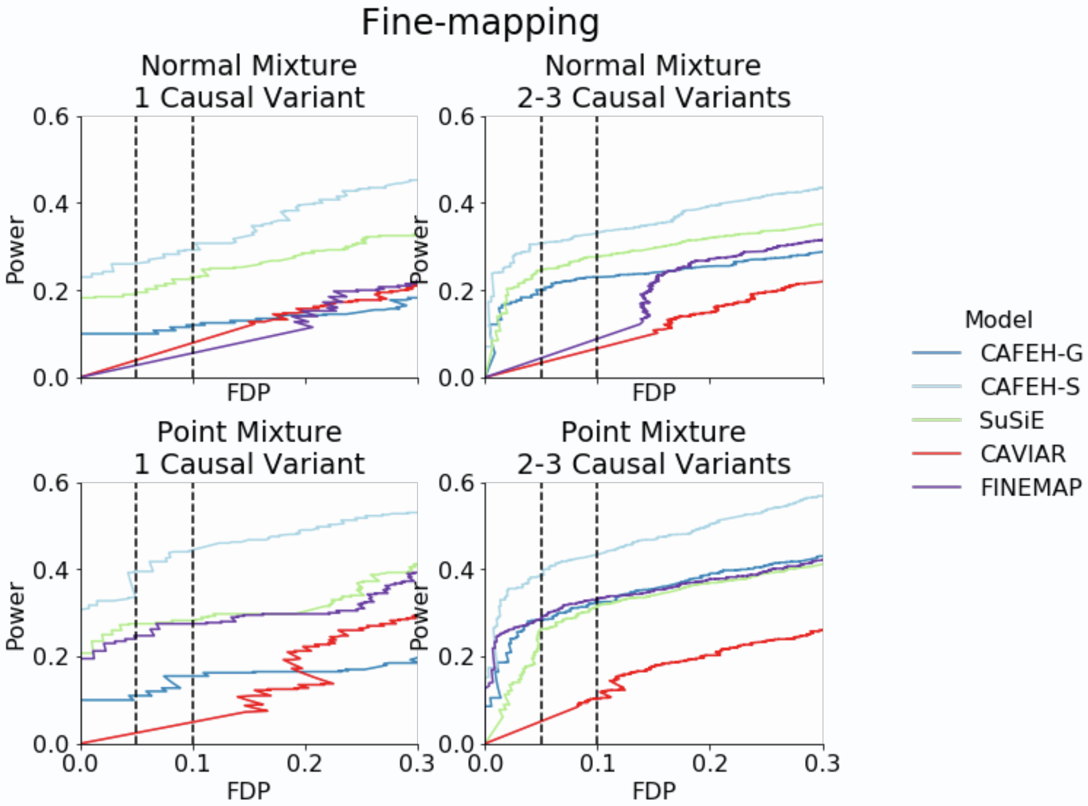

Figure S18: Fine-mapping of mixture simulations: Causal variants are drawn from a mixture of 0-mean normal distributions (top) or a mixture of point masses (bottom). Plots show the trade off between power and false discovery at varying posterior inclusion probability thresholds for simulations with a single causal variant (left) and multiple causal variants (right).

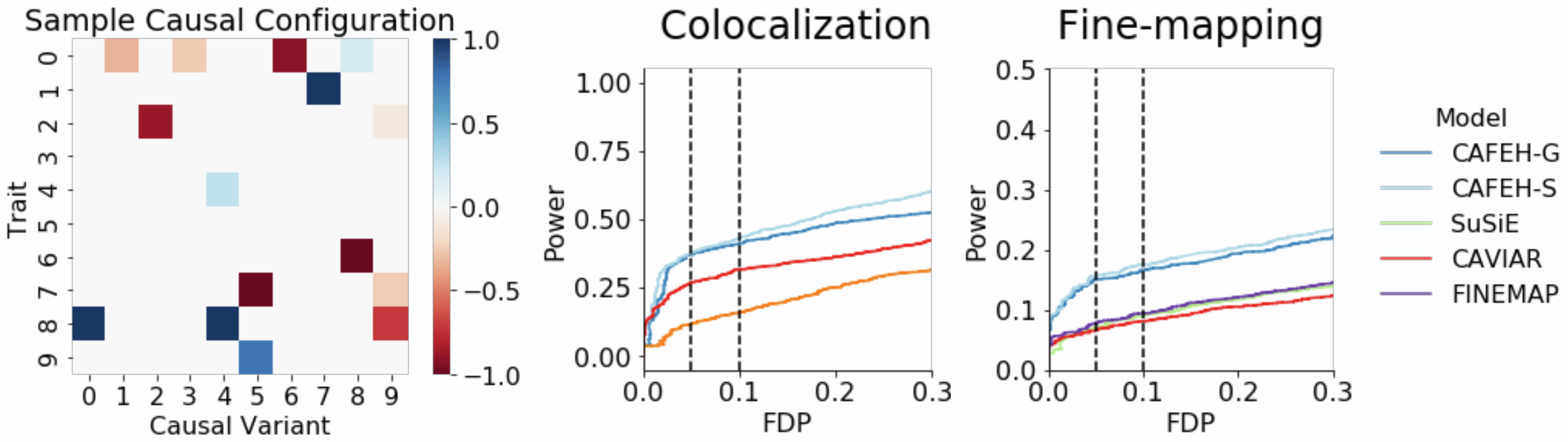

Figure S19: Fine-mapping of point-normal simulations: We simulate 10 traits with a total of 10 causal variants. Causal variants are randomly assigned to each simulated trait with probability 1/5, effects are drawn from a 0-centered Normal distribution, Normal noise is added to achieve percent variance explained 0.01, 0.05, 0.1. Panels show a sample causal configuration generated under this simulation (left) and the trade off between power and false discovery at varying colocalization thresholds (middle) and posterior inclusion probability thresholds (right) across 50 replicates of each simulation.

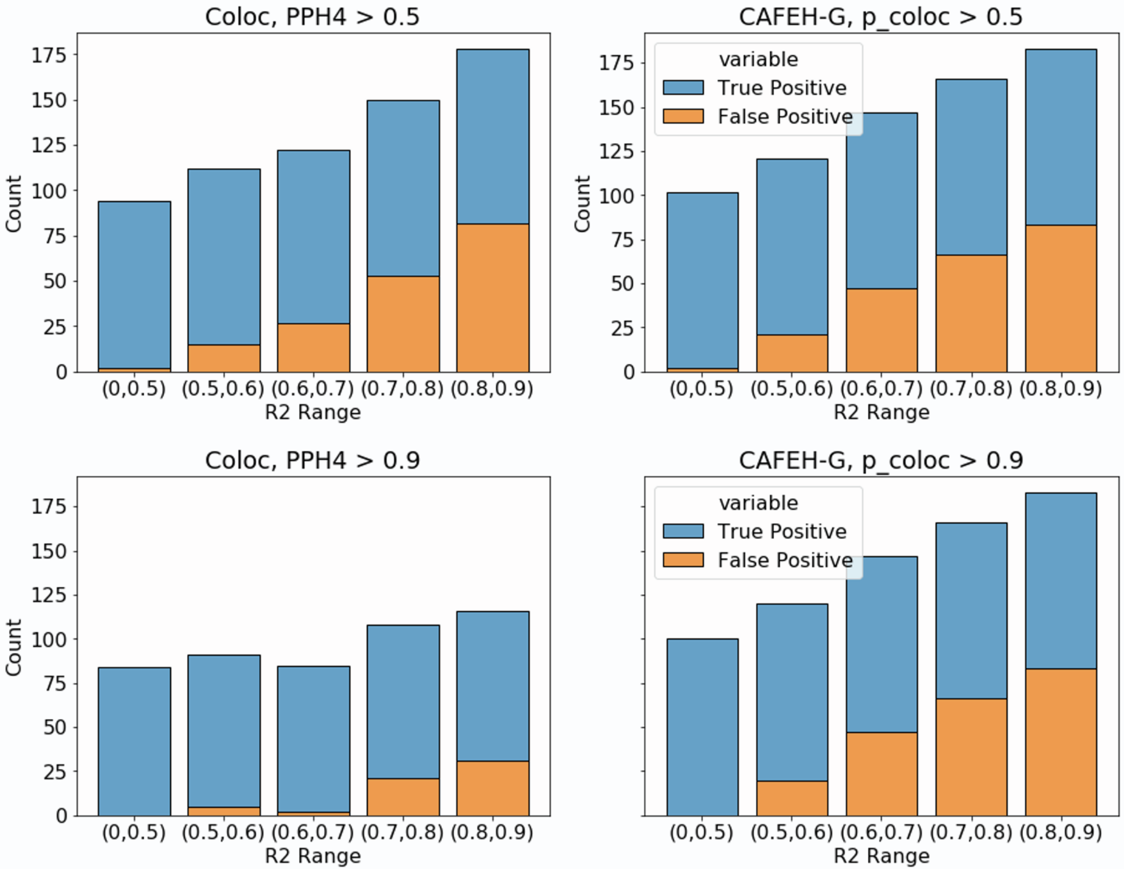

Figure S20: Simulations of CAFEH and COLOC in different ranges of LD between the causal variants. Both methods have increased numbers of false positive colocalization findings in high LD although CAFEH has more false positives when higher thresholds for colocalization are chosen and LD R2 is > 0.9.

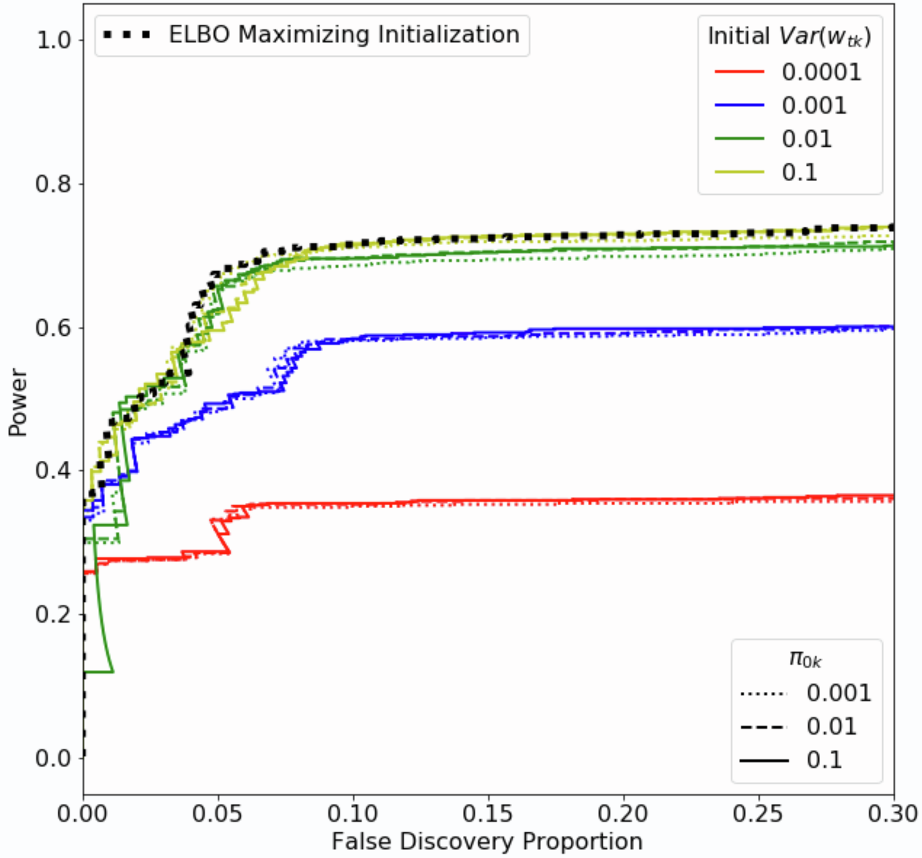

Figure S21: Sensitivity of CAFEH-G to initialization and hyperparameters. We vary the the prior spike probability and the initialization of effect size variance. Bold, black, dotted line indicates performance when selecting the model that maximized the evidence lower bound (ELBO) for each simulation. We observe that CAFEH is robust to various settings of the spike probability π_{0k}, and that our defaults ($\pi_{0k} = 0.1, Var(w_{tk}) = 0.1$) settings work well in our simulations. Among multiple initializations, choosing the ELBO maximizing initialization yields good results.

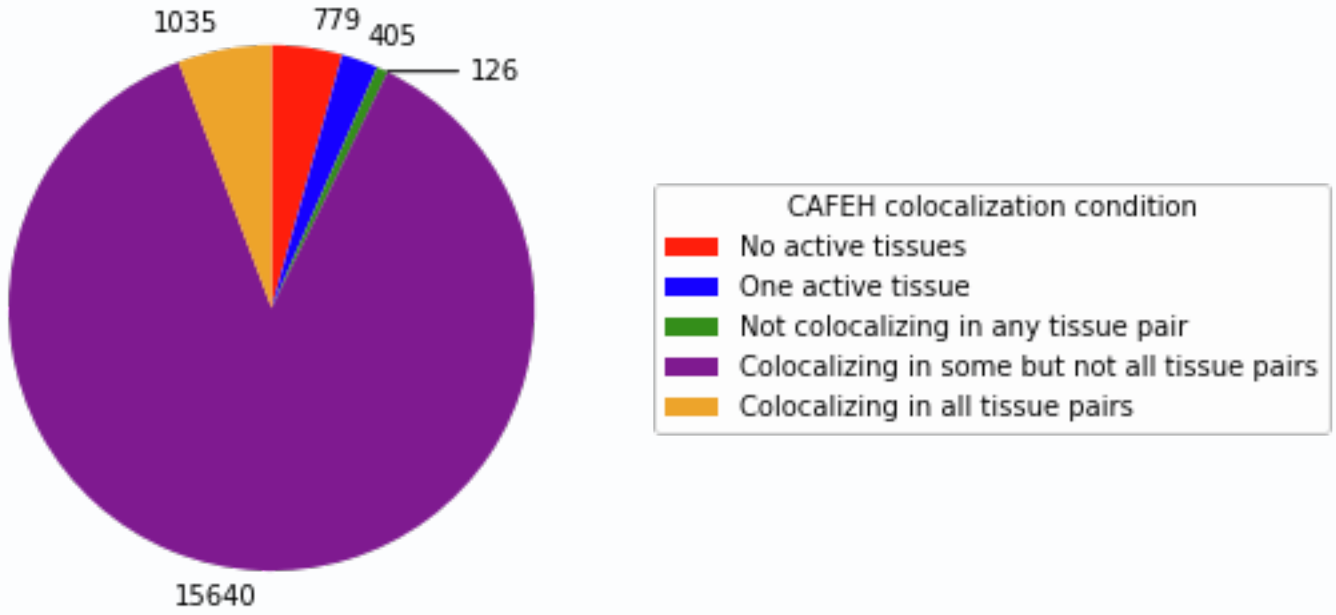

Figure S22: Protein coding genes classified by CAFEH colocalization conditions: we classify 17,985 genes expressed in at least one tissue in GTEx by the proportion of colocalizing tissue pairs in CAFEH. We consider a tissue active for a gene if it has at least one CAFEH component with p_active > 0.9. We consider two tissues colocalizing if they share a CAFEH component (p_active > 0.9).

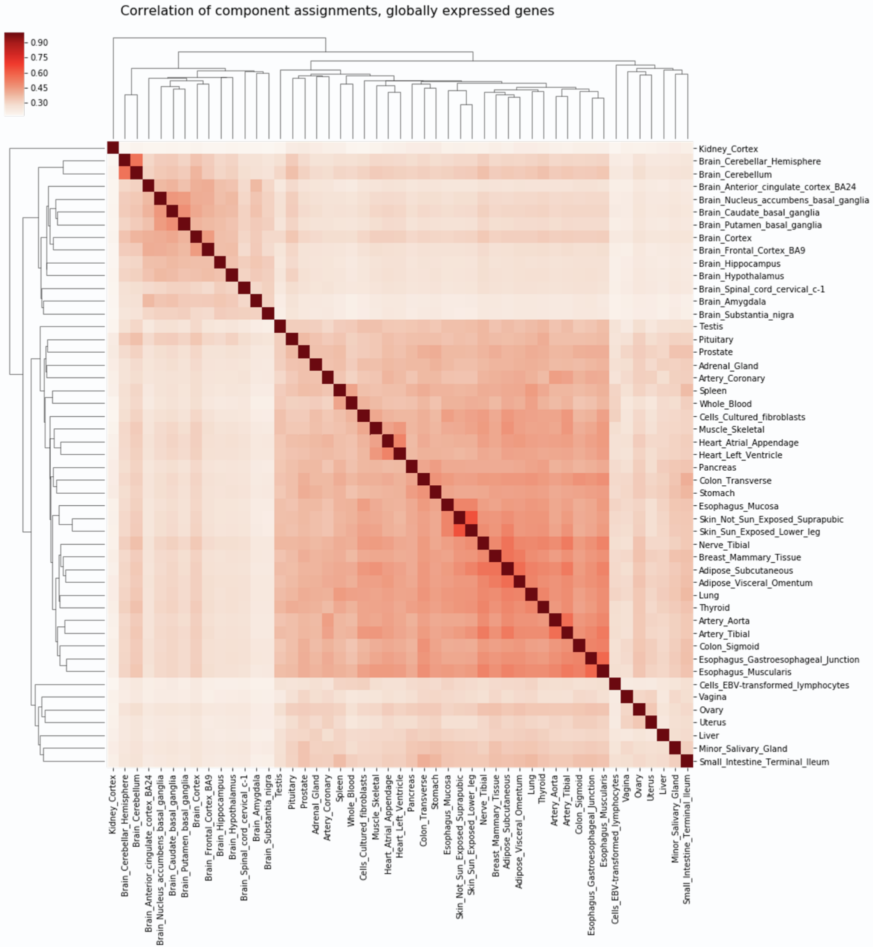

Figure S23: Correlation of CAFEH component activity across GTEx protein coding genes. Heatmap shows Pearson correlation of CAFEH component activity between GTEx tissues across 17,985 protein coding genes. Dendrogram denotes a hierarchical clustering of tissues. Similar tissues share more CAFEH components on average.

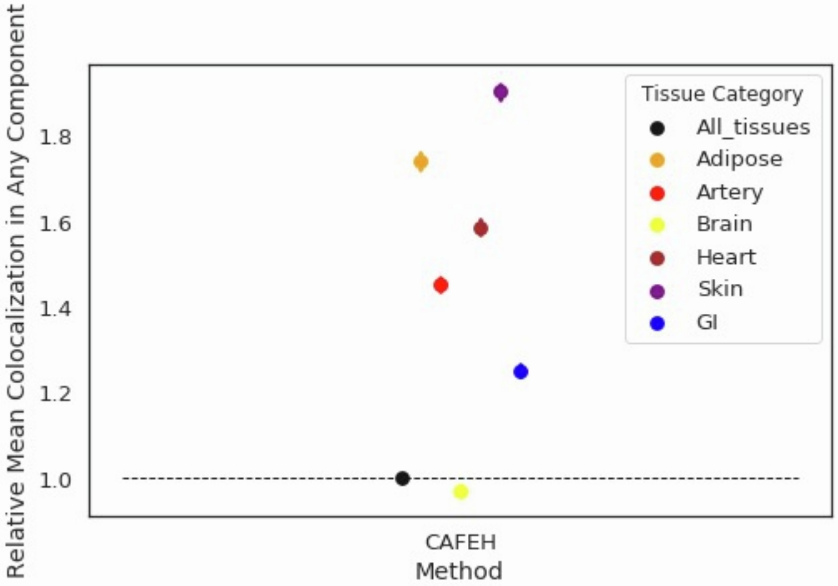

Figure S24: CAFEH reveals tissue specific colocalization of GTEx tissues. GTEx tissues are grouped into related tissues. For each tissue category, the the average of pairwise colocalization between tissues, calculated as $\max_{k=1\ldots K} \min(p_{t_1 k}, p_{t_2 k})$, is taken across 17,985 protein coding genes. Values are normalized to the average colocalization of all tissue pairs.

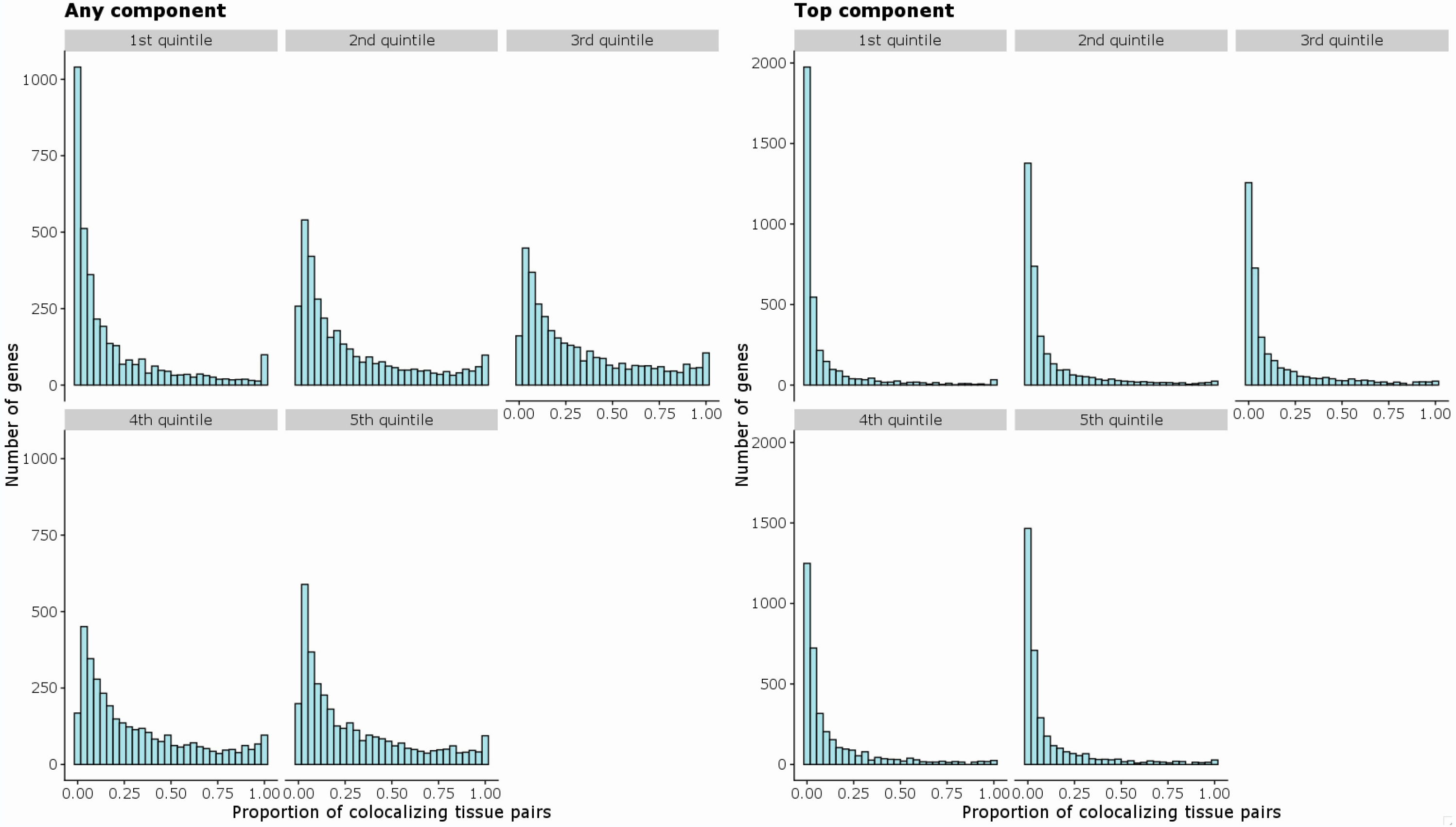

Figure S25. Influence of gene expression level on colocalization. All protein coding genes tested in at least one tissue in GTEx v8 (n=17601) were stratified into quintiles based on their median expression levels across tissues. Histograms of proportions of colocalizing tissue pairs are plotted for each expression quintile based on CAFEH colocalization in any component (left panel) or top component (right panel).

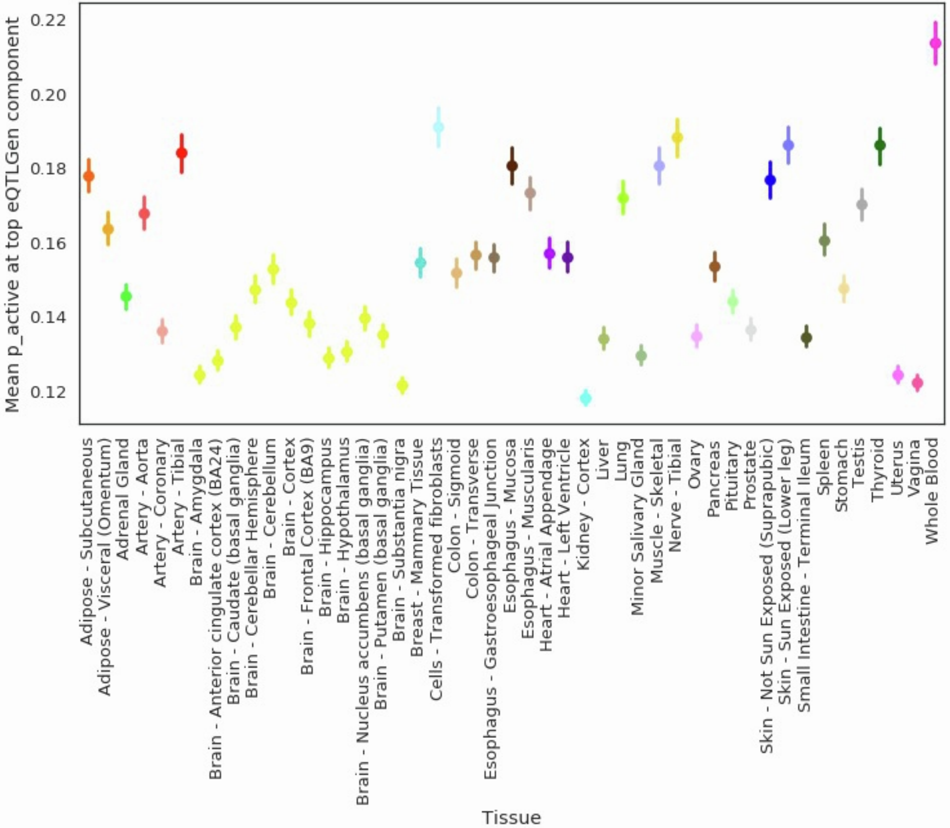

Figure S26: CAFEH colocalizes eQTLGen with relevant GTEx Tissues. CAFEH-S was run on cis-eQTL summary statistics from eQTLGen and 49 GTEx tissues for 9,744 protein coding genes. Plot shows average component activity (95% boostrap CI) for the top eQTLGen component in 49 GTEx tissues. We see highest average colocalization with GTEx Whole Blood.

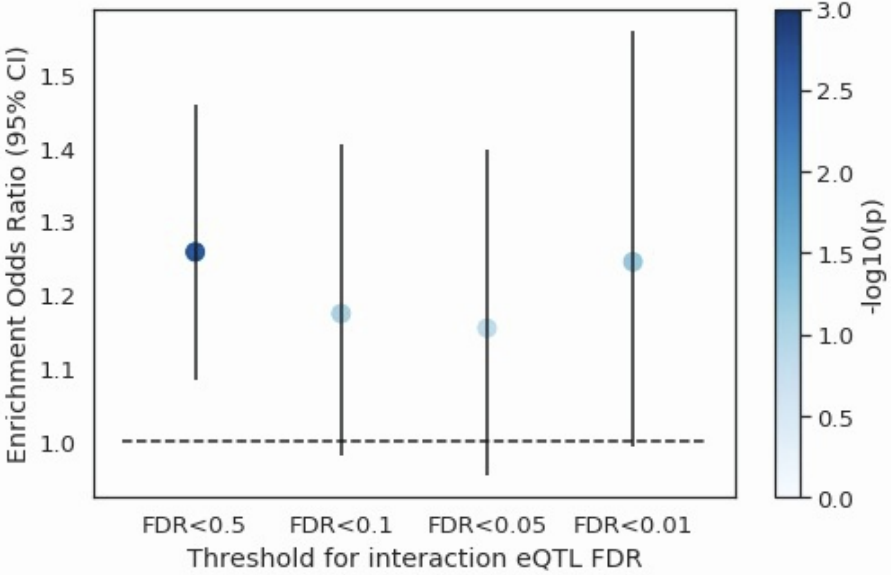

Figure S27: Enrichment of cell-type interacting genes in genes that do not colocalize in CAFEH. We consider GTEx Whole Blood and eQTLGen colocalizing if GTEx is active in the top eQTLGen component and both GTEx and eQTLGen have p_active > 0.9.

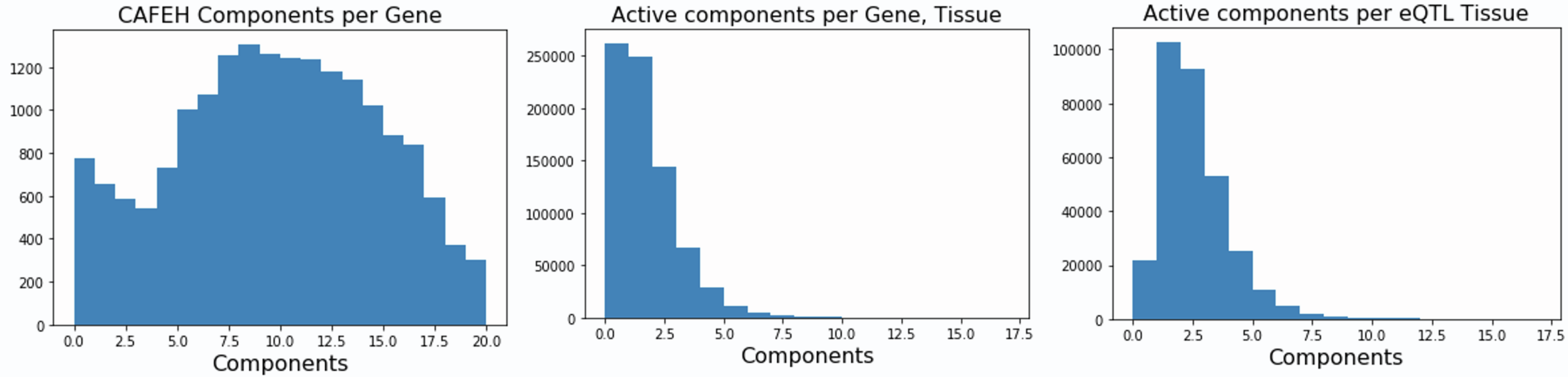

Figure S28: Extent of allelic heterogeneity in cis-eQTLs. A. Number of CAFEH components active in at least one tissue across GTEx v8 protein coding genes. **B.** Number of components per tissue across GTEx v8 protein coding genes. **C.** Number of components per tissue with a genome-wide significant eQTL across GTEx v8 protein coding genes.

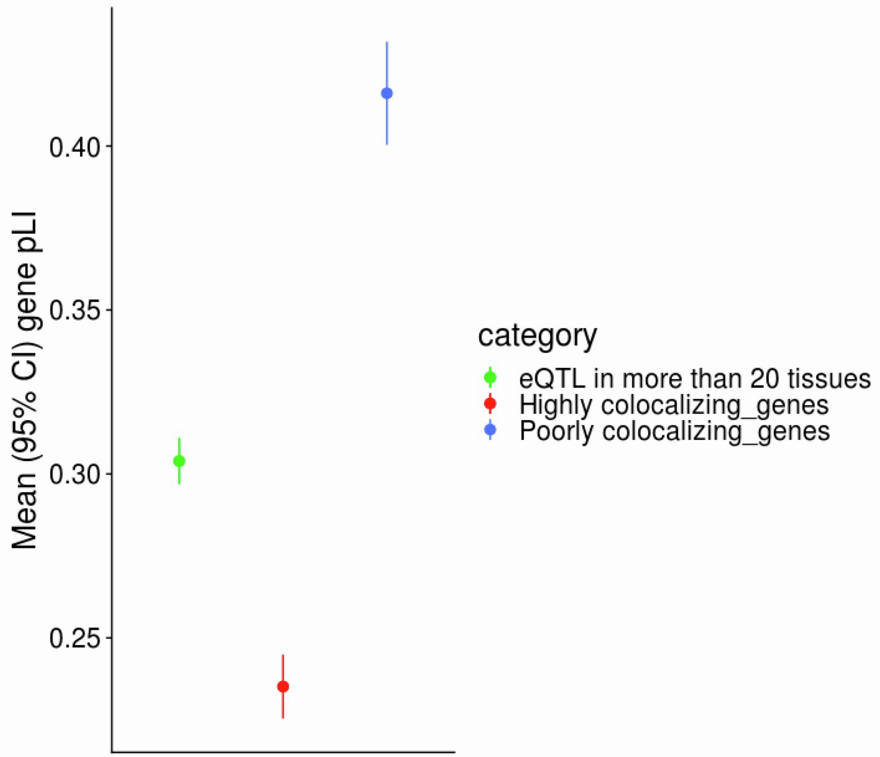

Figure S29. Gene LOEUF stratified by colocalization probability. Average probability of loss of function intolerance (pLI) between genes that are colocalizing in at least 20 tissues (highly colocalizing) and those that colocalize in less than 5 tissues (poorly colocalizing), comparing only genes that have an eQTL in at least 20 tissues. Colocalization was defined as sharing of the top causal component based on CAFEH**.** Similar to LOEUF, this alternative conservation metric also demonstrates higher conservation of genes that are poorly colocalizing according to CAFEH.

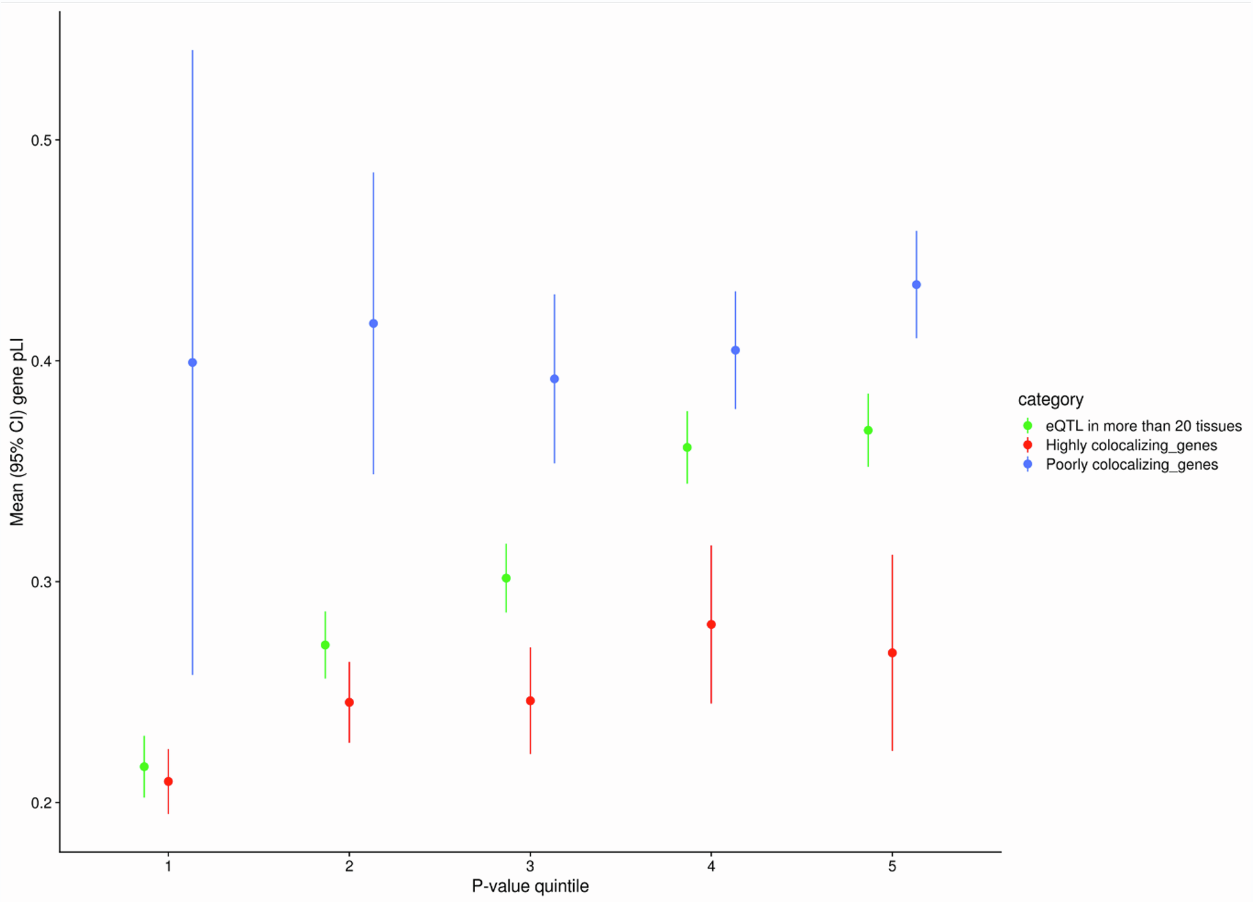

Figure S30: Average probability of loss of function intolerance (pLI) between genes that are colocalizing in at least 20 tissues (highly colocalizing) and those that colocalize in less than 5 tissues (poorly colocalizing) compared to all genes that have an eQTL in at least 20 tissues at different quantiles of the geometric average eQTL p-value of the strongest associated variant for each gene. Colocalization was defined as sharing of the top component based on CAFEH. We see that poorly colocalizing genes are more conserved compared to highly colocalizing genes in all quintiles.

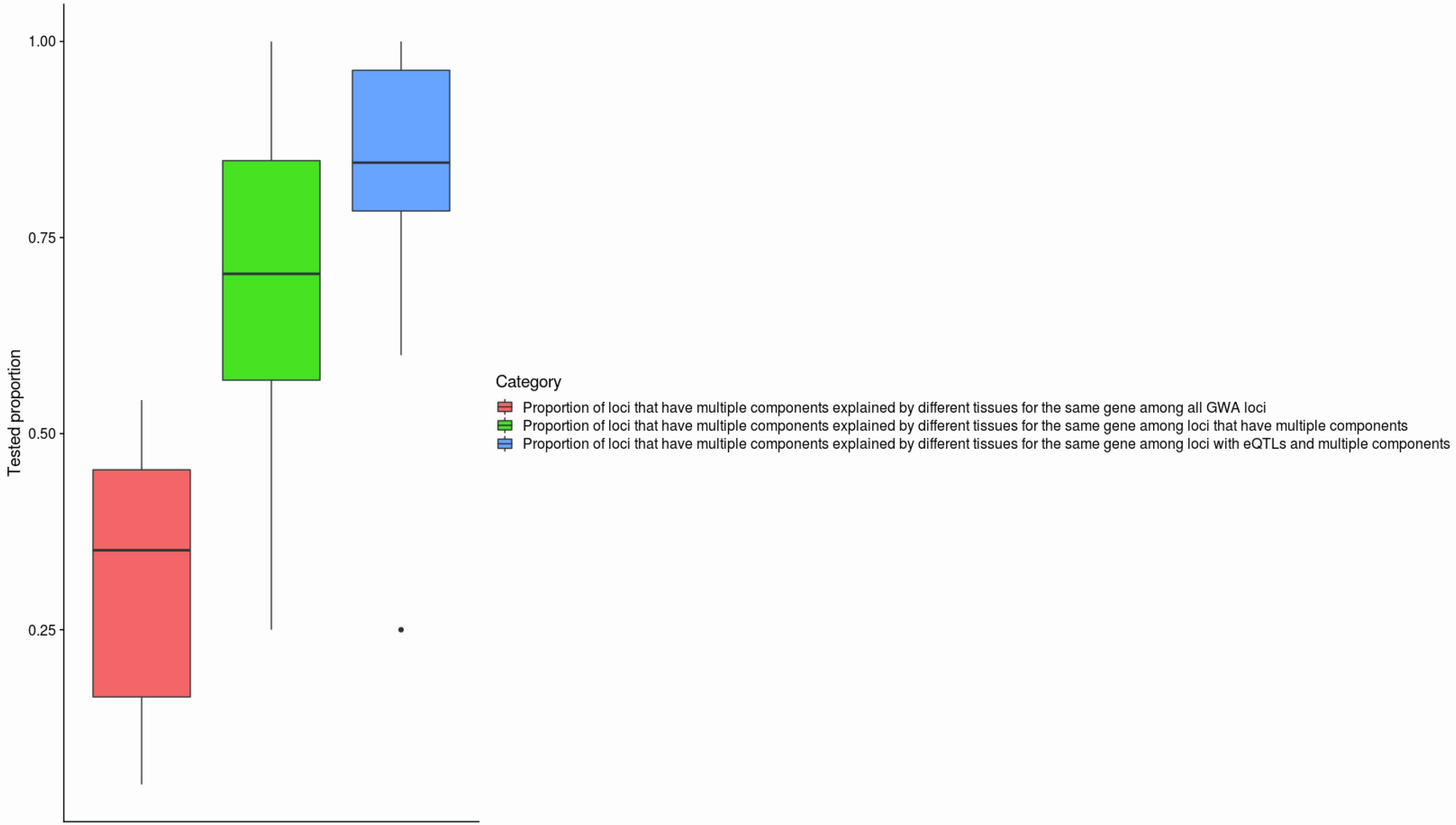

Figure S31: Proportion of loci in 19 UK Biobank GWAS traits that have multiple active components colocalizing with different tissues for the same gene based on CAFEH. The figure displays boxplots of the median proportions across the 19 tested GWAS traits. The red panel displays proportion of the loci that have the characteristics of the title divided by all genome-wide significant loci. The green panel displays the proportion divided by loci that have multiple components based on CAFEH. The blue panel displays the proportion divided by loci that have a genome-wide significant eQTL in at least one GTEx v8 tissue and also have multiple active components based on CAFEH. Colocalization was defined as p_active >=0.5 in both the GWAS and the tested tissue based on CAFEH.

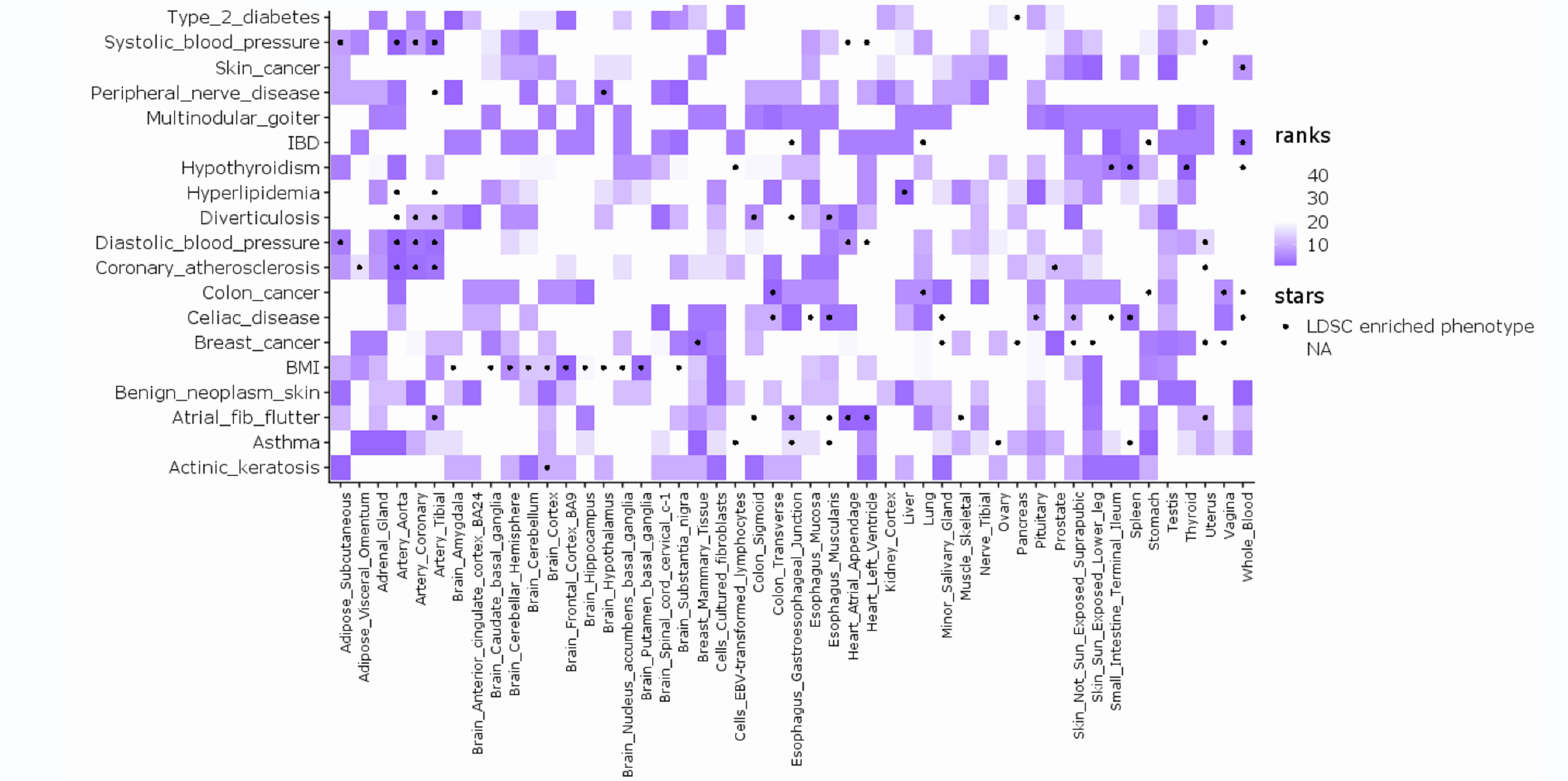

Figure S32: Heatmap of the prioritized tissues based on CAFEH for different UK Biobank GWAS traits. Tissues are colored based on their ranks which are determined based on the number of colocalizing loci based on CAFEH top component colocalization. Ranks range from 1-49 with 1 being the highest (most colocalizing) tissue. Tissues that are also enriched based on LD score regression are annotated. We see significant overlap in tissue prioritization between CAFEH and LDSC.

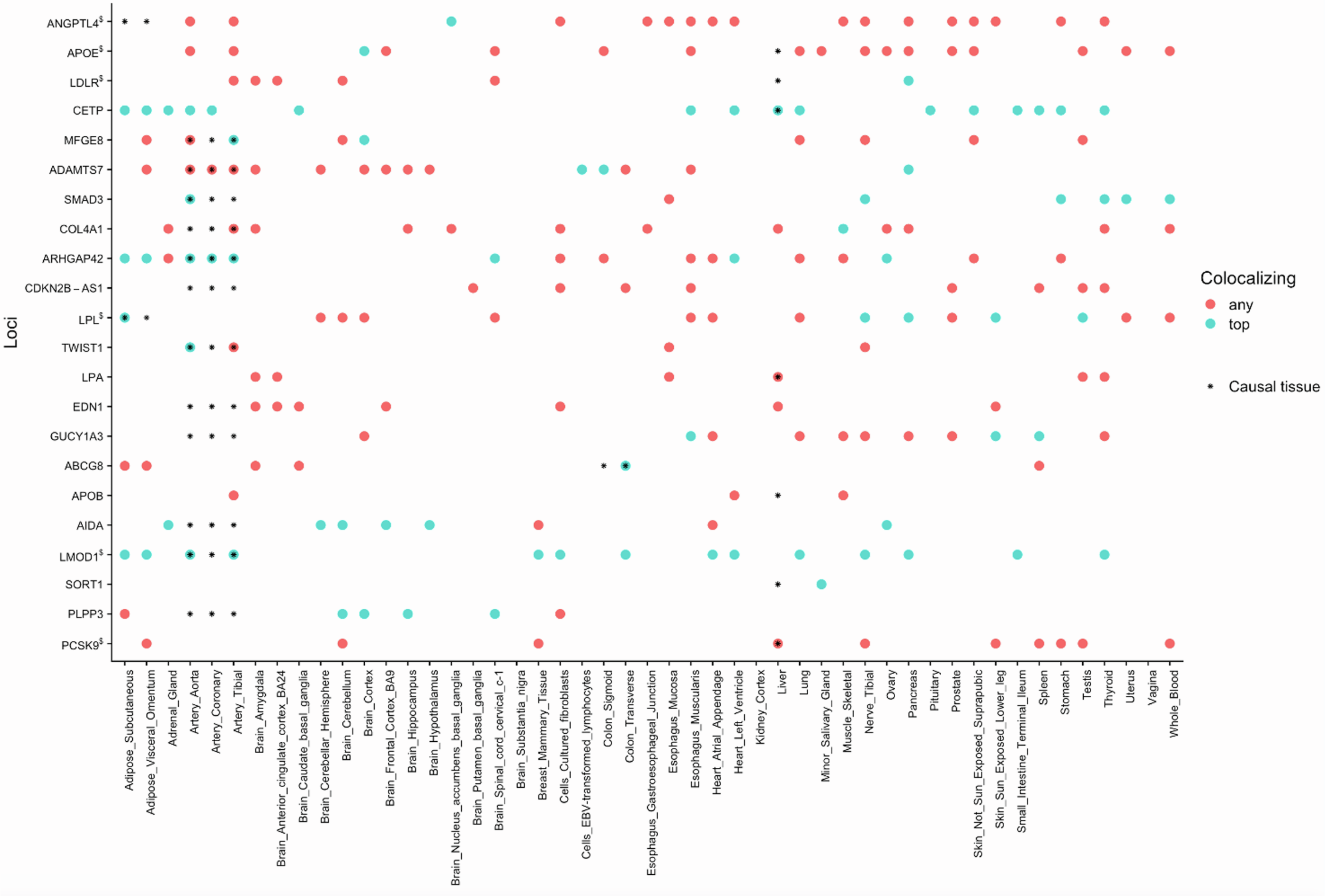

Figure S33: Colocalization of functionally characterized CAD GWAS loci in different GTEx v8 tissues based on CAFEH.

Supplemental Methods

In this document we review variational inference and describe the variational approximation used in CAFEH. Then we derive the coordinate ascent updates for CAFEH-G and CAFEH-S. Finally, we describe how to use stochastic variational inference to improve speed of CAFEH-S optimization.

1 Variational Inference Review

1.0.1 Problem set up

Given a model $p(Y, \theta)$ where Y are observed data and θ are latent variables, we want to compute the posterior distribution $p(\theta|Y)$. When the exact posterior distribution is intractable, we can approximate the posterior using variational inference.

In variational inference, we recast inference as an optimization problem. We posit a family of distributions $\mathcal{Q}$ over the latent variables in the model θ and find the member of that family that minimizes the KL-divergence to the true posterior.

$$q^*(\theta) = argmin_{q \in \mathcal{Q}} KL\left[q(\theta)||p(\theta|Y)\right] \tag{1}$$

When $p(\theta|Y) \in \mathcal{Q}$ this optimization yields the true posterior distribution. In practice, we choose $\mathcal{Q}$ so that we can efficiently optimize over the parameters of the family. Specifically it is often useful to choose a family of variational distributions that factorize over latent variables: $q(\theta) = \prod_i q(\theta_i)$.

We can solve this optimization by maximizing the Evidence Lower Bound (ELBO), which is a lower bound to the marginal data likelihood $p(Y|X) = \int_\theta p(Y, \theta|X) d\theta$

$$ELBO = \mathbb{E}_q\left[\ln p(Y, \theta|X)\right] + \mathbb{E}_q\left[\ln q(\theta)\right] \tag{2}$$

It can be shown that optimizing the ELBO with respect to the variational parameters is equivalent to minimizing the KL divergence in (1) [1].

The ELBO may be equivalently expressed as

$$ELBO = \mathbb{E}_q\left[p(Y|X, \theta)\right] - KL\left[q(\theta)||p(\theta)\right] \tag{3}$$

1.0.2 Deriving updates

We want to derive the udpate for a variational factor $q(z)$. where z is some subset of the latent variables in the model. Modifying the logic from [1] consider decomposing the ELBO

$$ELBO = \mathbb{E}_q(z)\left[\mathcal{L}\right] - \mathbb{E}_q(z)\left[\ln q(z)\right] + C \tag{4}$$

Where $\mathcal{L}$ are all terms of the ELBO that depend on z, and $q(z)$ is a density function which satisfies $\int q(z) = 1$. Using Lagrange multipliers to encode this constraint

$$\frac{d}{dq(z)} ELBO = \frac{d}{dq(z)}\left\{\mathbb{E}_{q(z)}\left[\mathbb{E}_{q(-z)}\left[\mathcal{L}\right]\right] - \mathbb{E}_{q(z)}\left[\ln q(z)\right] + \lambda \mathbb{E}_{q(z)}[1] - 1\right\} \tag{5}$$

$$= \mathbb{E}_{q(-z)}\left[\mathcal{L}\right] - \ln q(z) + \lambda \tag{6}$$

Setting the derivative equal to 0 we find

$$\ln q(z) = E_{q(-z)}\left[\mathcal{L}\right] + \lambda \tag{7}$$

Recognizing that $q(z)$ must integrate to one and that the normalizing factor does not depend on z

$$q^*(z) \propto exp\left\{\mathbb{E}_{q(-z)}\left[\mathcal{L}\right]\right\} \tag{8}$$

This suggests an approach for deriving our updates: compute $E_{q(-z)}\left[\mathcal{L}\right]$ and identify the parameters for $q(z)$ satisfying (8). Note that in general, identifying this distribution is not straight-forward. However, for a special class of models, of which CAFEH is a member, the coordinate-wise optima are exponential family distributions and their parameters can be computed analytically.

2 CAFEH-G

2.1 Model

For clarity we restat the model. Let Y an $N \times T$ matrix of measurements in N individuals across T phenotypes. Let X be a $N \times G$ matrix of genotypes in N individuals across G SNPs. The CAFEH model is written as

$$
\begin{aligned}
Y_t &\sim (X\mathbf{b}_t, \tau_t^{-1} I) && (9)\\
\mathbf{b}_t &= \sum_{k=1}^{K} \phi_k w_{tk} s_{tk} && (10)\\
w_{tk}|\alpha_{tk} &\sim \mathcal{N}(0, \alpha_{tk}^{-1}) && (11)\\
s_{tk} &\sim \text{Bernoulli}(p_{0k}) && (12)\\
\phi_k &\sim \text{Categorical}(\pi_0) && (13)\\
\alpha_{tk} &\sim \Gamma(a_0, b_0) && (14)\\
\tau_t &\sim \Gamma(c_0, d_0) && (15)
\end{aligned}
$$

2.2 Variational Approximation

Let $\theta = \{w_{tk}\} \cup \{s_{tk}\} \cup \{\phi_k\} \cup \{\alpha_{tk}\} \cup \{\tau_t\}$ denote the set of latent variables.

We select $\mathcal{Q}$ to factorize as follows:

$$
q^*(\theta) = \prod_k \prod_t q(w_{tk}|\phi_k, s_{tk}) q(s_{tk}) q(\alpha_{tk}) \prod_k q(\phi_k) \prod_t q(\tau_t) \tag{16}
$$

In particular we choose to a variational family that maintain dependence of w_{tk} on ϕ_k and s_{tk} so that we can accurately estimate effect sizes under different causal configurations. This is similar to the choice made in for the variational approximations chosen for SuSiE [3] and [2].

We optimize the ELBO via coordinate ascent, iteratively updating each $q(w|\phi, s)$, $q(\phi)$, $q(s)$, $q(\alpha)$ and $q(\tau)$, while holding the others fixed. Note, that while we have not specified a parametric form for the factors of the variational distribution, the model and factorization imply the optimal form of each variational factor:

$$
\begin{aligned}
q^*(s_{tk}) &\sim Bernoulli(\gamma_{tk})\\
q^*(\phi_k) &\sim Categorical(\pi_k)\\
q^*(\alpha_{tk}) &\sim \Gamma(a_{tk}, b_{tk})\\
q^*(\tau_t) &\sim \Gamma(c_t, d_t)\\
q^*(w_{tk}|\phi_k = i, s_{tk} = 1) &\sim \mathcal{N}(\mu_{tki}, \sigma^2_{tki})
\end{aligned}
\tag{17}
$$

$\{\mu, \sigma^2, \gamma, \pi, a, b, c, d\}$ (omitting subscripts) are *variational parameters* that we optimize over. We provide the full updates and their derivation below.

2.3 Evidence Lower Bound (ELBO)

$$ELBO = \mathbb{E}_{q(\theta)}\left[\ln p(\mathbf{Y}|\theta)\right] - KL\left[q(\theta)||p(\theta)\right] \tag{18}$$

$$\begin{aligned}
&= \mathbb{E}_{q(\theta)}\left[\sum_t \ln \mathcal{N}(\mathbf{Y}_t|\mathbf{b}_t, \tau^{-1}I)\right] \\
&- \sum_{t,k} \mathbb{E}_{q(s_{tk},\alpha_{tk},\phi_k)}\left[KL\left[q(w_{tk}|s_{tk},\phi_k)||p(w_{tk}|\alpha_{tk})\right]\right] \\
&- \sum_{t,k} KL\left[q(s_{tk})||p(s_{tk})\right] - \sum_{t,k} KL\left[q(\alpha_{tk})||p(\alpha_{tk})\right] \\
&- \sum_k KL\left[q(\phi_k)||p(\phi_k)\right] - \sum_t KL\left[q(\tau_t||p(\tau_t)\right]
\end{aligned} \tag{19}$$

2.3.1 Expected conditional

$$\begin{aligned}
&\mathbb{E}_{q(\theta)}\left[\ln \mathcal{N}(\mathbf{Y}_t|\mathbf{X}\mathbf{b}_t, \tau^{-1}I)\right] = \\
&\quad -\frac{M}{2}\ln 2\pi + \frac{M}{2}\langle \ln \tau_t\rangle - \frac{\langle \tau_t\rangle}{2}\left[\mathbf{Y}_t^T\mathbf{Y}_t - 2\mathbf{Y}_t^T\langle \mathbf{X}\mathbf{b}_t\rangle - \langle \mathbf{b}_t^T\mathbf{X}^T\mathbf{X}\mathbf{b}_t\rangle\right]
\end{aligned} \tag{20}$$

The expectation of $\mathbf{b}_t$ is

$$\langle \mathbf{b}_t\rangle = \sum_k (\pi_k \circ \mu_{tk})\gamma_{tk} \tag{21}$$

Letting $d_i = e_i^T\mathbf{X}^T\mathbf{X}e_i$ and $\langle \mathbf{b}_{tk}\rangle = (\pi_k \circ \mu_{tk})\gamma_{tk}$ and noting $s_{tk}^2 = s_{tk}$ we can get a nice expression for the quadratic term

$$\langle \mathbf{b}_t^T\mathbf{X}^T\mathbf{X}\mathbf{b}_t\rangle = \left\langle \left(\sum_k \phi_k w_{tk} s_{tk}\right)^T \mathbf{X}^T\mathbf{X}\left(\sum_k \phi_k w_{tk} s_{tk}\right)\right\rangle \tag{22}$$

$$= \sum_k \langle w_{tk}^2 s_{tk} d_{\phi_k}\rangle + \sum_{k\neq j}\langle w_{tk}s_{tk}\phi_k^T\rangle \mathbf{X}^T\mathbf{X}\langle \phi_j w_{tj} s_{tj}\rangle \tag{23}$$

$$= \sum_{k,i}(\mu_{tki}^2 + \sigma_{tki}^2)\gamma_{tk}\pi_{ki}d_i + \langle \mathbf{b}_t\rangle^T\mathbf{X}^T\mathbf{X}\langle \mathbf{b}_t\rangle - \sum_k ||\mathbf{X}\langle \mathbf{b}_{tk}\rangle||^2 \tag{24}$$

2.3.2 KL computations

To compute the ELBO and coordinate ascent updates, we need to compute $\mathbb{E}\left[KL\left[q(w|\phi,s)||p(w|\alpha)\right]\right]$, where expectations are taken over $q(\alpha)$, $q(s_{tk})$ and/or $q(\phi_k)$ depending on the setting. s and ϕ appear linearly, while α does not. Here we write the expectation of the KL divergence w.r.t α in terms of the the KL of the expectation plus a positive correction.

$$\begin{aligned}
&\left\langle KL\left[\mathcal{N}(\mu,\sigma^2)\,||\mathcal{N}(0,\alpha^{-1})\right.\right. && (25)\\
&= \left\langle \frac{1}{2}\left[\alpha\mu^2+\sigma^2\alpha-1-\ln\sigma^2-\ln\alpha\right]\right\rangle && (26)\\
&= \frac{1}{2}\left[\langle\alpha\rangle\mu^2+\sigma^2\langle\alpha\rangle-1-\ln\sigma^2-\langle\ln\alpha\rangle\right] && (27)\\
&= \frac{1}{2}\left[\langle\alpha\rangle\mu^2+\sigma^2\langle\alpha\rangle-1-\ln\sigma^2-\ln\langle\alpha\rangle\right]+\frac{1}{2}(\ln\langle\alpha\rangle-\langle\ln\alpha\rangle) && (28)\\
&= KL\left[\mathcal{N}(\mu,\sigma^2)||\mathcal{N}(0,\langle\alpha\rangle^{-1})\right]+\frac{1}{2}(\ln\langle\alpha\rangle-\langle\ln\alpha\rangle) && (29)
\end{aligned}$$

2.3.3 Residualized likelihood

As we write our variational updates it will be useful to define $r_{tk} = Y_t - X\mathbf{b}_t + X\mathbf{b}_{tk}$ where $\mathbf{b}_{tk} = \phi_k w_{tk} s_{tk}$. That is, r_{tk} is the residual with all but the k-th component removed. The conditional likelihood may be written

$$\mathcal{N}(Y_t|X\mathbf{b}_t,\tau_t^{-1}) = \mathcal{N}(r_{tk}|X\mathbf{b}_{tk},\tau_t^{-1}) \qquad (30)$$

Then, when considering updates for a particular component k, we can write the ELBO as

$$ELBO = \mathbb{E}_{q(\theta)}\left[\sum_t -\frac{\tau_t}{2}\left[-2r_{tk}^T\mathbf{X}\mathbf{b}_{tk}+\mathbf{b}_{tk}^T\mathbf{X}^T\mathbf{X}\mathbf{b}_{tk}\right]\right] - KL\left[q(\theta)||p(\theta)\right] \qquad (31)$$

2.4 Coordinate Ascent updates

2.4.1 Update for $q^*(w_{tk}|\phi_k = i, s_{tk} = 1$

Where $\mathbf{x}_i$ is the ith column of X, the genotypes at SNP i.

$$\begin{aligned}
&q^*(w_{tk}|s_{tk}=1,\phi_k=i) && (32)\\
&\propto exp\left\{\left\langle\ln\mathcal{N}(r_{tk}|w_{tk}\mathbf{x}_i,\tau_t^{-1}\mathbf{I})\right\rangle+\langle\ln p(w_{tk}|\alpha_{tk})\rangle\right\} && (33)\\
&\propto exp\left\{\frac{\langle\tau_t\rangle}{2}\left(-2\langle r_{tk}\rangle^T\mathbf{x}_i w_{tk}+d_i w_{tk}^2\right)+\frac{\langle\alpha_{tk}\rangle}{2}(w_{tk}^2)\right\} && (34)
\end{aligned}$$

Completing the square we find

$$\begin{aligned}
\sigma_{tki}^2 &= (d_i\langle\tau_t\rangle+\langle\alpha_{tk}\rangle)^{-1} && (35)\\
\mu_{tki} &= \sigma_{tki}^2\langle\tau_t\rangle\langle r_{tk}\rangle^T\mathbf{x}_i && (36)\\
q^*(w_{tk}|\phi_k=i,s_{tk}=1) &= \mathcal{N}(w_{tk}|\mu_{tki},\sigma_{tki}^2) && (37)
\end{aligned}$$

2.4.2 Update for $q^*(w_{tk}|\phi_k, s_{tk} = 0)$

$$
\begin{aligned}
&q^*(w_{tk}|s_{tk} = 1, \phi_k = i) \\
&\qquad \propto exp\left\{\left\langle \ln \mathcal{N}(r_{tk}|0, \tau_t^{-1}\mathbf{I})\right\rangle + \langle \ln p(w_{tk}|\alpha_{tk})\rangle\right\} \\
&\qquad \propto exp\left\{\frac{\langle \alpha_{tk}\rangle}{2}(w_{tk}^2)\right\}
\end{aligned} \tag{38}
$$

$$
q^*(w_{tk}|s_{tk} = 0, \phi_k = i) = \mathcal{N}(w + tk|0, \langle \alpha_{tk}\rangle^{-1}) \;\; \forall i \in \{1, \ldots, N\} \tag{39}
$$

2.4.3 Update for $q^*(s_{tk})$

$$
\begin{aligned}
q^*(s_{tk}) \propto exp\{ &\left\langle \ln \mathcal{N}(r_{tk}|\mathbf{X}\phi_k w_{tk}, \tau_t^{-1}\mathbf{I})\right\rangle \mathbb{1}(s_{tk} = 1) \\
&+ \langle KL\left[q(w_{tk}, \alpha_{tk}|s_{tk} = 1, \phi_k)||p(w_{tk}, \alpha_{tk})\right]\rangle \mathbb{1}(s_{tk} = 1) \\
&+ \ln p_{0k}\mathbb{1}(s_{tk} = 1) \\
&\left\langle \ln \mathcal{N}(r_{tk}|0, \tau_t^{-1}\mathbf{I})\right\rangle \mathbb{1}(s_{tk} = 0) \\
&+ \langle KL\left[q(w_{tk}, \alpha_{tk}|s_{tk} = 0, \phi_k)||p(w_{tk}, \alpha_{tk})\right]\rangle \mathbb{1}(s_{tk} = 0) \\
&+ \ln(1 - p_{0k})\mathbb{1}(s_{tk} = 0)
\end{aligned} \tag{40}
$$

Grouping terms where $s_{tk} = 1$ and $s_{tk} = 0$ we can write

$$
q^*(s_{tk}) \propto exp\left\{(a + \ln p_{0k})\mathbb{1}(s_{tk} = 1) + (b + \ln(1 - p_{0k}))\mathbb{1}(s_{tk=0})\right\} \tag{41}
$$

$$
\begin{aligned}
a = -\frac{\langle \tau_t\rangle}{2}&\left[-2\langle r_{tk}\rangle^T \mathbf{X}(\pi_k \circ \mu_{tk}) + \sum_i (\mu_{tki}^2 + \sigma_{tki}^2)\pi_{ki}\right] \\
&- \sum_i \pi_{ki}\langle KL\left[q(w_{tk}, \alpha_{tk}|s_{tk} = 1, \phi_k = i)||p(w_{tk}, \alpha_{tk}\right]\rangle
\end{aligned} \tag{42}
$$

$$
b = -\langle KL\left[q(w_{tk}, \alpha_{tk}|s_{tk} = 0)||p(w_{tk}, \alpha_{tk}\right]\rangle = -\frac{1}{2}(\ln\langle \alpha\rangle - \langle \ln \alpha\rangle) \tag{43}
$$

Setting $\gamma_{tk} = \frac{e^a p_{0k}}{e^a p_{0k} + e^b(1 - p_{0k})}$

$$
q^*(s_{tk}) = \text{Bernoulli}(s_{tk}|\gamma_{tk}) \tag{44}
$$

2.4.4 Update for $q^*(\alpha_{tk})$

$$\begin{aligned} q^*(\alpha_{tk}) &\propto exp\left\{\left\langle \ln \mathcal{N}(w_{tk}|0, \alpha_{tk}^{-1}) \ln p(\alpha_{tk})\right\rangle\right\} \\ &\propto exp\left\{\frac{1}{2}\ln\alpha_{tk} - \frac{\alpha_{tk}}{2}\left\langle w_{tk}^2\right\rangle + (a_0-1)\ln\alpha_{tk} - b_0\alpha_{tk}\right\} \\ &\propto exp\left\{\left(a_0 + \frac{1}{2} - 1\right)\ln\alpha_{tk} - \left(b_0 + \frac{\left\langle w_{tk}^2\right\rangle}{2}\right)\alpha_{tk}\right\} \\ &\propto exp\left\{\left(a_0 + \frac{1}{2} - 1\right)\ln\alpha_{tk} - \left(b_0 + \frac{\sum_i \pi_{ki}(\mu_{tki}^2 + \sigma_{tki})^2}{2}\right)\alpha_{tk}\right\} \end{aligned} \tag{45}$$

Let $a = a_0 + \frac{1}{2}$ and $b = b_0 + \frac{\sum_i \pi_{ki}(\mu_{tki}^2+\sigma_{tki})^2}{2}$

$$q^*(\alpha_{tk}) = \Gamma(\alpha_{tk}|a, b) \tag{46}$$

2.4.5 Update for $q^*(\phi_k)$

$$q^*(\phi_k) \propto \sum_i \rho_{ki} 1(\phi_k = i) \tag{47}$$

$$\begin{aligned} \rho_{ki} =& \left\langle \ln \mathcal{N}(r_{tk}|s_{tk}w_{tk}\mathbf{x}_i, \tau^{-1}I\right\rangle \\ &- \langle KL[q(w_{tk}, \alpha_{tk}|\phi_k = i) \mid\mid p(w_{tk}|\alpha_{tk})]\rangle + \ln\pi_{0ki} \end{aligned} \tag{48}$$

$$\begin{aligned} \rho_{ki} =& -\frac{\langle\tau_t\rangle}{2}\left[-2\left\langle r_{tk}\right\rangle^T \mathbf{x}_i\mu_{tk}\gamma_{tk} + \gamma_{tk}(\mu_{tki}^2 + \sigma_{tki}^2)\right] \\ &- \langle KL[q(w_{tk}, \alpha_{tk}|s_{tk} = 1, \phi_k = i) \mid\mid p(w_{tk}|\alpha_{tk})]\rangle\,\gamma_{tk} \\ &- \langle KL[q(w_{tk}, \alpha_{tk}|s_{tk} = 0, \phi_k = i) \mid\mid p(w_{tk}|\alpha_{tk})]\rangle\,(1-\gamma_{tk}) + \ln\pi_{0ki} \end{aligned} \tag{49}$$

Then

$$\pi_{ki} = \frac{e^{\rho_i}}{\sum_i e^{\rho_{ik}}} \tag{50}$$

2.4.6 Update for $q^*(\tau_t)$

$$\begin{aligned} \ln q^*(\tau_t) &\propto \left\langle \mathcal{N}(\hat{\beta}_t|\mathbf{X}\mathbf{b}_t, \tau_t^{-1}I) + \ln p(\tau_t)\right\rangle \\ &\propto \frac{1}{2}\ln\tau_t - \frac{\tau_t}{2}\left\langle (\hat{\beta}_t - \mathbf{X}\mathbf{b}_t)^T(\hat{\beta}_t - \mathbf{X}\mathbf{b}_t)\right\rangle + (c_0-1)\ln\tau_t - d_0\tau_t \end{aligned} \tag{51}$$

Let $c = c_0 + \frac{1}{2}$ and $d = d_0 + \frac{\left\langle(\hat{\beta}_t - \mathbf{X}\mathbf{b}_t)^T(\hat{\beta}_t - \mathbf{X}\mathbf{b}_t)\right\rangle}{2}$

$$q^*(\tau_t) = \Gamma(\tau_t|c, d) \tag{52}$$

3 CAFEH-S model

CAFEH-S has an identical prior on the effect sizes $\mathbf{b}_t$ as CAFEH-G, however the likelihood is written in terms of summary statistics using the RSS likelihood [4]. $\hat{\beta}_t$ are the vector of effect sizes for marginal linear regression of G SNPs in phenotype t. R is an LD matrix containing the pairwise correlation of SNPs. S is a diagonal matrix where $S_{ii}^2 = \beta^2/n_{ti} + \hat{s}^2 + ti$. n_{ti} and $\hat{s}_{ti}$ are the sample size and standard errors for the corresponding tests.

$$\hat{\beta}_t \sim (SRS^{-1}\mathbf{b}_t, SRS) \tag{53}$$

$$\mathbf{b}_t = \sum_{k=1}^{K} \phi_k w_{tk} s_{tk} \tag{54}$$

$$w_{tk}|\alpha_{tk} \sim \mathcal{N}(0, \alpha_{tk}^{-1}) \tag{55}$$

$$s_{tk} \sim \text{Bernoulli}(p_{0k}) \tag{56}$$

$$\phi_k \sim \text{Categorical}(\pi_0) \tag{57}$$

$$\alpha_{tk} \sim \Gamma(a_0, b_0) \tag{58}$$

3.1 Evidence Lower Bound (ELBO)

We write the ELBO, lumping terms that are constant w.r.t the variational parameters into a constant C. Letting

$$D = S^{-1}RS^{-1}$$

$$ELBO = \mathbb{E}_q\left[\sum_t ln\mathcal{N}(\hat{\beta}_t|SRS^{-1}\mathbf{b}_t, SRS)\right] - KL\left[q||p\right] \tag{59}$$

$$= \mathbb{E}_q\left[\sum_t -\frac{1}{2}\left(-2\hat{\beta}_t^T S^{-2}\mathbf{b}_t + \mathbf{b}_t^T D\mathbf{b}_t\right)\right] - KL\left[q||p\right] + C \tag{60}$$

3.1.1 Residualized likelihood

Our coordinate ascent updates are performed by updating one component while holding all other components and fixed. It will be convenient to rewrite the likelihood in terms of the residual with all but one component removed

$$\mathbf{b}_{tk} = w_{tk}s_k\phi_k \tag{61}$$

$$\mathbf{b}_{-tk} = \sum_{j\neq k}\mathbf{b}_{tj} \tag{62}$$

$$r_{tk} = \hat{\beta}_t - SRS^{-1}\mathbf{b}_{-tk} \tag{63}$$

So that

$$\mathcal{N}(\hat{\beta}_t|SRS^{-1}\mathbf{b}_t, SRS) = \mathcal{N}(r_{tk}|SRS^{-1}\mathbf{b}_{tk}, SRS) \tag{64}$$

Notice that the term $r_{tk}^T(SRS)^{-1}r_{tk}$ does not depend on component k. For the purpose of optimization of the variational parameters of component k we may write the ELBO

$$ELBO = \mathbb{E}_q\left[\sum_t -\frac{1}{2}\left(-2r_{tk}^T S^{-2}\mathbf{b}_t + \mathbf{b}_t^T D\mathbf{b}_t\right)\right] - KL\left[q||p\right] + C \tag{65}$$

3.2 Coordinate Ascent updates

3.2.1 Update for $q^*(w_{tk}|\phi_k, s_{tk} = 1)$

With $d_i = D_{ii}$

$$\begin{aligned} q^*(w_{tk}|s_{tk} = 1, \phi_k = i) \propto \\ exp\left\{\left\langle \ln\mathcal{N}(r_{tk}|SRS^{-1}\mathbf{b}_{tk}, SRS) + \ln\mathcal{N}(w_{tk}|0,\alpha_{tk})\right\rangle\right\} \\ exp\left\{-\frac{1}{2}\left[-2\left\langle r_{tk}\right\rangle^T S^{-2}e_i w_{tk} + d_i w_{tk}^2 + \left\langle \alpha_{tk}\right\rangle w_{tk}^2\right]\right\} \end{aligned} \tag{66}$$

Completing the square we arrive at

$$\begin{aligned} \sigma_{tki}^2 = (d_i + \langle\alpha\rangle)^{-1} \\ \mu_{tki} = \sigma_{tki}^2 \left\langle r_{tk}\right\rangle^T S^{-2}e_i \\ q^*(w_{tk}|\phi_k = i, s_{tk} = 1) = \mathcal{N}(w_{tk}|\mu_{tki}, \sigma_{tki}^2) \end{aligned} \tag{67}$$

3.2.2 Update for $q^*(w_{tk}|\phi_k, s_{tk} = 0)$

$$q^*(w_{tk}|s_{tk} = 0, \phi_k = i) \propto exp\left\{-\frac{1}{2}\left\langle\alpha_{tk}\right\rangle w_{tk}^2\right\} \tag{68}$$

It follows that

$$q^*(w_{tk}|\phi_k, s_{tk} = 0) = \mathcal{N}(w_{tk}|0, \left\langle\alpha_{tk}\right\rangle^{-1}) \tag{69}$$

3.2.3 Update for $q^*(s_{tk})$

We group terms of the ELBO where $s_{tk} = 1$:

$$\begin{aligned} a = \mathbb{E}_{q|s_{tk}=1}\left[\log\mathcal{N}(r_{tk}|SRS^{-1}b_{tk}, SRS)\right] \\ +\mathbb{E}_{q(w_{tk},\phi_k,s_{tk}=1)}\left[\log p(w_{tk}|\alpha_{tk})\right] \\ +\mathbb{E}_{q(\phi_k)}\left[H(q(w_{tk}|s_{tk} = 1, \phi_k)\right] + \log p_{0k} + C \end{aligned} \tag{70}$$

Evaluates to

$$
\begin{aligned}
a = -\frac{1}{2}\left(-2\left\langle r_{tk}\right\rangle^{T} S^{-2}(\pi_k \circ \mu_{tk}) + \sum_i (\mu_{tki}^2 + \sigma_{tki}^2) d_i \pi_{ki}\right) \\
+\mathbb{E}_{q(w_{tk},\phi_k,s_{tk}=1)}\left[\log p(w_{tk}|\alpha_{tk})\right] \\
+\mathbb{E}_{q(\phi_k)}\left[H(q(w_{tk}|s_{tk}=1,\phi_k)\right] + \log p_{0k} + C
\end{aligned}
\tag{71}
$$

And $s_{tk} = 0$:

$$
\begin{aligned}
b = \mathbb{E}_{q|s_{tk}=0}\left[\log \mathcal{N}(r_{tk}|SRS^{-1}b, SRS)\right] \\
+\mathbb{E}_{q(w_{tk},\phi_k,s_{tk}=0)}\left[\log p(w_{tk}|\alpha_{tk})\right] + \\
\mathbb{E}_{q(\phi_k)}\left[H(q(w_{tk}|s_{tk}=0,\phi_k)\right] + \log(1-p_{0k}) + C
\end{aligned}
\tag{72}
$$

Evaluates to

$$
\begin{aligned}
b = 0 \\
+\mathbb{E}_{q(w_{tk},\phi_k,s_{tk}=0)}\left[\log p(w_{tk}|\alpha_{tk})\right] \\
+\mathbb{E}_{q(\phi_k)}\left[H(q(w_{tk}|s_{tk}=0,\phi_k)\right] + \\
\log(1-p_{0k}) + C
\end{aligned}
\tag{73}
$$

$$
q^*(s_{tk}) \propto exp\left\{a1(s_{tk}=1) + b1(s_{tk}=0)\right\} \implies \gamma_{tk} = \frac{e^a}{e^a + e^b}
\tag{74}
$$

3.2.4 Update for $q^*(\phi_k)$

Grouping terms where $\phi_k = i$

$$
\begin{aligned}
a_i = \mathbb{E}_{q|\phi_k=i}\left[\log \mathcal{N}(r_{tk}|SRS^{-1}b_{tk}, SRS)\right] \\
+\mathbb{E}_{q(w_{tk},s_{tk}|\phi_k=i)}\left[p(w_{tk}|\alpha_{tk})\right] \\
+\mathbb{E}_{q(s_{tk})}\left[H(q(w_{tk}|s_{tk},\phi_k=i)\right]
\end{aligned}
\tag{75}
$$

$$
\begin{aligned}
a_i = -\frac{1}{2}\left[-2\left\langle r_{tk}\right\rangle^{T} S^{-2} e_i \mu_{tki}\gamma_{tk} + \gamma_{tk}(\mu_{tk}^2 + \sigma_{tki}^2) d_i\right] \\
+\mathbb{E}_{q(w_{tk},s_{tk}|\phi_k=i)}\left[p(w_{tk}|\alpha_{tk})\right] \\
+\mathbb{E}_{q(\phi_k)}\left[H(q(w_{tk}|s_{tk},\phi_k=i)\right]
\end{aligned}
\tag{76}
$$

$$
q^*(s_{tk}) \propto exp\left\{\sum_i a_i 1(\phi_k = i)\right\} \implies \pi_{ki} = \frac{e^{a_i}}{\sum_i e^{a_i}}
\tag{77}
$$

3.3 Stochastic Variational Inference

3.3.1 Monte-Carlo estimate of the ELBO

Recall the ELBO for CAFEH-S

$$ELBO = \mathbb{E}_q\left[\sum_t -\frac{1}{2}\left(-2r_{tk}^T S^{-2}\mathbf{b}_t + \mathbf{b}_t^T D\mathbf{b}_t\right)\right] - KL\left[q||p\right] + C \tag{78}$$

The CAFEH-S updates, (equivalently, evaluating the gradient of the ELBO), require the repeated evaluation of $\langle r_{tk}\rangle = \hat{\beta}_t - SRS^{-1}\langle\mathbf{b}_{-tk}\rangle$. This involves a matrix-vector multiplication that grows with the number of SNPs, and causes CAFEH-S to be slow to run with a large number of variants.

We propose using a Monte-Carlo estimate for the expectation over $q(\phi)$. Rather than averaging over all SNPs, and incurring the expensive matrix-vector multiplication, we sample SNPs. We write $\mathbf{b}_{tk}(\phi_k)$ to emphasize the dependence of $\mathbf{b}_{tk}$ on ϕ_k.

$$\mathbb{E}_{q(\phi_k)}\left[\mathbb{E}_{q(-\phi_k)}\mathbf{b}_{tk}(\phi_k)\right] \approx \frac{1}{L}\sum_{l=1}^{L}\mathbb{E}_{q(-\phi_k)}\mathbf{b}_{tk}(z_k^{(l)}) = \tilde{\mathbf{b}}_{tk} \tag{79}$$

Where $z_k^{(1)}, \ldots, z_k^{(L)}$ are iid samples from $Categorical(\pi_k)$, the current setting of $q(\phi_k)$. This approximation yields a noisy but unbiased estimate of the ELBO, satisfying the core requirement for performing stochastic optimization.

Importantly for moderate choice of L, $LK << G$. Thus, $\tilde{\mathbf{b}}_t$ is sparse and $SRS^{-1}\tilde{\mathbf{b}}_{tk}$ can be computed quickly.

3.3.2 Stochastic Variational Inference

For models where all the complete conditionals are an exponential family, coordinate ascent on stochastic estimates of the ELBO is stochastic gradient ascent (in the natural parameter space) [cite]. In short, we can use the same updates as above, replacing expectations over $q(\phi_k)$ with their Monte-Carlo estimate, to compute $\hat{\lambda}$ an intermediate estimate of our variational parameter λ. We update our estimate of λ as a weighted average of our old estimate and the intermediate estimate

$$\lambda^{(t+1)} = (1-\rho_t)\lambda_t + \rho_t\hat{\lambda}_t \tag{80}$$

Where t indicates iteration, and ρ_t are weights. When the sequence $(\rho_t)_{t=1}^{\infty}$ satisfy the Robbins Monro conditions $\sum \rho_t = \infty$ and $\sum \rho_t^2 < \infty$, the stochastic optimization is guaranteed to converge to a local optimum.

We note that for well behaved causal components, where $q(\phi_k)$ places most of its mass on a set of tightly linked SNPs, the Monte-Carlo estimate will be very close to the true expectation.

References

[1] David M Blei, Alp Kucukelbir, and Jon D McAuliffe. Variational inference: A review for statisticians. *Journal of the American statistical Association*, 112(518):859–877, 2017.

[2] Michalis Titsias and Miguel Lázaro-Gredilla. Spike and slab variational inference for multi-task and multiple kernel learning. *Advances in neural information processing systems*, 24:2339–2347, 2011.

[3] Gao Wang, Abhishek Sarkar, Peter Carbonetto, and Matthew Stephens. A simple new approach to variable selection in regression, with application to genetic fine mapping. *Journal of the Royal Statistical Society: Series B (Statistical Methodology)*, 82(5):1273–1300, 2020.

[4] Xiang Zhu and Matthew Stephens. Bayesian large-scale multiple regression with summary statistics from genome-wide association studies. *The annals of applied statistics*, 11(3):1561, 2017.
